# Impacts for health and care workers of Covid-19 and other public health emergencies of international concern: living systematic review, meta-analysis and policy recommendations

**DOI:** 10.1186/s12960-024-00892-2

**Published:** 2024-01-25

**Authors:** Inês Fronteira, Verona Mathews, Ranailla Lima Bandeira dos Santos, Karen Matsumoto, Woldekidan Amde, Alessandra Pereira, Ana Paula Cavalcante de Oliveira, Isabel Craveiro, Raphael Chança, Mathieu Boniol, Paulo Ferrinho, Mario Roberto Dal Poz

**Affiliations:** 1 Global Health and Tropical Medicine, Instituto de Higiene e Medicina Tropical, Universidade Nova de Lisboa, Rua da Junqueira, 100, 1349-008 Lisbon, Portugal; 2 National School of Public Health, Public Health Research Centre, Comprehensive Health Research Center, NOVA University of Lisbon, Avenida Padre Cruz, 1600-560 Lisbon, Portugal; 3 School of Public, Health University of the Western Cape, South Africa, Private Bag X17, Bellville, 7535 Republic of South Africa; 4 Escola Nacional de Saúde Pública Sérgio Arouca, Fundação Osvaldo Cruz, Rua Leopoldo Bulhões, 1480 - Manguinhos, Rio de Janeiro, Brazil; 5 Instituto de Medicina Social, Universidade do Estado do Rio de Janeiro, Rua São Francisco Xavier 524 – 7º andar, Blocos D e E – Maracanã, Rio de Janeiro, RJ 20550-013 Brazil; 6 Instituto Nacional de Cancer, Ministério da Saúde, Rua Marquês de Pombal, 125, Centro, Rio de Janeiro, RJ 20230240 Brazil; 7 Health Workforce Department, World Health Organization, Av. Appia 20, 1202 Geneva, Switzerland

**Keywords:** Public health emergencies of international concern, Health and care workers, Living systematic review, Meta-analysis, SARS-CoV-2, COVID-19, SARS, Influenza, MERS, Ebola, Mental health physical health

## Abstract

**Background:**

Health and care workers (HCW) faced the double burden of the SARS-CoV-2 pandemic: as members of a society affected by a public health emergency and as HWC who experienced fear of becoming infected and of infecting others, stigma, violence, increased workloads, changes in scope of practice, among others. To understand the short and long-term impacts in terms of the COVID-19 pandemic and other public health emergencies of international concern (PHEICs) on HCW and relevant interventions to address them, we designed and conducted a living systematic review (LSR).

**Methods:**

We reviewed literature retrieved from MEDLINE—PubMed, Embase, SCOPUS, LILACS, the World Health Organization COVID-19 database, the ClinicalTrials.org and the ILO database, published from January 2000 until December 2021. We included quantitative observational studies, experimental studies, quasi-experimental, mixed methods or qualitative studies; addressing mental, physical health and well-being and quality of life. The review targeted HCW; and interventions and exposures, implemented during the COVID-19 pandemic or other PHEICs. To assess the risk of bias of included studies, we used the Johanna Briggs Institute (JBI) Critical Appraisal Tools. Data were qualitatively synthetized using meta-aggregation and meta-analysis was performed to estimate pooled prevalence of some of the outcomes.

**Results:**

The 1013 studies included in the review were mainly quantitative research, cross-sectional, with medium risk of bias/quality, addressing at least one of the following: mental health issue, violence, physical health and well-being, and quality of life. Additionally, interventions to address short- and long-term impact of PHEICs on HCW included in the review, although scarce, were mainly behavioral and individual oriented, aimed at improving mental health through the development of individual interventions. A lack of interventions addressing organizational or systemic bottlenecks was noted.

**Discussion:**

PHEICs impacted the mental and physical health of HCW with the greatest toll on mental health. The impact PHEICs are intricate and complex. The review revealed the consequences for health and care service delivery, with increased unplanned absenteeism, service disruption and occupation turnover that subvert the capacity to answer to the PHEICs, specifically challenging the resilience of health systems.

**Supplementary Information:**

The online version contains supplementary material available at 10.1186/s12960-024-00892-2.

## Background

The SARS-CoV-2 pandemic hit the world in a disruptive way, forcing stringent adaptation to a new reality, including ways of living, working, and communicating. All over the world, health and care systems were affected by the pandemic.

A strikingly high number of cases flooded health and care services with patients, many needing specialized and intensive care and demanding quick and often morally challenging decisions by health and care workers (HCW) [1, 2]. Patients with chronic conditions were “deviated” from the usual care pathways by either suspending care or reallocating them to other health units or health professionals.

HCW were inevitably involved in the turmoil of the pandemic and began to face a double burden of the pandemic [3–6]. As members of a society affected by a public health emergency, HCW faced the challenges of lockdowns, social distancing and other measures aimed at controlling the pandemic as well as its social and economic impacts, while experiencing fear of becoming infected and of infecting others [7–14], stigma, violence in the workplace and outside health facilities as they were the ones breaching the lockdown [15–17]. Many HCWs were overworked and under strenuous conditions with women more affected then men [18] and were asked to work more hours, to extend their scope of practice, to start working immediately after graduation without due guidance, subject to stigma, harassment, temporary contracts, and with no extra incentives [2].

Along with the numbers and figures of the pandemic, many media reports focused on the strain and problems that HCW were facing in terms of their mental and physical health. Some reports claimed anecdotal evidence on the absenteeism resulting from quarantines and the COVID-19 infection itself, and it was suggested that many HCWs started to miss work, suffered from burnout or/and exhaustion. Progressively, reports of some HCW leaving practice, and even the profession, emerged [19, 20].

Despite all the news, tweets and posts, and even scientific papers, there is a vacuum of knowledge on the health impacts of the COVID-19 pandemic but also of previous public health emergencies of international concern (PHEICs) [21] which is paramount to inform health and multisectoral decisions.

To systematize existing knowledge on this matter, we designed and conducted a living systematic review (LSR), “systematic review that is continually updated, incorporating relevant new evidence as it becomes available” [22] to answer to the review questions in Table 1. This is the first, comprehensive SLR to be conducted on the specific impacts of PHEIC on the health of HCW and its living nature can contribute to continuously monitor the evidence produced providing a “real-time” evidence base to support informed decisions.

**Table 1:** Review questions for the impacts for health and care workers of Covid-19

Review questions
1. What are the short and long-term impacts, in terms of morbidity, disability, mortality, violence against health care workers, attrition, performance and quality of life of COVID-19 pandemic and other public health emergencies of international concern (Severe Acute Respiratory Syndrome—SARS, Middle East Respiratory Syndrome SARS, Middle East Respiratory Syndrome—MERS, Ebola, Zika, Influenza A) on HCW?
2. What are the cost-effective and culturally relevant interventions to address short- and long-term morbidity, disability, mortality, violence against health care workers, attrition, performance, and quality of life of COVID-19 pandemic and other public health emergencies of international concern (SARS, MERS, Ebola, Zika, Influenza A) HCW?

In this paper, we report the baseline results of the LSR.

## Methods

### Protocol and registration

The protocol for this LSR has been registered in PROSPERO (registration PROSPERO 2022 CRD42022324006 (https://www.crd.york.ac.uk/prospero/display_record.php?RecordID=324006).

### Information sources

**Fig. 1:**
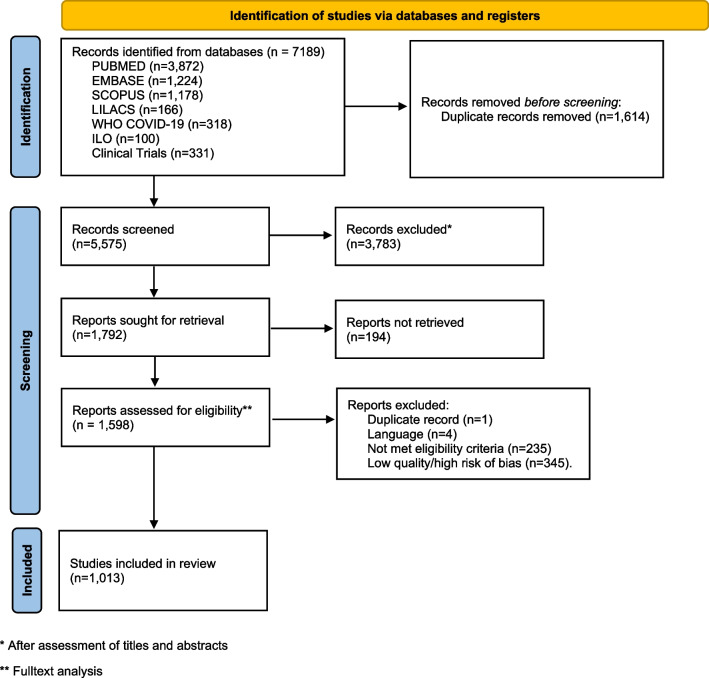
Flow diagram

MEDLINE—PubMed, Embase, Latin American and Caribbean Health Sciences Literature; SCOPUS, World Health Organization COVID-19 database; ClinicalTrials.org; and International Labour Office databases were searched in March 2022 with the last search conducted on the 4th of April 2022. The number of references detailed per database is mentioned in Fig. 1.

### Search

The search strategy for each database is detailed in Additional file 1. The search of the LSR was limited to documents published from 1st of January 2000 to 31st December 2021 in English, French, Hindi, Portuguese, Italian or Spanish.

### Selection process

The eligibility criteria for this LSR were:

- Type of study: quantitative observational studies (i.e., cohort, case–control, cross-sectional), experimental studies, quasi-experimental, mixed methods, and qualitative studies.
- Conditions studied: all studies addressing workplace hazards, physical and mental health (including stress, burnout, post-traumatic stress, suicide, and other mental health conditions), unplanned absenteeism, attrition, and intention to leave the profession, performance, violence, and quality of life were included.
- Participants/population: health and care workers as reported by the authors of the studies.
- Intervention(s), exposure(s): managerial, organizational and system strategies (i.e., environmental factors, health services, counselling, or screening) targeting health care workers implemented during the COVID-19 pandemic or other PHEICs (SARS, MERS, Ebola, Zika and Influenza A); (1) targeting health systems; (2) targeting HCW and their families; (3) targeting health services users and the public.

To address the review questions, we used PICO (Population, Intervention, Comparison, Outcome) (Table 2).

**Table 2:** PICO for impacts for health and care workers of Covid-19

Population	Health and care workers
Interventions	(1) Targeting health systems: managerial, organizational and system strategies (i.e., environmental factors, health services, counselling, or screening) targeting HCW implemented during the COVID-19 pandemic or other PHE; (2) targeting HCW and their families; (3) targeting health services users and the public
Comparison	General population, subsets of HCW, and other occupational groups
Outcomes	Workplace hazards, mental health (including stress, burnout, post-traumatic stress, suicide, and other mental health conditions), unplanned absenteeism, attrition, and intention to leave the occupation, performance, violence e quality of life

The eligibility criteria were first applied to the title and abstract/executive summary or introduction of the studies and, if met, applied again to the full text of the studies. In cases where no abstract or equivalent was available, the full text was assessed for eligibility (Additional file 2).

The titles and the abstracts of the references retrieved from the databases were imported to Rayyan and assessed blindly by four reviewers (IF, VM, WA and RL), between mid-April and May 2022. The references were distributed between reviewers with overlap of references between at least two of the reviewers. The inclusion rate (number of included references/total references assessed) was 30% for IF, 32% for VM and WA and 47% for RL. The agreement between IF and VM, IF and WA, and VM and WA was almost perfect and between WA and RL substantial [23] (Additional file 3). A total of 294 (5.3%) conflicts were found between reviewers. These were resolved by a third reviewer.

### Data collection process

The extraction of data was conducted by 5 reviewers (IF, VM, WA, RL, and KM). Each reviewer was given a list of the studies included after assessment of title and abstract. Reviewers assessed the risk of bias/quality of the study (see “Risk of bias assessment”). Studies with high risk of bias/low quality were excluded. Data were only collected for studies scored moderate to low risk of bias/moderate to high quality.

The JBI Qualitative and Qualitative Data Extraction Tools were adapted to extract data from qualitative and quantitative studies, respectively [24]. For mixed methods studies, data were retrieved using the data collection form for quantitative studies for the quantitative part and the data collection form for qualitative studies for the qualitative part of the study.

The forms for data collection were designed in REDCap [25, 26] and are available in Additional file 4.

### Risk of bias assessment

The risk of bias/quality of each included study was assessed using the JBI critical appraisal tools (CAT) (https://jbi.global/critical-appraisal-tools): Checklists for Analytical Cross Sectional Studies, Case Control Studies, Cohort Studies, Prevalence Studies (used only when the aim as stated by the authors was to estimate/compute/describe the prevalence), Qualitative Research, Quasi-Experimental Studies, and Experimental Studies.

For all checklists, there were 4 options of answer (yes/no/unclear/not applicable). To compute a risk of bias/quality score for each study, each option of answer was given the following points:

- Yes—2 points.
- No—0 points.
- Unclear—1 point
- Not applicable—missing.

For each CAT checklist the maximum score was computed by multiplying the total number of items per the maximum score in each item (2 points per YES) as detailed in Additional file 5.

The Grading of Recommendations, Assessment, Development and Evaluations (GRADE) framework [27] was used to rate the body of evidence of the outcomes (Additional file 5). For that purpose, observational studies were considered low or very low quality of evidence, experimental studies, namely randomized clinical trials were considered very high quality of evidence, with non-randomized clinical studies as well as quasi-experimental studies being classified as high quality of evidence. Qualitative studies were classified by default as very low quality of evidence. Then, for each outcome, and taking into consideration the number and type of studies, their limitations, inconsistency, indirectness, imprecision and publication bias, the quality of the evidence was determined as high, moderate, low, and very low. We present an overall qualitative assessment of the probability of publication bias (likely/very likely) for each of the outcomes considered in the meta-analysis and the graphical observation of funnel plots (Additional file 6).

### Effect measures

We used the prevalence (present/absent) of the outcome as an effect measure. The prevalence was obtained through data provided by the authors either directly whenever the prevalence was presented in the text or by computation using the number of events divided by total population.

### Synthesis methods

The data synthesis included a qualitative synthesis using a meta-aggregation approach pertaining to each research question and organized per outcome.

For each outcome we included a general summary of the context of studies, the outcomes assessed, the results and the results of the risk assessment bias. A summary of findings table is available in Additional file 7.

We conducted a maximum likelihood estimators’ random effect inverse variance meta-analysis of binary outcomes with pre-calculated effect sizes using IBM SPSS v.29 to summarize the prevalence of the outcomes. We conducted subgroup analysis per World Bank Lending Regions (East Asia and Pacific; Europe and Central Asia; Latin America and the Caribbean; Middle East and North Africa; North America; South Asia; and sub-Saharan Africa) and multi-country studies. Whenever feasible, we further stratified the analysis per studies published before 2020 and after 2020, the year of the start of the COVID-19 pandemic.

Given the high methodological heterogeneity of the studies in terms of outcome measures and of reporting (e.g., prevalence and/or mean/median values) of the outcomes as well in study design and populations, we based the meta-analysis on the following assumptions:

- only quantitative studies or quantitative parts of mixed methods studies were included.
- all data collection instruments were equal in terms of identifying those with or without the outcome of interest.
- the source populations (HCW) were considered comparable (i.e., despite the definition used in the original research) considering solely that they worked as HCW, i.e., working in health and care system, no matter the nature of the occupation, the hierarchy, or other distinctive characteristics.
- only data on prevalence (yes/no) were considered (whenever needed this was computed by the researchers based on data from the studies)—all outcomes are nominal and dichotomous (yes/no) and only studies where this information was available or could be computed were included in the meta-analysis. Studies presenting only mean or median scores were not considered in the analysis. In studies reporting several degrees of the outcome (e.g., mild, severe, etc.) abnormal categories were categorized as yes and normal as no.
- studies comparing the outcome in different periods of time (e.g., first vs. second wave of the pandemic) were excluded since we were computing prevalence of the outcome and not considering the time of the pandemic.

The combined prevalence of the outcome and the 95% confidence interval (95CI), the *I*^2^ statistics for homogeneity, the forest plot and the funnel plot are detailed in Table 3 and in Additional file 7.

**Table 3:** Effect size estimates of the outcomes (total and per region), test of homogeneity (*I*^2^) and GRADE quality of the evidence (below the outcome is the total number of studies mentioning the outcome)

Outcome		Effect size estimates									*I*^2^	GRADE quality of the evidence
		Included in meta-analysis	For subgroup analysis									
			East Asia and Pacific	Europe and Central Asia	Latin America and the Caribbean	Middle East and North Africa	North America	South Asia	Sub-Saharan Africa	Multi-country studies		
Anxiety^a^ (*N* = 518)	*n*	350	106	83	23	44	38	32	13	11	1.00	Very low—346 cross-sectional studies, 3 cohorts and 1 case–control, 57% medium quality/risk of bias, 43% high quality/low risk of bias, very serious inconsistency, very serious imprecision, unlikely publication bias, no large effect, no evidence of a dose–response gradient, all plausible confounding would suggest a spurious effect
	*P*	39%	31%	40%	44%	48%	37%	45%	49%	32%		
	95CI	[37; 41]	[27; 34]	[36; 45]	[36; 52]	[41; 54]	[31; 43]	[38; 52]	[35; 62]	[17; 47]		
Depression (*N* = 503)	*Before 2020*											
	*n*	3	–	–	–	–	–	–	–	–	0.98	Very low—3 cross-sectional studies, 1 medium quality/risk of bias, 2 high quality/low risk of bias, very serious inconsistency, very serious imprecision, unlikely publication bias, no large effect, no evidence of a dose–response gradient, all plausible confounding would suggest a spurious effect
	*P*	21%	–	–	–	–	–	–	–	–		
	95CI	[5; 37]	–	–	–	–	–	–	–	–		
	*From 2020 onward*s											
	*n*	370	108	90	22	48	45	34	13	10	1.00	Very low—367 cross-sectional studies, 2 cohorts and 1 case–control, 55% medium quality/risk of bias, 45% high quality/low risk of bias, very serious inconsistency, very serious imprecision, unlikely publication bias, no large effect, no evidence of a dose–response gradient, all plausible confounding would suggest a spurious effect
	*P*	35%	32%	35%	34%	44%	31%	39%	41%	20%		
	95CI	[33; 37]	[29; 35]	[31; 39]	[26; 42]	[39; 50]	[26; 36]	[32; 45]	[31; 51]	[11; 29]		
Stress^b^ (*N* = 486)	*Before 2020*											
	*n*	2	–	–	–	–	–	–	–	–	1.00	Very low—2 cross-sectional studies, 1 medium quality/risk of bias, 1 high quality/low risk of bias, very serious inconsistency, very serious imprecision, unlikely publication bias, no large effect, no evidence of a dose–response gradient, all plausible confounding would suggest a spurious effect
	*P*	40%	–	–	–	–	–	–	–	–		
	95CI	[0; 79]	–	–	–	–	–	–	–	–		
	*From 2020 onwards*											
	*n*	158	41	38	9	25	11	23	7	4	1.00	Very low—157 cross-sectional studies and 1 case–control, 63% medium quality/risk of bias, 37% high quality/low risk of bias, very serious inconsistency, very serious imprecision, unlikely publication bias, no large effect, no evidence of a dose–response gradient, all plausible confounding would suggest a spurious effect
	*P*	44%	39%	46%	34%	50%	38%	48%	51%	35%		
	95CI	[40; 48]	[32; 46]	[38; 54]	[22; 47]	[41; 60]	[25; 50]	[37; 59]	[39; 63]	[3; 67]		
Burnout^a^ (*N* = 235)	*n*	94	24	23	8	12	20	1	4	2	1.00	Very low—90 cross-sectional studies and 4 cohort, 52% medium quality/risk of bias, 48% high quality/low risk of bias, very serious inconsistency, very serious imprecision, unlikely publication bias, no large effect, no evidence of a dose–response gradient, all plausible confounding would suggest a spurious effect
	*P*	46%	52%	46%	34%	55%	42%	–	41%	46%		
	95CI	[42; 51]	[43; 61]	[38; 55]	[18; 49]	[43; 66]	[32; 51]	–	[16; 67]	[18; 75]		
PTSD^a^ (*N* = 84)	*n*	53	12	23	4	2	8	1	2	1	1.00	Very low—52 cross-sectional, 1 cohort, 89% medium quality/risk of bias, 43% high quality/low risk of bias, very serious inconsistency, very serious imprecision, unlikely publication bias, no large effect, no evidence of a dose–response gradient, all plausible confounding would suggest a spurious effect
	*P*	26%	26%	25%	22%	44%	24%	–	56%	–		
	95CI	[22; 31]	[16; 36]	[18; 31]	[6; 38]	[16; 72]	[15; 32]	–	[52; 59]	–		
Suicidal ideation^a^ (*N* = 18)	*n*	15	4	7	1	–	3	–	–	–	0.98	Very low—12 cross-sectional, 60% medium quality/risk of bias, 40% high quality/low risk of bias, very serious inconsistency, very serious imprecision, unlikely publication bias, no large effect, no evidence of a dose–response gradient, all plausible confounding would suggest a spurious effect
	*P*	7%	9%	5%	–	–	8%	–	–	–		
	95CI	[5; 8]	[6; 12]	[3; 7]	–	–	[8; 9]	–	–	–		
Headaches^a,c^ (*N* = 12)	*n*	12	2	2	1	–	1	5	–	1	0.99	Very low—12 cross-sectional studies, 92% medium quality/risk of bias, 8% high quality/low risk of bias, very serious inconsistency, very serious imprecision, unlikely publication bias, no large effect, no evidence of a dose–response gradient, all plausible confounding would suggest a spurious effect
	*P*	53%	43%	36%	–	–	–	53%	–	–		
	95CI	[38; 67]	NA	[23; 49]	–	–	–	[39; 77]	–	–		
Sleep disorders (*N* = 90)	*n*	54	13	9	3	12	6	4	5	2	0.99	Very low—54 cross-sectional studies, 52% medium quality/risk of bias, 48% high quality/low risk of bias, very serious inconsistency, very serious imprecision, unlikely publication bias, no large effect, no evidence of a dose–response gradient, all plausible confounding would suggest a spurious effect
	*P*	36%	40%	31%	33%	38%	50%	34%	24%	32%		
	95CI	[31; 41]	[30; 50]	[21; 41]	NA	[29; 46]	[33; 67]	[17; 51]	[12; 37]	[12; 52]		
Skin-related morbidity (*N* = 19)	*n*	14	4	6	1	1	1	1	–	–	1.00	Very low—14 cross-sectional studies, 86% medium quality/risk of bias, 14% high quality/low risk of bias, very serious inconsistency, very serious imprecision, unlikely publication bias, no large effect, no evidence of a dose–response gradient, all plausible confounding would suggest a spurious effect
	*P*	51%	48%	64%	–	–	–	–	–	–		
	95CI	[39; 64]	[43; 53]	[43; 84]	–	–	–	–	–	–		
Violence (*N* = 32)	*n*	11	1	2	3	2	–	2	–	1	1.00	Very low—11 cross-sectional studies, 64% medium quality/risk of bias, 36% high quality/low risk of bias, very serious inconsistency, very serious imprecision, unlikely publication bias, no large effect, no evidence of a dose–response gradient, all plausible confounding would suggest a spurious effect
	*P*	48%	–	30%	58%	81%	–	19	–	–		
	95CI	[32; 64]	–	[28; 32]	[31; 84]	NA	–	[17; 21]	–	–		

*N* total number of studies included in the LSR for a given outcome, *n* studies included in the meta-analysis, *P* prevalence of the outcome in percentage, *95CI* 95% confidence interval for the prevalence, *NA* not available

^a^None of the included studies in the meta-analysis were published before 2020

^b^Only studies measuring stress were considered for meta-analysis

^c^Only data on the prevalence of de novo headaches are included in the meta-analysis

This LSR did not require approval by an Ethics Committee, but ethical consideration pertaining to the use of secondary data was undertaken by the review team.

## Results

A total of 1013 studies were included (Additional file 6) and 3783 were excluded after assessment of eligibility criteria in the title and abstract: 585 studies were excluded after reading the full text: 1 was a duplicate, 4 were in a language not considered in the LSR, for 194 it was not possible to access the full text or were abstracts from conferences or posters and 235 did not meet at least one for the four eligibility criteria. A total of 345 studies, presented high risk of bias/low quality and were, thus, also excluded (Fig. 1). A table of excluded studies is provided in Additional file 8.

The evidence of this LSR is derived from 1013 studies, mainly quantitative with a cross-sectional design, addressing the impacts of COVID-19 in HCW. In general, the quality of the evidence per outcome was very low, according to GRADE.

**Fig. 2:**
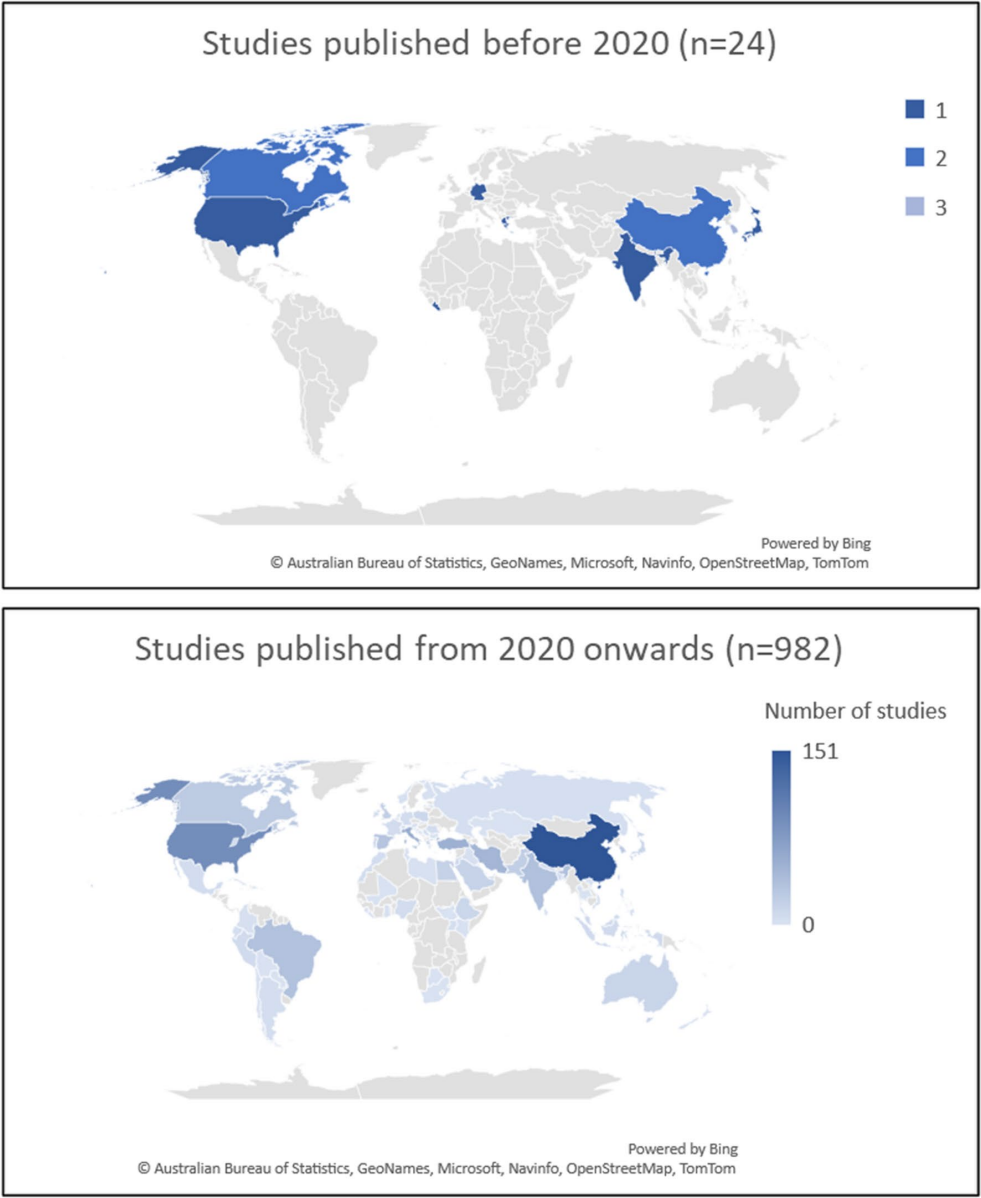
Geographical distribution of the studies included in the LSR

The majority of the studies were mostly concerned with the impact of PHEICs, especially the COVID-19 pandemic, on the health of HCW and, more specifically, on mental health. The studies covered a variety of countries and workplaces in all continents (with a bias to the Global North and with the vast majority of studies being published from 2020 onwards and focusing on the COVID-19 pandemic) (Fig. 2), as well as HCW in general or per type of occupation (with a greater focus on nurses and physicians), thus allowing to have a comprehensive glimpse of the health and care workforce. Much of the social rejection or other negative experiences that HCW experienced due to a disproportionate exposure to PHEIC and high risk of infection were often associated with mental health conditions [28] and these often resulted in somatization [29]. The conditions under which HCW were forced to work (e.g., with PPE, during extended working periods, with changes in the organization of work), and faced with insufficient or inadequate coping strategies or resilience [30–34], impacted their physical health with reports of PPE-related skin injuries and headaches or sleep disturbances. All these factors negatively impacted the performance of HCW, leading, for instance to absenteeism [35], financial problems (that backlashed on mental health) [36] and inevitably resulted in regret about choosing the profession or even intention to resign or change career [37–39].

One of the most mentioned impacts of the PHEIC was on mental health, namely stress/distress, anxiety, depression, burnout, post-traumatic stress disorder (PTSD) and suicidal ideation (thoughts), among others.

### Anxiety

Contrary to other mental health outcomes, such as burnout, suicide, depression or psychological well-being in general [40–47], anxiety in HCW did not seem to be a major concern reflected in the published literature before 2020 [44, 48], although its relevance became evident with the COVID-19 pandemic. Anxiety was frequently reported in the studies included in the LSR (*N* = 518), either as the sole outcome or measured along with depression and/or stress, among other mental health outcomes. The overall prevalence of anxiety in HCW was 39% (95CI = [37; 41]) with low variation between regions 31% (95CI = [27; 34]) in East Asia and Pacific and 49% (95CI = [35; 62]) in sub-Saharan Africa (Table 3 and Additional file 6). This prevalence was related only with the SARS-CoV-2 pandemic.

Generally, there were higher levels of anxiety in HCW when compared to the general population [38, 49–51], in healthcare providers vs non-healthcare providers [52, 53], in frontline HCW compared to non-frontliners [54–60], in those working in high incidence areas [61–63], with infected patients [52–54] and in relation to the pre-PHEIC period [64]. Anxiety was also frequently reported in female HCW [5, 51, 57, 63, 65–87].

Anxiety was frequently associated with depression and stress [88–92], sharing some of its determinants. Anxiety and depression were commonly either the result or the drivers for burnout, stress, distress and PTSD and poor well-being [31, 65, 93–97]. All tended to relate to sleep quality and sleep disorders [98–102]. Some resulted from or in somatization [103].

Anxiety emerged as an early consequence of the PHEIC that would: (i) either resolve by itself (improvement in symptoms of anxiety between the start the of the PHEIC and subsequent periods, even in HCW who had contracted the disease [104–112] but much dependent on overall incidence of the infection [113]; (ii) or evolve to more serious presentations of mental health conditions such as depression [66], PTSD or suicidal ideation [114]. Nevertheless, the levels of anxiety tended to remain high for long periods of time [105, 106] (Additional file 9).

### Depression

Depression was the second outcome most frequently addressed (*N* = 503). With a prevalence of 21% (95CI = [5; 37]) before 2020 and 35% (95CI = [33; 37]) after 2020 (Table 3 and Additional file 6), depression, as anxiety, seemed to be more prevalent in HCW compared to the overall population [115, 116], among frontliners [5, 55, 74, 77, 117–124], those caring for patients [81, 125–127], especially for COVID-19 patients, and even on HCW that had become infected [50, 67, 77, 88, 88, 98, 109, 125, 128–130]. Female gender was also mentioned to be related with depression [51, 66, 71, 74, 80, 86–88, 88, 93, 98, 122, 128, 129, 131–136].

The changes in clinical and operational practices and the level of PHEICs’ preparedness of health and care services along with adjustments in professional roles [93, 98, 137–141] were associated with depression. PHEIC seemed to exacerbate or add to existing mental conditions [67, 69, 87, 119, 131, 140, 142–145] (Table 3). High levels of anxiety and depression prevented health professionals from psychologically detaching from work [8] leading to burnout [146] and stress [147].

Health and care occupations are considered very stressful with long work hours, frequent night work, and shift duties. Hence, when compared to the with general population, HCW even in non-PHEIC situations, face high risk of stress, poor sleep patterns, fatigue and burnout [148]. Not surprisingly, as a result of the PHEICs [149–152], 40% (95CI = [0; 70%]) of HCW reported experiencing some level of stress before 2020 with the prevalence increasing after 2020 to 44% (95CI = [40; 48%]) (Table 3 and Additional file 6). Usually stress is higher in HCW than the general population [153, 154] and, despite the manifestations being more frequently psychological than physical [155, 156], few sought professional mental health support [157]. Stress resulted mainly from working conditions like the complexity of patients and concerns about transmitting the disease [155, 158], disruption of familiar and social networks, exposure to disease [159] but also from workload and levels of perceived anxiety and depression [160] (Additional file 9). Several studies mentioned stress to be related with female gender in HCW [67, 70, 74, 80, 87, 111, 122, 123, 131, 133, 139, 160–166].

### Burnout

Job stress, staff and resource adequacy, interprofessional relationships in healthcare practice, fear of infection and anxiety related to work during the PHEIC largely contributed to emotional and mental exhaustion of HCW often leading to burnout [167, 168]. Burnout addressed in 235 of the included studies, had a prevalence of 46% (95CI = [42; 51]) and was mainly reported after 2020 with studies conducted on East Asia and Pacific Region showing higher prevalence than in other regions (Table 3 and Additional file 6). Burnout seems to be higher during PHEICs in relation to the pre-PHEIC period [169–172], evolved over time [173] and manifested through physical (chronic fatigue, extreme exhaustion, reduced energy, and sleep disturbances), emotional (frustration, irritability, anger and fear), cognitive (mental fatigue, difficulty in decisions) and behavioral (negativism, emotional outbursts, cynicism, rudeness) symptoms [169]. Its negative impact is far reaching and includes not only harm to the burnt out HCW, but also to patients, co-workers, family members, close friends, and healthcare organizations [174]. Similarly, burnout was frequently more reported in female HCW [63, 66, 77, 135, 167, 175–185].

Burnout and other mental health conditions, more frequent in women, together with feelings of dehumanization of self and/or of others can potentiate PTSD [186–188]. A total of 84 studies addressed PTSD. The pooled prevalence of PTSD, after 2020, was 26% (95CI = [22; 31]) (Table 3 and Additional file 6). The studies point to an excess of PTSD in HCW when compared to general population [50], before the declaration of PHEIC [108] and in frontliners [66, 215]. Previous mental health conditions, especially stress, work in frontline services, high workload and access and use of PPE were the main determinants referred by the literature. PTSD seemed to be linked to suicidal thoughts [186, 189] (Additional file 9).

A total of 18 studies addressed suicidal ideation in HCW. The pooled prevalence of suicidal ideation and/or attempt was 7% (95CI = [5; 8]). Thoughts of suicide or self-harm were frequently related with depression and other previous mental health conditions [156, 157]. Young, male, living alone HCW were the most frequently affected (Additional file 9).

Sleep disorders, headaches and migraines, skin-related morbidity and other health issues. In this LSR we also found sleep disorders (pooled prevalence of 36%; 95CI = [31; 41]), headaches/migraines (pooled prevalence of de novo headaches 53%; 95CI = [38; 67]) and skin-related morbidity (pooled prevalence of 51%; 95CI = [39; 64]) to be frequently reported physical health impacts of PHEIC (Table 3 and Additional file 6). Other less frequent PHEICs-related morbidity studied in HCW included musculoskeletal disorders, erectile dysfunction, eye strain, weight gain, constipation and risk of infection [159, 190–194].

PHEICS often require HCW to use sophisticated PPE (e.g., gloves, respirators, eye protection, face shields masks, full body suites) more frequently and for prolonged periods of time, which seemed to be associated with dermatitis, pressure injuries, excessive heating and sweating, headaches and/or migraines, breathing difficulties, itching, cracking, burning, flaking, peeling and/or rash [195–203], although complaints varied greatly with the equipment used. Actually, the use of PPE tended to induce de novo headaches and migraines or worsen pre-existing ones, a couple of hours after the end of the shift [204, 205].

### Workplace violence

In the context of a PHEICS, workplace violence emerged also as a relevant impact, with a pooled prevalence of 48% (95CI = [32; 64]) among HCW, from 2020 onwards (Table 3 and Additional file 6). Known in their communities as HCW, during PHEICs these professionals cannot escape scrutiny and face stigma and violent episodes, even if they are working remotely [206]. HCW continue to move freely even in curfews and lockdowns and have often to quarantine even if not infected, which places them at risk of extortion and other violent acts [207]. The determinants of violence in PHEICs do not seem to differ from those identified in non-PHEIC periods and include, among others, unsupportive environment and lack of guidelines or appropriate measures to implement necessary health protocols (399 402) (Additional file 9). Also in the case of PHEICs, violence [208, 209] and stigma [120, 140, 142] seem to contribute to poor mental health.

HCW and the impact that their work has on their health has been studied in the past, in particular throughout the developed world where markedly high rates of sickness absence, sickness presenteeism, burnout, and distress compared to what has been described for other sectors [210, 211].

### Unplanned absenteeism

PHEICs are inevitably linked to unplanned absenteeism. This results from HCW becoming infected, bearing the burden of working in services directly linked to the management of the PHEIC, increased physical and mental morbidity, having to assist relatives or due to non-pharmacological measures such as quarantine [212]. Other recognized determinants of absenteeism in HCW include organizational aspects, inadequate working conditions, long hours, task overload, interpersonal conflicts, low autonomy and remuneration, associated with psychological, cognitive and physical professional overload [213], all aggravated during a PHEIC. Sometimes, HCW might opt to work even if not feeling well, a practice known as sickness presenteeism which as deleterious effects such as increased risk of burnout or loss of productivity [211, 214].

### Attrition

Leaving or intention to leave the occupation emerged as a relevant impact of PHEICs [215, 216]. Contrary to unplanned absenteeism, it is more definitive and represents a peril for the sustainability of provision of care during the PHEIC and afterwards. Sometimes, it is preceded by department or institution turnover [217–219]. Besides, working conditions like understaffing or increased work hours [20, 37, 220], mental health issues seemed to be the most relevant determinants [37, 38, 221] (Additional file 9).

Among the included studies, we only identified 9 interventions that included behavioral and organizational approaches directed at individual HCW. The timing and relative novelty and somewhat rapid resolution of most of the PHEICs might explain the lack of studies to address interventions to tackle their effects. In this LSR, all but one, which addressed workplace violence, aimed at the impacts of PHEICs on mental health. Behavioral interventions were based on therapies to increase HCW capacity to deal with stressors, building resilience and acquire and developing coping strategies [222–227]. Organizational interventions were designed to strengthen the health and care service capacity to address the challenges imposed by the PHEIC [228, 229]. Nevertheless, the overall evidence on this matter was very weak (Additional file 10).

## Discussion

Included studies were assessed in terms of risk of bias/quality of the study with those with low quality being excluded from the evidence synthesis. If on one hand this decision might have left out studies focusing on, for instance, less frequent outcomes, or those for which there is no standardized instruments for their measure, on the other hand was essential to base conclusions on less biased results.

Throughout the analysis a publication bias (i.e., tendency to include study that conform to their preconceived notions or outcomes) was not detected, since studies demonstrating effects and those not demonstrating that same effects were published and included in the analysis. Nevertheless, the large number of studies on mental health and more specifically on some of its outcomes need to be considered carefully, especially when considering pooled effect estimates—all presented very high heterogeneity. They might result from a publicity/media bias (i.e., tendency to publish “hot” topics) as these issues became more and more relevant during the pandemic.

HWC are not isolated islands. They integrate societies that also suffered the impacts of the pandemic, thus holding a double burden that it is not easy to measure.

Often, the measurement of the exposure to the PHEIC (i.e., direct contact with infected patients, nature of the contact, including duration, use of PPE, among others) was not considered in the studies which made it difficult to conclude if the changes observed in the outcomes resulted from a specific, more intense, work-related exposure or if it was restrained to that of the lay citizen (this was particularly evident in the case of the studies conducted during the COVID-19 pandemic).

Some gender differences have emerged, namely in terms of mental health outcomes. However, and because we did not consider this perspective since the design of the LSR, we cannot draw conclusions on the relevance of these studies in adding to existing evidence that reports important gender inequities between genders in HCW.

Access to and demand from HCW of in-service supportive interventions where not properly addressed in the literature. PHEICs are often associated with an intensification of work, with higher demand for care, ambiguous roles and unfamiliar work content but also to a halt on career development and increased industrial action, attrition and absences from work [230, 231].

During PHEICs, including the most recent pandemic, there was an evident concern with HCW health and well-being and the impact that PHEICs was having on their lives. The concern was mainly as professionals essential and paramount to guarantee front-line response to the challenges posed by the situation.

During PHEICs, HCW suffer stigma, divergent healthcare hero perception, added responsibilities, fear and uncertainty, worry about infecting others, broader social grief, professional exhaustion, work–life imbalance, leadership challenges and challenged performance to name a few, that negatively influence their well-being and quality of life [232–236]. The hero narrative may be detrimental to the mental well-being of HCW as it risks stifling the debate about their scope of practice and the ethical limits of duty [237].

PHEICs are opportunities to learn, to develop new skills, to improve inter and intradisciplinary collaboration and team work, and to gain the ability to balance work and life [228, 232, 234]. However, the final balance of this score card appears to remain negative as HCW tend to present poorer well-being and quality of life during the PHEIC when compared to other professionals and with the general population [238–240] with well-being stabilizing at a lower level [152].

The evidence on the impacts of PHEICs are still weak and based on cross-sectional studies.

We recommend the implementation of continuing monitoring of the health of HCW, besides the practice of traditional occupational health, in all health and care services, but specially in those more subject to PHEICs-related strain (e.g., emergency departments, intensive care units, pre-hospital emergency services). The monitoring should include extended physical health (to problems often ascertained in studies but sometimes forgotten in terms of occupational health services like sleep disorders), mental health (in general and specific prevalent problems like stress, burnout, anxiety, among others) and workplace violence screening and gender perspective. The continuous monitoring of the health and well-being of HCW should not be restricted to PHEIC periods as information on pre-PHEIC and post-PHEIC is as crucial as during PHEIC.

The LSR included a wide number of references, mostly published after the inception of the COVID-19 pandemic. Despite having included other PHEIC, such as SARS, MERS, or Ebola, most of the studies retrieved and included focused on the impact of the COVID-19 pandemic and, as such, had been published from 2020 onwards. Accordingly, the geographical distribution of the studies reflects the geographical distribution and intensity of the pandemic.

No matter the year of publication, most of the studies adopted a cross-sectional design which makes difficult to establish a timeline between PHEICs and the occurrence of the outcomes. Despite an overall consistency in the results of the studies, cross-sectional studies are weak in terms of strength of the evidence. Given that the majority of the studies included addressed the impacts of COVID-19, additional time and research is need to fully grasp the impacts of this pandemic that was still ongoing when searches were conducted in the databases. Several cohorts of HCW were created during the pandemic to monitor its impacts in the short, medium, and long term. Only 2 years have passed. This is not enough epidemiological time or even scientific time to study and publish the results.

The research gaps identified during the review require future research to address longitudinal data on the health and well-being of HCW no matter the period under consideration (in relation to PHEICs). We recommend the creation of a global, multi-country, multicenter cohort of HCW from whose observation good quality evidence can be derived. This cohort should include multiple contexts (e.g., high/middle/low income, rural/urban, underserved), different health and care services (e.g., hospital, primary health care, long-term) and departments (e.g., wards, emergency department, laboratories) and should focus on a broader understanding of the concept of HCW (similar to that adopted in this report). It should also foresee a sex-disaggregated data collection. PHEICs have shown not to spare anyone who is working in health and care services—from physicians and nurses to the auxiliary and maintenance personnel.

Although interventions aimed at HCW are relevant and seem to produce positive effects on individuals (e.g., mindfulness-based interventions appear to improve the well-being), helping them to cope and effectively manage the psychological and individual burden imposed by PHEICs, some literature suggests that interventions targeting the workplace at a system-level (including organizational, cultural, social, physical aspects) can also improve health and well-being of healthcare staff [210]. These interventions need to continue and to be expanded to cover more HCW. But they should also be complemented with initiatives to incorporate input from staff regarding their local needs and contexts and the involvement of management staff at all levels of the organization. As such, we recommend the implementation of system-level interventions that address the determinants of the impacts of the PHEIC on HCW. We also recommend that interventions can be a part of the follow-up of the HCW so that can provide in-time evidence to support the impacts of PHEIC.

Addressing the impacts of PHEIC on HCW, being able to minimize them and having a healthy, motivated, and resilient HCW is paramount to ensure universal health coverage even during crisis.

## Limitations of the study

Given the heterogeneity of HCW we have only considered as a proxy to exposure to PHEICs, being or not HCW. We acknowledge that the exposure and putative impacts of PHEICs largely depend on the occupation, type of service, etc. As such, the results of this LSR should be interpreted bearing this in mind. As should also the results that point gender differences as the gendered impacts of COVID-19 and other PHEICs were not considered in the definition of our search strategy. However, we recognize that there are critical gender imbalances and inequities in the health workforce that should be accounted for in future research on the topic.”

Despite the large number of studies on the impacts of PHEIC in the health and performance of HCWs, the majority adopt a cross-sectional design which makes difficult to establish a timeline between PHEICs and the occurrence of the outcomes, presenting a traditional example of reverse causality.

The measurement of the exposure to the PHEIC (i.e., direct contact with infected patients, nature of the contact, including duration, use of PPE, among others) is often not considered which makes it difficult to conclude if the changes observed in the frequency of studied outcomes resulted from a specific, more intense, work-related exposure or if it is restrained to that of the lay citizen (this was particularly evident in the case of the studies conducted during the COVID-19 pandemic).

### Supplementary Information

**Additional file 1.** Search strategy.

**Additional file 2.** Flow for assessing eligibility criteria.

**Additional file 3.** Interrater agreement.

**Additional file 4.** Data collection forms.

**Additional file 5.** Critical appraisal tools, score coding and GRADE.

**Additional file 6.** Forest plots and funnel plots for meta-analysis of the outcomes.

**Additional file 7.** Included studies.

**Additional file 8.** Excluded studies.

**Additional file 9.** Impact of PHEICs on HCW.

**Additional file 10.** Cost-effective and culturally relevant interventions to address short- and long-term impact of COVID-19 pandemic and other PHEICs on HCW.

## Data Availability

The database of the LSR is available upon request from the authors.

## Abbreviations

COVID-19: Coronavirus disease 19
HCW: Health and care workers
LSR: Living systematic review
PHEIC: Public health emergencies of international concern
PPE: Personal protective equipment
PTSD: Post-traumatic stress disorder
SARS: Severe acute respiratory syndrome
SARS-CoV-2: Severe acute respiratory syndrome coronavirus 2
MERS: Middle East respiratory syndrome
WHO: World Health Organization

## References

[1] United Nations, editor. International standard industrial classification of all economic activities (ISIC), (Statistical papers. Series M). Rev. 4. New York: United Nations; 2008. p. 291.

[2] World Health Organization. The impact of COVID-19 on health and care workers: a closer look at deaths. World Health Organization; 2021. Report No. WHO/HWF/WorkingPaper/2021.1. https://apps.who.int/iris/handle/10665/345300. Accessed 8 Aug 2022.

[3] Mehta S, Machado F, Kwizera A, Papazian L, Moss M, Azoulay É (2021). COVID-19: a heavy toll on health-care workers. Lancet Respir Med.

[4] Lancet T (2020). COVID-19: protecting health-care workers. Lancet.

[5] Lai J, Ma S, Wang Y, Cai Z, Hu J, Wei N (2020). Factors associated with mental health outcomes among health care workers exposed to coronavirus disease 2019. JAMA Netw Open.

[6] Gómez-Ochoa SA, Franco OH, Rojas LZ, Raguindin PF, Roa-Díaz ZM, Wyssmann BM (2021). COVID-19 in health-care workers: a living systematic review and meta-analysis of prevalence, risk factors, clinical characteristics, and outcomes. Am J Epidemiol.

[7] Chegini Z, Arab-Zozani M, Rajabi MR, Kakemam E (2021). Experiences of critical care nurses fighting against COVID-19: a qualitative phenomenological study. Nurs Forum.

[8] Llorente-Alonso M, García-Ael C, Topa G, Sanz-Muñoz ML, Muñoz-Alcalde I, Cortés-Abejer B (2021). Can psychological empowerment prevent emotional disorders in presence of fear of COVID-19 in health workers? A cross-sectional validation study. J Clin Med.

[9] Camacho KG, Gomes Junior S, Reis AT, Junqueira-Marinho MF, França LCM, Abramov DM (2022). Repercussions of the COVID-19 pandemic on health professionals in the state of Rio de Janeiro/Brazil. PLoS ONE.

[10] Seo YE, Kim HC, Yoo SY, Lee KU, Lee HW, Lee SH (2020). Factors associated with burnout among healthcare workers during an outbreak of MERS. Psychiatry Investig.

[11] Prasad K, McLoughlin C, Stillman M, Poplau S, Goelz E, Taylor S (2021). Prevalence and correlates of stress and burnout among US healthcare workers during the COVID-19 pandemic: a national cross-sectional survey study. EClinicalMedicine.

[12] Ariyo JO, Akinnawo EO, Akpunne BC, Kumuyi DO, Onisile DF (2022). An investigation of associations and incidence of anxiety, depression, perceived vulnerability to diseases, and fear of COVID-19 among Nigerian health care workers. Arch Pediatr Infect Dis.

[13] Patel AV, Kandre DD, Mehta P, Prajapati A, Patel B, Prajapati S (2021). Multi-centric study of psychological disturbances among health care workers in tertiary care centers of Western India during the COVID-19 pandemic. Neuropsychiatr Neuropsychol.

[14] Cho M, Kim O, Pang Y, Kim B, Jeong H, Lee J (2021). Factors affecting frontline Korean nurses’ mental health during the COVID-19 pandemic. Int Nurs Rev.

[15] Chen CS, Wu HY, Yang P, Yen CF (2005). Psychological distress of nurses in Taiwan who worked during the outbreak of SARS. Psychiatr Serv.

[16] Adom D, Mensah JA, Osei M (2021). The psychological distress and mental health disorders from COVID-19 stigmatization in Ghana. Soc Sci Humanit Open.

[17] Adhikari SP, Rawal N, Shrestha DB, Budhathoki P, Banmala S, Awal S (2021). Prevalence of anxiety, depression, and perceived stigma in healthcare workers in Nepal during later phase of first wave of COVID-19 pandemic: a web-based cross-sectional survey. Cureus.

[18] Morgan R, Tan HL, Oveisi N, Memmott C, Korzuchowski A, Hawkins K (2022). Women healthcare workers’ experiences during COVID-19 and other crises: a scoping review. Int J Nurs Stud Adv.

[19] Marković I, Nikolovski S, Milojević S, Živković D, Knežević S, Mitrović A (2020). Public trust and media influence on anxiety and depression levels among skilled workers during the COVID-19 outbreak in Serbia. Vojnosanit Pregl.

[20] Lasater KB, Aiken LH, Sloane DM, French R, Martin B, Reneau K (2021). Chronic hospital nurse understaffing meets COVID-19: an observational study. BMJ Qual Saf.

[21] Emergencies: International health regulations and emergency committees. 2021. https://web.archive.org/web/20210815072835/https://www.who.int/news-room/q-a-detail/emergencies-international-health-regulations-and-emergency-committees. Accessed 6 Aug 2022

[22] Cochrane community. Living systematic reviews. Cochrane Community. 2022. https://community.cochrane.org/review-production/production-resources/living-systematic-reviews. Accessed 2 June 2021.

[23] Viera AJ, Garrett JM (2005). Understanding interobserver agreement: the Kappa statistic. Fam Med.

[24] Aromataris E, Munn Z, editors. JBI manual for evidence synthesis—JBI Global Wiki. JBI; 2020. https://synthesismanual.jbi.global. Accessed 9 Mar 2022.

[25] Harris PA, Taylor R, Thielke R, Payne J, Gonzalez N, Conde JG (2009). Research electronic data capture (REDCap)—a metadata-driven methodology and workflow process for providing translational research informatics support. J Biomed Inform.

[26] Harris PA, Taylor R, Minor BL, Elliott V, Fernandez M, O’Neal L (2019). The REDCap consortium: building an international community of software platform partners. J Biomed Inform.

[27] Guyatt G, Oxman AD, Akl EA, Kunz R, Vist G, Brozek J (2011). GRADE guidelines: 1. Introduction—GRADE evidence profiles and summary of findings tables. J Clin Epidemiol.

[28] Park C, Hwang JM, Jo S, Bae SJ, Sakong J (2020). COVID-19 outbreak and its association with healthcare workers’ emotional stress: a cross-sectional study. J Korean Med Sci.

[29] Landa-Blanco M, Mejía CJ, Landa-Blanco AL, Martínez-Martínez CA, Vásquez D, Vásquez G (2021). Coronavirus awareness, confinement stress, and mental health: evidence from Honduras, Chile, Costa Rica, Mexico and Spain. Soc Sci Med.

[30] Khalaf OO, Khalil MA, Abdelmaksoud R (2020). Coping with depression and anxiety in Egyptian physicians during COVID-19 pandemic. Middle East Curr Psychiatry.

[31] Chen J, Liu X, Wang D, Jin Y, He M, Ma Y (2021). Risk factors for depression and anxiety in healthcare workers deployed during the COVID-19 outbreak in China. Soc Psychiatry Psychiatr Epidemiol.

[32] Lin J, Ren YH, Gan HJ, Chen Y, Huang YF, You XM (2020). Factors associated with resilience among non-local medical workers sent to Wuhan, China during the COVID-19 outbreak. BMC Psychiatry.

[33] Ciuluvica (Neagu) C, Gualdi G, Dal Canton M, Fantini F, Paradisi A, Sbano P (2021). Mental health consequences of the COVID-19 pandemic long-term exposure in Italian dermatologists. Int J Environ Res Public Health.

[34] Miguel-Puga JA, Cooper-Bribiesca D, Avelar-Garnica FJ, Sanchez-Hurtado LA, Colin-Martínez T, Espinosa-Poblano E (2021). Burnout, depersonalization, and anxiety contribute to post-traumatic stress in frontline health workers at COVID-19 patient care, a follow-up study. Brain Behav.

[35] Olivares-Tirado P, Zanga-Pizarro R (2022). Impact of COVID-19 pandemic outbreak on mental health of the hospital front-line healthcare workers in Chile: a difference-in-differences approach. J Public Health Oxf.

[36] Ali M, Uddin Z, Ahsan NF, Haque MZ, Bairagee M, Khan SA (2021). Prevalence of mental health symptoms and its effect on insomnia among healthcare workers who attended hospitals during COVID-19 pandemic: a survey in Dhaka city. Heliyon.

[37] Ohue T, Togo E, Ohue Y, Mitoku K (2021). Mental health of nurses involved with COVID-19 patients in Japan, intention to resign, and influencing factors. Medicine.

[38] Norkiene I, Jovarauskaite L, Kvedaraite M, Uppal E, Phull MK, Chander H (2021). “Should I Stay, or Should I Go?” Psychological distress predicts career change ideation among intensive care staff in Lithuania and the UK amid COVID-19 pandemic. Int J Environ Res Public Health.

[39] Khan N, Palepu A, Dodek P, Salmon A, Leitch H, Ruzycki S (2021). Cross-sectional survey on physician burnout during the COVID-19 pandemic in Vancouver, Canada: the role of gender, ethnicity and sexual orientation. BMJ Open.

[40] Theorell T, Hammarström A, Aronsson G, Träskman Bendz L, Grape T, Hogstedt C (2015). A systematic review including meta-analysis of work environment and depressive symptoms. BMC Public Health.

[41] Stanley IH, Hom MA, Joiner TE (2016). A systematic review of suicidal thoughts and behaviors among police officers, firefighters, EMTs, and paramedics. Clin Psychol Rev.

[42] Schneider A, Weigl M (2018). Associations between psychosocial work factors and provider mental well-being in emergency departments: a systematic review. PLoS ONE.

[43] Oskrochi Y, Maruthappu M, Henriksson M, Davies AH, Shalhoub J (2016). Beyond the body: a systematic review of the nonphysical effects of a surgical career. Surgery.

[44] O’Connor K, Muller Neff D, Pitman S (2018). Burnout in mental health professionals: a systematic review and meta-analysis of prevalence and determinants. Eur Psychiatry J Assoc Eur Psychiatr.

[45] Cocker F, Joss N (2016). Compassion fatigue among healthcare, emergency and community service workers: a systematic review. Int J Environ Res Public Health.

[46] Lever I, Dyball D, Greenberg N, Stevelink SAM (2019). Health consequences of bullying in the healthcare workplace: a systematic review. J Adv Nurs.

[47] Petrie K, Milligan-Saville J, Gayed A, Deady M, Phelps A, Dell L (2018). Prevalence of PTSD and common mental disorders amongst ambulance personnel: a systematic review and meta-analysis. Soc Psychiatry Psychiatr Epidemiol.

[48] Hall LH, Johnson J, Watt I, Tsipa A, O’Connor DB (2016). Healthcare staff wellbeing, burnout, and patient safety: a systematic review. PLoS ONE.

[49] Thakrar A, Raheem A, Chui K, Karam E, Wickramarachchi L, Chin K (2020). Trauma and orthopaedic team members’ mental health during the COVID-19 pandemic: results of a UK survey. Bone Jt Open.

[50] Lee AM, Wong JG, McAlonan GM, Cheung V, Cheung C, Sham PC (2007). Stress and psychological distress among SARS survivors 1 year after the outbreak. Can J Psychiatry.

[51] Arslan HN, Karabekiroglu A, Terzi O, Dundar C (2021). The effects of the COVID-19 outbreak on physicians’ psychological resilience levels. Postgrad Med.

[52] Zhang WR, Wang K, Yin L, Zhao WF, Xue Q, Peng M (2020). Mental health and psychosocial problems of medical health workers during the COVID-19 epidemic in China. Psychother Psychosom.

[53] Pan W, Hu J, Yi L (2020). Mental state of central sterile supply department staff during COVID-19 epidemic and CART analysis. BMC Health Serv Res.

[54] Baminiwatta A, De Silva S, Hapangama A, Basnayake K, Abayaweera C, Kulasinghe D (2021). Impact of COVID-19 on the mental health of frontline and non-frontline healthcare workers in Sri Lanka. Ceylon Med J.

[55] Heidarijamebozorgi M, Jafari H, Sadeghi R, Sheikhbardsiri H, Kargar M, Gharaghani M (2021). The prevalence of depression, anxiety, and stress among nurses during the coronavirus disease 2019: a comparison between nurses in the frontline and the second line of care delivery. Nurs Midwifery Stud.

[56] Saeed BA, Shabila NP, Aziz AJ (2021). Stress and anxiety among physicians during the COVID-19 outbreak in the Iraqi Kurdistan Region: an online survey. PLoS ONE.

[57] Pouralizadeh M, Bostani Z, Maroufizadeh S, Ghanbari A, Khoshbakht M, Alavi SA (2020). Anxiety and depression and the related factors in nurses of Guilan University of Medical Sciences hospitals during COVID-19: a web-based cross-sectional study. Int J Afr Nurs Sci.

[58] Kim SC, Quiban C, Sloan C, Montejano A (2021). Predictors of poor mental health among nurses during COVID-19 pandemic. Nurs Open.

[59] Zhou Y, Sun Z, Wang Y, Xing C, Sun L, Shang Z (2021). The prevalence of PTSS under the influence of public health emergencies in last two decades: a systematic review and meta-analysis. Clin Psychol Rev.

[60] Yang X, Chen D, Chen Y, Wang N, Lyv C, Li Y (2021). Geographical distribution and prevalence of mental disorders among healthcare workers in China:a cross-sectional country-wide survey: a cross-sectional study to assess mental disorders of healthcare workers in China. Int J Health Plan Manag.

[61] Guo WP, Min Q, Gu WW, Yu L, Xiao X, Yi WB (2021). Prevalence of mental health problems in frontline healthcare workers after the first outbreak of COVID-19 in China: a cross-sectional study. Health Qual Life Outcomes.

[62] Chen Y, Li W (2021). Influencing factors associated with mental health outcomes among dental medical staff in emergency exposed to coronavirus disease 2019: a multicenter cross-sectional study in China. Front Psychiatry.

[63] Civantos AM, Byrnes Y, Chang C, Prasad A, Chorath K, Poonia SK (2020). Mental health among otolaryngology resident and attending physicians during the COVID-19 pandemic: national study. Head Neck.

[64] Al Noaimi HM, Al Noaimi MM, Al Fayez FM, Al Mushkhes HQ, Al AW (2021). The impact of COVID-19 pandemic on mental health of health care workers of Bahrain defence force royal medical services. Bahrain Med Bull.

[65] Pazmiño Erazo EE, Alvear Velásquez MJ, Saltos Chávez IG, Pazmiño Pullas DE (2021). Factors associated with psychiatric adverse effects in healthcare personnel during the COVID-19 pandemic in Ecuador. Rev Colomb Psiquiatr.

[66] Lasalvia A, Bodini L, Amaddeo F, Porru S, Carta A, Poli R (2021). The sustained psychological impact of the COVID-19 pandemic on health care workers one year after the outbreak—a repeated cross-sectional survey in a tertiary hospital of North-East Italy. Int J Env Res Public Health.

[67] Ghaleb Y, Lami F, Al Nsour M, Rashak HA, Samy S, Khader YS (2021). Mental health impacts of COVID-19 on healthcare workers in the Eastern Mediterranean Region: a multi-country study. J Public Health Oxf.

[68] Fernández-Arana A, Olórtegui-Yzú A, Vega-Dienstmaier JM, Cuesta MJ (2022). Depression and anxiety symptoms and perceived stress in health professionals in the context of COVID-19: do adverse childhood experiences have a modulating effect?. Brain Behav.

[69] Cag Y, Erdem H, Gormez A, Ankarali H, Hargreaves S, Ferreira-Coimbra J (2021). Anxiety among front-line health-care workers supporting patients with COVID-19: a global survey. Gen Hosp Psychiatry.

[70] Saddik B, Elbarazi I, Temsah MH, Saheb Sharif-Askari F, Kheder W, Hussein A (2021). Psychological distress and anxiety levels among health care workers at the height of the COVID-19 pandemic in the United Arab Emirates. Int J Public Health.

[71] Matsumoto Y, Fujino J, Shiwaku H, Miyajima M, Doi S, Hirai N (2021). Factors affecting mental illness and social stress in hospital workers treating COVID-19: paradoxical distress during pandemic era. J Psychiatr Res.

[72] Baraka AAE, Ramadan FH, Hassan EA (2021). Predictors of critical care nurses’ stress, anxiety, and depression in response to COVID-19 pandemic. Nurs Crit Care.

[73] Mi T, Yang X, Sun S, Li X, Tam CC, Zhou Y (2021). Mental health problems of HIV healthcare providers during the COVID-19 pandemic: the interactive effects of stressors and coping. AIDS Behav.

[74] Elkholy H, Tawfik F, Ibrahim I, Salah El-Din W, Sabry M, Mohammed S (2021). Mental health of frontline healthcare workers exposed to COVID-19 in Egypt: a call for action. Int J Soc Psychiatry.

[75] Luceño-Moreno L, Talavera-Velasco B, García-Albuerne Y, Martín-García J (2020). Symptoms of posttraumatic stress, anxiety, depression, levels of resilience and burnout in Spanish health personnel during the COVID-19 pandemic. Int J Environ Res Public Health.

[76] Elawady MA, Abd-Elraouf MSED (2021). Effect of coronavirus disease 2019 pandemic on mental health among health care workers and others. Egypt J Hosp Med.

[77] Karacan FA, Yilmaz S, Kirpinar I (2021). Psychosocial adjustment of healthcare professionals during the COVID-19 pandemic: resident doctors, nurses, and caregivers need extra attention. Med J Bakirkoy.

[78] Kurt O, Deveci SE, Oguzoncul AF (2020). Levels of anxiety and depression related to covid-19 among physicians: an online cross-sectional study from Turkey. Ann Clin Anal Med.

[79] Xu L, You D, Li C, Zhang X, Yang R, Kang C (2022). Two-stage mental health survey of first-line medical staff after ending COVID-19 epidemic assistance and isolation. Eur Arch Psychiatry Clin Neurosci.

[80] Arafa A, Mohammed Z, Mahmoud O, Elshazley M, Ewis A (2021). Depressed, anxious, and stressed: what have healthcare workers on the frontlines in Egypt and Saudi Arabia experienced during the COVID-19 pandemic?. J Affect Disord.

[81] Nayak BS, Sahu PK, Ramsaroop K, Maharaj S, Mootoo W, Khan S (2021). Prevalence and factors associated with depression, anxiety and stress among healthcare workers of Trinidad and Tobago during COVID-19 pandemic: a cross-sectional study. BMJ Open.

[82] Onchonga D, Ngetich E, Makunda W, Wainaina P, Wangeshi D, Viktoria P (2021). Anxiety and depression due to 2019 SARS-CoV-2 among frontier healthcare workers in Kenya. Heliyon.

[83] Elsaie ML, Hasan MS, Zaky MS, Hussein SM, Kadah AS, Omar AM (2021). Implication of COVID-19 on the mental health of Egyptian dermatologists: a cross-sectional study. J Cosmet Dermatol.

[84] He L, Wang J, Zhang L, Wang F, Dong W, Zhao W (2021). Risk factors for anxiety and depressive symptoms in doctors during the coronavirus disease 2019 pandemic. Front Psychiatry.

[85] Kirk AHP, Chong SL, Kam KQ, Huang W, Ang LSL, Lee JH (2021). Psychosocial impact of the COVID-19 pandemic on paediatric healthcare workers. Ann Acad Med Singap.

[86] Liu S, Yang L, Zhang C, Xu Y, Cai L, Ma S (2021). Gender differences in mental health problems of healthcare workers during the coronavirus disease 2019 outbreak. J Psychiatr Res.

[87] Azizi M, Kamali M, Moosazadeh M, Aarabi M, Ghasemian R, Hasannezhad Reskati M (2021). Assessing mental health status among Iranian healthcare workers in times of the COVID-19 pandemic: a web-based cross-sectional study. Brain Behav.

[88] Motahedi S, Aghdam NF, Khajeh M, Baha R, Aliyari R, Bagheri H (2021). Anxiety and depression among healthcare workers during COVID-19 pandemic: a cross-sectional study. Heliyon.

[89] Mo Y, Deng L, Zhang L, Lang Q, Pang H, Liao C (2021). Anxiety of nurses to support Wuhan in fighting against COVID-19 epidemic and its correlation with work stress and self-efficacy. J Clin Nurs.

[90] Shao Y, Zhang W (2020). Psychological and ocular surface state of ophthalmologists and ophthalmic nurses working with patients with coronavirus disease 2019. JAMA Ophthalmol.

[91] Liu Y, Chen H, Zhang N, Wang X, Fan Q, Zhang Y (2021). Anxiety and depression symptoms of medical staff under COVID-19 epidemic in China. J Affect Disord.

[92] Mo Y, Deng L, Zhang L, Lang Q, Liao C, Wang N (2020). Work stress among Chinese nurses to support Wuhan in fighting against COVID-19 epidemic. J Nurs Manag.

[93] dos Santos KMR, Galvão MHR, Gomes SM, de Souza TA, Medeiros ADA, Barbosa IR (2021). Depressão e ansiedade em profissionais de enfermagem durante a pandemia da covid-19. Esc Anna Nery Rev Enferm.

[94] Liu Y, Wang L, Chen L, Zhang X, Bao L, Shi Y (2020). Mental health status of paediatric medical workers in China during the COVID-19 outbreak. Front Psychiatry.

[95] Franzoi IG, Granieri A, Sauta MD, Agnesone M, Gonella M, Cavallo R (2021). Anxiety, post-traumatic stress, and burnout in health professionals during the COVID-19 pandemic: comparing mental health professionals and other healthcare workers. Healthcare.

[96] Pappa S, Athanasiou N, Sakkas N, Patrinos S, Sakka E, Barmparessou Z (2021). From recession to depression? Prevalence and correlates of depression, anxiety, traumatic stress and burnout in healthcare workers during the COVID-19 pandemic in Greece: a multi-center, cross-sectional study. Int J Environ Res Public Health.

[97] Ceri V, Cicek I (2021). Psychological well-being, depression and stress during COVID-19 pandemic in Turkey: a comparative study of healthcare professionals and non-healthcare professionals. Psychol Health Med.

[98] Hasan MT, Sahadat H, Farhana S, Afifa A, Abid Hasan K, Kamrun Nahar K, et al. Prevalence of anxiety and depressive symptoms among physicians during the COVID-19 pandemic in Bangladesh: a cross-sectional study. 2020. https://medrxiv.org/cgi/content/short/2020.12.08.20245829. 10.1017/gmh.2022.30 PMC9253439 36606239

[99] Abdelghani M, Mahdy RS, El-Gohari HM (2021). Health anxiety to COVID-19 virus infection and its relationship to quality of life in a sample of health care workers in Egypt: a cross-sectional study. Arch Psychiatry Psychother.

[100] Pan X, Xiao Y, Ren D, Xu ZM, Zhang Q, Yang LY (2022). Prevalence of mental health problems and associated risk factors among military healthcare workers in specialized COVID-19 hospitals in Wuhan, China: a cross-sectional survey. Asia Pac Psychiatry.

[101] Shah ED, Pourmorteza M, Elmunzer BJ, Ballou SK, Papachristou GI, Lara LF (2021). Psychological health among gastroenterologists during the COVID-19 pandemic: a national survey. Clin Gastroenterol Hepatol.

[102] Chen X, Liu P, Lei GF, Tong L, Wang H, Zhang XQ (2021). Sleep quality and the depression-anxiety-stress state of frontline nurses who perform nucleic acid sample collection during COVID-19: a cross-sectional study. Psychol Res Behav Manag.

[103] Chew NWS, Lee GKH, Tan BYQ, Jing M, Goh Y, Ngiam NJH (2020). A multinational, multicentre study on the psychological outcomes and associated physical symptoms amongst healthcare workers during COVID-19 outbreak. Brain Behav Immun.

[104] Thng F, Rao KA, Ge L, Mao D, Neo HN, Molina JA (2021). A one-year longitudinal study: changes in depression and anxiety in frontline emergency department healthcare workers in the COVID-19 pandemic. Int J Env Res Public Health.

[105] Moallef P, Lueke NA, Gardner PJ, Patcai J (2021). Chronic PTSD and other psychological sequelae in a group of frontline healthcare workers who contracted and survived SARS. Can J Behav Sci.

[106] Vitale E (2021). Anxiety, depression and insomnia conditions in Italian nurses during the first and the second waves of the COVID-19 pandemic. J Evid Based Psychother.

[107] Castioni D, Galasso O, Rava A, Massè A, Gasparini G, Mercurio M (2021). Has the COVID-19 pandemic changed the daily practices and psychological state of orthopaedic residents?. Clin Orthop Relat Res.

[108] Havaei F, Smith P, Oudyk J, Potter GG (2021). The impact of the COVID-19 pandemic on mental health of nurses in British Columbia, Canada using trends analysis across three time points. Ann Epidemiol.

[109] Amsalem D, Lazarov A, Markowitz JC, Naiman A, Smith TE, Dixon LB (2021). Psychiatric symptoms and moral injury among US healthcare workers in the COVID-19 era. BMC Psychiatry.

[110] Zakeri MA, Rahiminezhad E, Salehi F, Ganjeh H, Dehghan M (2021). Burnout, anxiety, stress, and depression among Iranian nurses: before and during the first wave of the COVID-19 pandemic. Front Psychol.

[111] Gorini A, Fiabane E, Sommaruga M, Barbieri S, Sottotetti F, La Rovere MT (2020). Mental health and risk perception among Italian healthcare workers during the second month of the Covid-19 pandemic. Arch Psychiatr Nurs.

[112] Sánchez-Sánchez E, García-Álvarez J, García-Marín E, Gutierrez-Serrano M, Alférez MJM, Ramirez-Vargas G (2021). Impact of the COVID-19 pandemic on the mental health of nurses and auxiliary nursing care technicians—a voluntary online survey. Int J Env Res Public Health.

[113] Iqbal M, Iftikhar N, Inam SHA, Jamil H, Nisar S (2021). Comparison of depression, anxiety and stress score among clinical and non-clinical healthcare workers during the covid-19 pandemic. Pak Armed Forces Med J.

[114] Sahimi HMS, Mohd Daud TI, Chan LF, Shah SA, Rahman FHA, Nik Jaafar NR (2021). Depression and suicidal ideation in a sample of Malaysian healthcare workers: a preliminary study during the COVID-19 pandemic. Front Psychiatry.

[115] Kabasakal E, Özpulat F, Akca A, Özcebe LH (2021). Mental health status of health sector and community services employees during the COVID-19 pandemic. Int Arch Occup Environ Health.

[116] Magalhaes E, Stoner A, Palmer J, Schranze R, Grandy S, Amin S (2021). An assessment of mental health outcomes during the COVID-19 pandemic. COMMUNITY Ment Health J.

[117] Chatterjee SS, Bhattacharyya R, Bhattacharyya S, Gupta S, Das S, Banerjee BB (2020). Attitude, practice, behavior, and mental health impact of COVID-19 on doctors. Indian J Psychiatry.

[118] Elmahdy MA, Shebl EM (2021). Mental health outcomes among health care workers exposed to covid-19 pandemic, Qalyoubia governorate: cross-sectional survey. Egypt J Hosp Med.

[119] Şahin MK, Aker S, Şahin G, Karabekiroğlu A (2020). Prevalence of depression, anxiety, distress and insomnia and related factors in healthcare workers during COVID-19 pandemic in Turkey. J Community Health.

[120] Han S, Choi S, Cho SH, Lee J, Yun JY (2021). Associations between the working experiences at frontline of COVID-19 pandemic and mental health of Korean public health doctors. BMC Psychiatry.

[121] An Y, Yang Y, Wang A, Li Y, Zhang Q, Cheung T (2020). Prevalence of depression and its impact on quality of life among frontline nurses in emergency departments during the COVID-19 outbreak. J Affect Disord.

[122] Jemal K, Deriba BS, Geleta TA (2021). Psychological distress, early behavioral response, and perception toward the COVID-19 pandemic among health care workers in North Shoa Zone, Oromiya Region. Front Psychiatry.

[123] Jemal K, Deriba BS, Geleta TA, Tesema M, Awol M, Mengistu E (2021). Self-reported symptoms of depression, anxiety, and stress among healthcare workers in Ethiopia during the COVID-19 pandemic: a cross-sectional study. Neuropsychiatr Treat.

[124] Su TP, Lien TC, Yang CY, Su YL, Wang JH, Tsai SL (2007). Prevalence of psychiatric morbidity and psychological adaptation of the nurses in a structured SARS caring unit during outbreak: a prospective and periodic assessment study in Taiwan. J Psychiatr Res.

[125] Hayat K, Arshed M, Fiaz I, Afreen U, Khan FU, Khan TA (2021). Impact of COVID-19 on the mental health of healthcare workers: a cross-sectional study from Pakistan. Front Public Health.

[126] Alsairafi Z, Naser AY, Alsaleh FM, Awad A, Jalal Z (2021). Mental health status of healthcare professionals and students of health sciences faculties in Kuwait during the COVID-19 pandemic. Int J Env Res Public Health.

[127] Dziedzic B, Kobos E, Sienkiewicz Z, Idzik A (2022). Mental health of nurses during the fourth wave of the COVID-19 pandemic in Poland. Int J Environ Res Public Health.

[128] Asnakew S, Amha H, Kassew T (2021). Mental health adverse effects of COVID-19 pandemic on health care workers in North West Ethiopia: a multicenter cross-sectional study. Neuropsychiatr Treat.

[129] Bhattacharya PK, Prakash J (2021). Impact of COVID-19 pandemic on the emotional well-being of healthcare workers: a multinational cross-sectional survey. Indian J Crit Care Med.

[130] Prekazi L, Hajrullahu V, Bahtiri S, Kryeziu B, Hyseni B, Taganoviq B (2021). The impact of coping skills in post-traumatic growth of healthcare providers: when mental health is deteriorating due to COVID-19 pandemic. Front Psychol.

[131] Gilleen J, Santaolalla A, Valdearenas L, Salice C, Fusté M (2021). Impact of the COVID-19 pandemic on the mental health and well-being of UK healthcare workers. BJPsych Open.

[132] Napoli G (2022). Stress and depressive symptoms among Italian mental health nurses during the COVID-19 pandemic, a cross-sectional study. Arch Psychiatr Nurs.

[133] Zhang C, Peng D, Lv L, Zhuo K, Yu K, Shen T (2020). Individual perceived stress mediates psychological distress in medical workers during covid-19 epidemic outbreak in Wuhan. Neuropsychiatr Dis Treat.

[134] Rodolfo R, Valentina S, Francesca P, Di Giorgio L, Di Antinisca M, Alberto S, et al. Mental health outcomes among front and second line health workers associated with the COVID-19 pandemic in Italy. 2020. https://medrxiv.org/cgi/content/short/2020.04.16.20067801.

[135] Coleman JR, Abdelsattar JM, Glocker RJ (2021). COVID-19 pandemic and the lived experience of surgical residents, fellows, and early-career surgeons in the American College of Surgeons. J Am Coll Surg.

[136] Lu P, Li X, Lu L, Zhang Y (2020). The psychological states of people after Wuhan eased the lockdown. PLoS ONE.

[137] Lum A, Goh YL, Wong KS, Seah J, Teo G, Ng JQ (2021). Impact of COVID-19 on the mental health of Singaporean GPs: a cross-sectional study. BJGP Open.

[138] Mediavilla R, Fernandez-Jimenez E, Martinez-Ales G, Moreno-Kustner B, Martinez-Morata I, Jaramillo F (2021). Role of access to personal protective equipment, treatment prioritization decisions, and changes in job functions on health workers’ mental health outcomes during the initial outbreak of the COVID-19 pandemic. J Affect Disord.

[139] Youssef N, Mostafa A, Ezzat R, Yosef M, Kassas ME (2020). Mental health status of health-care professionals working in quarantine and non-quarantine Egyptian hospitals during the covid-19 pandemic. East Mediterr Health J.

[140] Khanal P, Devkota N, Dahal M, Paudel K, Joshi D (2020). Mental health impacts among health workers during COVID-19 in a low resource setting: a cross-sectional survey from Nepal. Glob Health.

[141] Wayessa ZJ, Melesse GT, Amaje Hadona E, Wako WG (2021). Prevalence of depressive symptoms due to COVID-19 and associated factors among healthcare workers in southern Ethiopia. SAGE Open Med.

[142] Hennein R, Mew EJ, Lowe SR (2021). Socio-ecological predictors of mental health outcomes among healthcare workers during the COVID-19 pandemic in the United States. PLoS ONE.

[143] Da Rosa P, Brown R, Pravecek B, Carotta C, Garcia AS, Carson P (2021). Factors associated with nurses emotional distress during the COVID-19 pandemic. Appl Nurs Res.

[144] Debski M, Abdelaziz HK, Sanderson J, Wild S, Assaf O, Wiper A (2021). Mental health outcomes among British healthcare workers-lessons from the first wave of the covid-19 pandemic. J Occup Environ Med.

[145] Zhou P, Du N, Diao D, OuYang Y, Kankanam Pathiranage HS (2021). Investigation on the influencing factors of mental health of healthcare workers for aid in Hubei during the outbreak of COVID-19. Ann Work Expo Health.

[146] Ghio L, Patti S, Piccinini G, Modafferi C, Lusetti E, Mazzella M (2021). Anxiety, depression and risk of post-traumatic stress disorder in health workers: the relationship with burnout during COVID-19 pandemic in Italy. Int J Environ Res Public Health.

[147] Al-Ghunaim TA, Johnson J, Biyani CS, O’Connor D (2021). Psychological and occupational impact of the COVID-19 pandemic on UK surgeons: a qualitative investigation. BMJ Open.

[148] Qiu D, Yu Y, Li RQ, Li YL, Xiao SY (2020). Prevalence of sleep disturbances in Chinese healthcare professionals: a systematic review and meta-analysis. Sleep Med.

[149] Aline TS, Constance A, Elise H, Bosede BA, Bouchra A, Aduragbemi BT, et al. Voices from the frontline: findings from a thematic analysis of a rapid online global survey of maternal and newborn health professionals facing the COVID-19 pandemic. 2020. https://medrxiv.org/cgi/content/short/2020.05.08.20093393. 10.1136/bmjgh-2020-002967 PMC7335688 32586891

[150] Latha SL, Priscilla T, Sudha Ty S, Saritha C, Alimchandani A, Thangaraju P (2022). Estimation of prevalence and comparing the levels of stress, anxiety, depression, and psychological impact before and after COVID-19 lockdown among front line health care workers. J Patient Exp.

[151] Tiago C, Dias-Vaz M, Barata M, Carvalho AF, Marques A (2021). Impact of the covid-19 pandemic on mental health of Portuguese anaesthesiologists from the National Health Service. Anesth Analg.

[152] Hesselink G, Straten L, Gallée L, Brants A, Holkenborg J, Barten DG (2021). Holding the frontline: a cross-sectional survey of emergency department staff well-being and psychological distress in the course of the COVID-19 outbreak. BMC Health Serv Res.

[153] Arshad MS, Hussain I, Nafees M, Majeed A, Imran I, Saeed H (2020). Assessing the impact of COVID-19 on the mental health of healthcare workers in three metropolitan cities of Pakistan. Psychol Res Behav Manag.

[154] Rus Prelog P, Matić T, Pregelj P, Sadikov A (2021). Risk of depression, anxiety, and stress during the second wave of COVID-19 in Slovenia. Front Psychiatry.

[155] Norful AA, Rosenfeld A, Schroeder K, Travers JL, Aliyu S (2021). Primary drivers and psychological manifestations of stress in frontline healthcare workforce during the initial COVID-19 outbreak in the United States. Gen Hosp Psychiatry.

[156] Gómez-Salgado J, Domínguez-Salas S, Romero-Martín M, Ortega-Moreno M, García-Iglesias JJ, Ruiz-Frutos C (2020). Sense of coherence and psychological distress among healthcare workers during the COVID-19 pandemic in Spain. Sustainability.

[157] Ma R, Oakman JM, Zhang M, Zhang X, Chen W, Buchanan NT (2021). Lessons for mental health systems from the COVID-19 front line: Chinese healthcare workers’ challenges, resources, resilience, and cultural considerations. Traumatology.

[158] Rouhbakhsh A, Badrfam R, Nejatisafa AA, Soori M, Sharafi SE, Etesam F (2022). Health care professionals’ perception of stress during COVID-19 pandemic in Iran: a qualitative study. Front Psychiatry.

[159] Li Y, Wang H, Jin XR, Li X, Pender M, Song CP (2018). Experiences and challenges in the health protection of medical teams in the Chinese Ebola treatment center, Liberia: a qualitative study. Infect Poverty.

[160] Linzer M, Stillman M, Brown R, Taylor S, Nankivil N, Poplau S (2021). Preliminary report: US physician stress during the early days of the COVID-19 pandemic. Mayo Clin Proc Innov Qual Outcomes.

[161] Gonzalo RM, Ana RG, Patricia CA, Laura AL, Nathalia GT, Luis C (2021). Short-term emotional impact of COVID-19 pandemic on Spaniard health workers. J Affect Disord.

[162] Badru OA, Oloko KO, Hassan AO, Yusuf OB, Abdur-Razaq UA, Yakub S (2021). Prevalence and correlates of psychological distress amongst healthcare workers during the COVID-19 pandemic: an online survey. Afr J Psychiatr.

[163] Jang OJ, Chung YI, Lee JW, Kim HC, Seo JS (2021). Emotional distress of the COVID-19 cluster infection on health care workers working at a national hospital in Korea. J Korean Med Sci.

[164] Ayalew M, Deribe B, Abraham Y, Reta Y, Tadesse F, Defar S (2021). Prevalence and determinant factors of mental health problems among healthcare professionals during COVID-19 pandemic in southern Ethiopia: multicentre cross-sectional study. BMJ Open.

[165] Gómez-Salgado J, Ortega-Moreno M, Soriano G, Fagundo-Rivera J, Allande-Cussó R, Ruiz-Frutos C (2021). History of contact with the SARS-COV-2 virus and the sense of coherence in the development of psychological distress in the occupational health professionals in Spain. Sci Prog.

[166] Gong H, Zhang SX, Nawaser K, Afshar Jahanshahi A, Xu X, Li J (2021). The mental health of healthcare staff working during the COVID-19 crisis: their working hours as a boundary condition. J Multidiscip Healthc.

[167] Alrawashdeh HM, Al-Tammemi AB, Alzawahreh MK, Al-Tamimi A, Elkholy M, Al Sarireh F (2021). Occupational burnout and job satisfaction among physicians in times of COVID-19 crisis: a convergent parallel mixed-method study. BMC Public Health.

[168] Algunmeeyn A, El-Dahiyat F, Altakhineh MM, Azab M, Babar ZUD (2020). Understanding the factors influencing healthcare providers’ burnout during the outbreak of COVID-19 in Jordanian hospitals. J Pharm Policy Pract.

[169] Leskovic L, Erjavec K, Leskovar R, Vukovič G (2020). Burnout and job satisfaction of healthcare workers in Slovenian nursing homes in rural areas during the COVID-19 pandemic. Ann Agric Environ Med.

[170] Kok N, van Gurp J, Teerenstra S, van der Hoeven H, Fuchs M, Hoedemaekers C (2021). Coronavirus disease 2019 immediately increases burnout symptoms in ICU professionals: a longitudinal cohort study. Crit Care Med.

[171] Seda-Gombau G, Montero-Alía JJ, Moreno-Gabriel E, Torán-Monserrat P (2021). Impact of the COVID-19 pandemic on burnout in primary care physicians in Catalonia. Int J Env Res Public Health.

[172] Serafin L, Kusiak A, Czarkowska-Pączek B (2022). The COVID-19 pandemic increased burnout and bullying among newly graduated nurses but did not impact the relationship between burnout and bullying and self-labelled subjective feeling of being bullied: a cross-sectional, comparative study. Int J Env Res Public Health.

[173] de Wit K, Mercuri M, Wallner C, Clayton N, Archambault P, Ritchie K (2020). Canadian emergency physician psychological distress and burnout during the first 10 weeks of COVID-19: a mixed-methods study. J Am Coll Emerg Physicians Open.

[174] Rothenberger DA (2017). Physician burnout and well-being: a systematic review and framework for action. Colon Rectum.

[175] Gramaglia C, Marangon D, Azzolina D, Guerriero C, Lorenzini L, Probo M (2021). The mental health impact of 2019-nCOVID on healthcare workers from North-Eastern Piedmont, Italy. Focus on burnout. Front Public Health.

[176] Di Giuseppe M, Nepa G, Prout TA, Albertini F, Marcelli S, Orrù G (2021). Stress, burnout, and resilience among healthcare workers during the COVID-19 emergency: the role of defense mechanisms. Int J Environ Res Public Health.

[177] Chen R, Sun C, Chen JJ, Jen HJ, Kang XL, Kao CC (2021). a large-scale survey on trauma, burnout, and posttraumatic growth among nurses during the COVID-19 pandemic. Int J Ment Health Nurs.

[178] Vitale E, Lupo R, Calabrò A, Cornacchia M, Conte L, Marchisio D (2021). Mapping potential risk factors in developing burnout syndrome between physicians and registered nurses suffering from an aggression in Italian emergency departments. J Psychopathol.

[179] Vitale E, Casolaro S (2021). Anxiety, burnout and depression levels according to sex and years of work experience in Italian nurses engaged in the care of covid-19 patients. J Evid Based Psychother.

[180] Khasne RW, Dhakulkar BS, Mahajan HC, Kulkarni AP (2020). Burnout among healthcare workers during COVID-19 pandemic in India: results of a questionnaire-based survey. Indian J Crit Care Med.

[181] Banerjee S, Lim KHJ, Murali K, Kamposioras K, Punie K, Oing C (2021). The impact of COVID-19 on oncology professionals: results of the ESMO Resilience Task Force survey collaboration. ESMO Open.

[182] Brera AS, Arrigoni C, Dellafiore F, Odone A, Magon A, Nania T (2021). Burnout syndrome and its determinants among healthcare workers during the first wave of the Covid-19 outbreak in Italy: a cross-sectional study to identify sex-related differences. Med Lav.

[183] Lange M, Joo S, Couette PA, Le Bas F, Humbert X (2022). Impact on mental health of the COVID-19 outbreak among general practitioners during the sanitary lockdown period. Ir J Med Sci.

[184] Stone KW, Kintziger KW, Jagger MA, Horney JA (2021). Public health workforce burnout in the COVID-19 response in the US. Int J Environ Res Public Health.

[185] Zhou LL, Zhang SE, Liu J, Wang HN, Liu L, Zhou JJ (2021). Demographic factors and job characteristics associated with burnout in Chinese female nurses during controlled COVID-19 period: a cross-sectional study. Front Public Health.

[186] Hagerty SL, Williams LM (2022). Moral injury, traumatic stress, and threats to core human needs in health-care workers: the COVID-19 pandemic as a dehumanizing experience. Clin Psychol Sci.

[187] Caillet A, Coste C, Sanchez R, Allaouchiche B (2020). Psychological impact of COVID-19 on ICU caregivers. Anaesth Crit Care Pain Med.

[188] Carmassi C, Pedrinelli V, Dell’Oste V, Bertelloni CA, Grossi C, Gesi C (2021). PTSD and depression in healthcare workers in the italian epicenter of the COVID-19 outbreak. Clin Pract Epidemiol Ment Health.

[189] Bruffaerts R, Voorspoels W, Jansen L, Kessler RC, Mortier P, Vilagut G (2021). Suicidality among healthcare professionals during the first COVID19 wave. J Affect Disord.

[190] Arca M, Dönmezdil S, Durmaz ED (2021). The effect of the COVID-19 pandemic on anxiety, depression, and musculoskeletal system complaints in healthcare workers. Work.

[191] Bulut EC, Ertaş K, Bulut D, Koparal MY, Çetin S (2021). The effect of COVID-19 epidemic on the sexual function of healthcare professionals. Andrologia.

[192] Sorokin MY, Kasyanov ED, Rukavishnikov GV, Makarevich OV, Neznanov NG, Morozov PV (2020). Stress and stigmatization in health-care workers during the COVID-19 pandemic. Indian J Psychiatry.

[193] Khorasanee R, Grundy T, Isted A, Breeze R (2021). The effects of COVID-19 on sickness of medical staff across departments: a single centre experience. Clin Med.

[194] Lee RLT, West S, Tang ACY, Cheng HY, Chong CYY, Chien WT (2021). A qualitative exploration of the experiences of school nurses during COVID-19 pandemic as the frontline primary health care professionals. Nurs Outlook.

[195] Ünver S, Yildirim M, Cansu Yeni Ğün S (2022). Personal protective equipment related skin changes among nurses working in pandemic intensive care unit: a qualitative study. J Tissue Viability.

[196] Nguyen C, Young FG, McElroy D, Singh A (2022). Personal protective equipment and adverse dermatological reactions among healthcare workers: survey observations from the COVID-19 pandemic. Medicine.

[197] Çağlar A, Kaçer İ, Hacımustafaoğlu M, Öztürk B, Öztürk K (2020). Symptoms associated with personal protective equipment among frontline health care professionals during the COVID-19 pandemic. Disaster Med Public Health Prep.

[198] Jha SK (2020). Physiological effect of N95 FFP and personal protective equipment in healthcare workers in covid ICU: a prospective cohort study. Indian J Crit Care Med.

[199] Christopher PM, Roren RS, Tania C, Jayadi NN, Cucunawangsih C (2020). Adverse skin reactions to personal protective equipment among health-care workers during COVID-19 pandemic: a multicenter cross-sectional study in Indonesia. Int J Dermatol Venereol.

[200] Daye M, Cihan FG, Durduran Y (2020). Evaluation of skin problems and dermatology life quality index in health care workers who use personal protection measures during COVID-19 pandemic. Dermatol Ther.

[201] Marraha F, Al Faker I, Charif F, Chahoub H, Benyamna Y, Rahmani N (2021). Skin reactions to personal protective equipment among first-line COVID-19 healthcare workers: a survey in northern Morocco. Ann Work Expo Health.

[202] Foo CC, Goon AT, Leow YH, Goh CL (2006). Adverse skin reactions to personal protective equipment against severe acute respiratory syndrome—a descriptive study in Singapore. Contact Dermat.

[203] Jiang Q, Liu Y, Wei W, Zhu D, Chen A, Liu H (2020). The prevalence, characteristics, and related factors of pressure injury in medical staff wearing personal protective equipment against COVID-19 in China: a multicentre cross-sectional survey. Int Wound J.

[204] Deshpande SH, Waghmare S, Jain H (2021). Difficulties faced by the healthcare workers wearing personal protective equipments in COVID-19 pandemic during summers of Mumbai City. J Clin Diagn Res.

[205] Ong JJY, Bharatendu C, Goh Y, Tang JZY, Sooi KWX, Tan YL (2020). Headaches associated with personal protective equipment—a cross-sectional study among frontline healthcare workers during COVID-19. Headache.

[206] Muñoz Del Carpio-Toia A, Begazo Muñoz Del Carpio L, Mayta-Tristan P, Alarcón-Yaquetto DE, Málaga G (2021). Workplace violence against physicians treating COVID-19 patients in Peru: a cross-sectional study. Jt Comm J Qual Patient Saf.

[207] Aborisade RA, Gbahabo DD (2021). Policing the lockdown: accounts of police officers’ aggression and extortion of frontline health workers in Nigeria. Polic Soc.

[208] Wang W, Lu L, Kelifa MM, Yu Y, He A, Cao N (2020). Mental health problems in Chinese healthcare workers exposed to workplace violence during the COVID-19 outbreak: a cross-sectional study using propensity score matching analysis. Risk Manag Health Policy.

[209] Park JS, Lee EH, Park NR, Choi YH (2018). Mental health of nurses working at a government-designated hospital during a MERS-CoV outbreak: a cross-sectional study. Arch Psychiatr Nurs.

[210] Brand SL, Thompson Coon J, Fleming LE, Carroll L, Bethel A, Wyatt K (2017). Whole-system approaches to improving the health and wellbeing of healthcare workers: a systematic review. PLoS ONE.

[211] Brborović H, Daka Q, Dakaj K, Brborović O (2017). Antecedents and associations of sickness presenteeism and sickness absenteeism in nurses: a systematic review. Int J Nurs Pract.

[212] Challener DW, Breeher LE, Frain J, Swift MD, Tosh PK, O’Horo J (2021). Healthcare personnel absenteeism, presenteeism, and staffing challenges during epidemics. Infect Control Hosp Epidemiol.

[213] Rezende R, Borges NMA, Frota OP (2012). Síndrome de Burnout e absenteísmo em enfermeiros no contexto hospitalar: revisão integrativa da literatura brasileira. Comun Ciênc Saúde.

[214] Li Y, Guo B, Wang Y, Lv X, Li R, Guan X (2021). Serial-multiple mediation of job burnout and fatigue in the relationship between sickness presenteeism and productivity loss in nurses: a multicenter cross-sectional study. Front Public Health.

[215] Jung H, Jung SY, Lee MH, Kim MS (2020). Assessing the presence of post-traumatic stress and turnover intention among nurses post-middle east respiratory syndrome outbreak: the importance of supervisor support. Workplace Health Saf.

[216] Sklar M, Ehrhart MG, Aarons GA (2021). COVID-related work changes, burnout, and turnover intentions in mental health providers: a moderated mediation analysis. Psychiatr Rehabil J.

[217] Al-Mansour K (2021). Stress and turnover intention among healthcare workers in Saudi Arabia during the time of COVID-19: can social support play a role?. PLoS ONE.

[218] Azoulay E, Pochard F, Reignier J, Argaud L, Bruneel F, Courbon P (2021). Symptoms of mental health disorders in critical care physicians facing the second COVID-19 wave: a cross-sectional study. Chest.

[219] Labrague LJ, de los Santos JAA (2021). Fear of COVID-19, psychological distress, work satisfaction and turnover intention among frontline nurses. J Nurs Manag.

[220] Li TM, Pien LC, Kao CC, Kubo T, Cheng WJ (2022). Effects of work conditions and organisational strategies on nurses’ mental health during the COVID-19 pandemic. J Nurs Manag.

[221] Jawad MK, Al-Reda DAARA, Armeah WA, Abdulhussein AJ (2021). Assessment of the burnout level in health care worker during covid 19. Indian J Forensic Med Toxicol.

[222] Chang YC, Hsu MC, Ouyang WC (2022). Effects of integrated workplace violence management intervention on occupational coping self-efficacy, goal commitment, attitudes, and confidence in emergency department nurses: a cluster-randomized controlled trial. Int J Env Res Public Health.

[223] Smith-MacDonald L, Lusk J, Lee-Baggley D, Bright K, Laidlaw A, Voth M (2021). Companions in the Abyss: a feasibility and acceptability study of an online therapy group for healthcare providers working during the COVID-19 pandemic. Front Psychiatry.

[224] Kostovich CT, Bormann JE, Gonzalez B, Hansbrough W, Kelly B, Collins EG (2021). Being present: examining the efficacy of an internet Mantram program on RN-delivered patient-centered care. Nurs Outlook.

[225] Klatt MD, Bawa R, Gabram O, Blake A, Steinberg B, Westrick A (2020). Embracing change: a mindful medical center meets COVID-19. Glob Adv Health Med.

[226] Lee S, Rozybakieva Z, Asimov M, Bagiyarova F, Tazhiyeva A, Ussebayeva N (2020). Coping strategy as a way to prevent emotional burnout in primary care doctors: a randomized controlled trial. Arch Balk Med Union.

[227] Dolić M, Antičević V, Dolić K, Pogorelić Z (2022). Difference in pandemic-related experiences and factors associated with sickness absence among nurses working in COVID-19 and non-COVID-19 departments. Int J Environ Res Public Health.

[228] Zaghini F, Fiorini J, Livigni L, Carrabs G, Sili A (2021). A mixed methods study of an organization’s approach to the COVID-19 health care crisis. Nurs Outlook.

[229] Chen R, Chou KR, Huang YJ, Wang TS, Liu SY, Ho LY (2006). Effects of a SARS prevention programme in Taiwan on nursing staff’s anxiety, depression and sleep quality: a longitudinal survey. Int J Nurs Stud.

[230] Jones A, Zhang S, Woodburn A, Dorrington S, Beck A, Winter H (2022). Experiences of staff working in a mental health trust during the COVID-19 pandemic and appraisal of staff support services. Int J Workplace Health Manag.

[231] Fan J, Hu K, Li X, Jiang Y, Zhou X, Gou X (2020). A qualitative study of the vocational and psychological perceptions and issues of transdisciplinary nurses during the COVID-19 outbreak. Aging.

[232] Ness MM, Saylor J, DiFusco LA, Evans K (2021). Leadership, professional quality of life and moral distress during COVID-19: a mixed-methods approach. J Nurs Manag.

[233] Gordon JM, Magbee T, Yoder LH (2021). The experiences of critical care nurses caring for patients with COVID-19 during the 2020 pandemic: a qualitative study. Appl Nurs Res.

[234] Rosenberg AR, Weaver MS, Fry A, Wiener L (2021). Exploring the impact of the coronavirus pandemic on pediatric palliative care clinician personal and professional well-being: a qualitative analysis of US survey data. J Pain Symptom Manag.

[235] Moreno-Mulet C, Sanso N, Carrero-Planells A, Lopez-Deflory C, Galiana L, Garcia-Pazo P (2021). The impact of the COVID-19 pandemic on ICU healthcare professionals: a mixed methods study. Int J Environ Res Public Health.

[236] da Costa Matos RA, Akutsu R, Zandonadi RP, Botelho RBA (2021). Quality of life prior and in the course of the COVID-19 pandemic: a nationwide cross-sectional study with Brazilian dietitians. Int J Env Res Public Health.

[237] Abdul Rahim, Hanan F, Fendt-Newlin, Meredith, Al-Harahsheh, Sanaa T, Campbell J. Our duty of care: a global call to action to protect the mental health of health and care workers. Doha: World Innovation Summit for Health; 2022. https://www.who.int/publications/m/item/wish_report. Accessed 17 May 2023.

[238] Bell C, Williman J, Beaglehole B, Stanley J, Jenkins M, Gendall P (2021). Challenges facing essential workers: a cross-sectional survey of the subjective mental health and well-being of New Zealand healthcare and “other” essential workers during the COVID-19 lockdown. BMJ Open.

[239] Xie XM, Zhao YJ, An FR, Zhang QE, Yu HY, Yuan Z (2021). Workplace violence and its association with quality of life among mental health professionals in China during the COVID-19 pandemic. J Psychiatr Res.

[240] Pacutova V, Madarasova Geckova A, Kizek P, de Winter AF, Reijneveld SA (2021). The impact of pandemic management on the quality of life of Slovak dentists. Int J Environ Res Public Health.

