# Supplementary material for: Impacts for health and care workers of Covid-19 and other public health emergencies of international concern: living systematic review, meta-analysis and policy recommendations
Source: Hum Resour Health. 2024 Jan 25;22:10. doi: 10.1186/s12960-024-00892-2 (PMC10809470; doi:10.1186/s12960-024-00892-2)
Supplement: Supplementary file 1 — Additional file 1. Search strategy. [file 12960_2024_892_MOESM1_ESM.docx]

**FUNNEL PLOTS**

Contents

[ANXIETY 1](#_Toc135239492)

[DEPRESSION (UNTIL 2020) 3](#_Toc135239493)

[DEPRESSION (2020 ONWARDS) 5](#_Toc135239494)

[STRESS UNTIL 2020 7](#_Toc135239495)

[STRESS 2020 ONWARDS 9](#_Toc135239496)

[BURNOUT 11](#_Toc135239497)

[PTSD 14](#_Toc135239498)

[SUICIDE 16](#_Toc135239499)

[HEADACHES 18](#_Toc135239500)

[SLEEP DISORDERS 20](#_Toc135239501)

[SKIN RELATED MORBIDITY 22](#_Toc135239502)

[VIOLENCE 24](#_Toc135239503)

# ANXIETY

| **Case Processing Summary** | | |
| --- | --- | --- |
|  | N | Percent |
| Included | 350 | 100,0% |
| Missing | 0 | 0,0% |
| Invalid^a^ | 0 | 0,0% |
| Dropped^b^ | 0 | 0,0% |
| Total | 350 | 100,0% |
| a. Risk difference less than -1 or greater than 1, or nonpositive variance or standard error. | | |
| b. Studies with either no success cases or no failure cases. | | |

| **Effect Size Estimates for Subgroup Analysis** | | | | | | |
| --- | --- | --- | --- | --- | --- | --- |
|  | Effect Size | Std. Error | Z | Sig. (2-tailed) | 95% Confidence Interval | |
|  |  |  |  |  | Lower | Upper |
| East Asia and Pacific | ,308 | ,0177 | 17,431 | <,001 | ,273 | ,342 |
| Europe and Central Asia | ,404 | ,0233 | 17,327 | <,001 | ,358 | ,450 |
| Latin America and the Caribbean | ,438 | ,0400 | 10,960 | <,001 | ,360 | ,517 |
| Middle East and North Africa | ,476 | ,0331 | 14,380 | <,001 | ,411 | ,541 |
| Muticountry | ,321 | ,0808 | 3,970 | <,001 | ,162 | ,479 |
| North America | ,369 | ,0290 | 12,722 | <,001 | ,312 | ,426 |
| South Asia | ,448 | ,0344 | 13,012 | <,001 | ,381 | ,516 |
| Sub-Saharan Africa | ,486 | ,0705 | 6,891 | <,001 | ,347 | ,624 |
| Overall | ,387 | ,0112 | 34,534 | <,001 | ,365 | ,409 |

# DEPRESSION (UNTIL 2020)

| **Case Processing Summary** | | |
| --- | --- | --- |
|  | N | Percent |
| Included | 3 | 100,0% |
| Missing | 0 | 0,0% |
| Invalid^a^ | 0 | 0,0% |
| Dropped^b^ | 0 | 0,0% |
| Total | 3 | 100,0% |
| a. Risk difference less than -1 or greater than 1, or nonpositive variance or standard error. | | |
| b. Studies with either no success cases or no failure cases. | | |

| **Effect Size Estimates** | | | | | | |
| --- | --- | --- | --- | --- | --- | --- |
|  | Effect Size | Std. Error | Z | Sig. (2-tailed) | 95% Confidence Interval | |
|  |  |  |  |  | Lower | Upper |
| Overall | ,211 | ,0808 | 2,614 | ,009 | ,053 | ,370 |

# DEPRESSION (2020 ONWARDS)

| **Case Processing Summary** | | |
| --- | --- | --- |
|  | N | Percent |
| Included | 370 | 100,0% |
| Missing | 0 | 0,0% |
| Invalid^a^ | 0 | 0,0% |
| Dropped^b^ | 0 | 0,0% |
| Total | 370 | 100,0% |
| a. Risk difference less than -1 or greater than 1, or nonpositive variance or standard error. | | |
| b. Studies with either no success cases or no failure cases. | | |

| **Effect Size Estimates for Subgroup Analysis** | | | | | | |
| --- | --- | --- | --- | --- | --- | --- |
|  | Effect Size | Std. Error | Z | Sig. (2-tailed) | 95% Confidence Interval | |
|  |  |  |  |  | Lower | Upper |
| East Asia and Pacific | ,320 | ,0172 | 18,616 | <,001 | ,286 | ,353 |
| Europe and Central Asia | ,350 | ,0206 | 17,029 | <,001 | ,310 | ,391 |
| Latin America and the Caribbean | ,339 | ,0403 | 8,425 | <,001 | ,260 | ,418 |
| Middle East and North Africa | ,443 | ,0268 | 16,549 | <,001 | ,390 | ,495 |
| Multicountry | ,198 | ,0438 | 4,512 | <,001 | ,112 | ,284 |
| North America | ,311 | ,0258 | 12,043 | <,001 | ,260 | ,362 |
| South Asia | ,385 | ,0317 | 12,141 | <,001 | ,323 | ,448 |
| Sub-Saharan Africa | ,410 | ,0511 | 8,031 | <,001 | ,310 | ,511 |
| Overall | ,349 | ,0099 | 35,350 | <,001 | ,330 | ,369 |

# STRESS UNTIL 2020

| **Case Processing Summary** | | |
| --- | --- | --- |
|  | N | Percent |
| Included | 2 | 100,0% |
| Missing | 0 | 0,0% |
| Invalid^a^ | 0 | 0,0% |
| Dropped^b^ | 0 | 0,0% |
| Total | 2 | 100,0% |
| a. Risk difference less than -1 or greater than 1, or nonpositive variance or standard error. | | |
| b. Studies with either no success cases or no failure cases. | | |

| **Effect Size Estimates** | | | | | | |
| --- | --- | --- | --- | --- | --- | --- |
|  | Effect Size | Std. Error | Z | Sig. (2-tailed) | 95% Confidence Interval | |
|  |  |  |  |  | Lower | Upper |
| Overall | ,396 | ,2018 | 1,961 | ,050 | ,000 | ,791 |

# STRESS 2020 ONWARDS

| **Case Processing Summary** | | |
| --- | --- | --- |
|  | N | Percent |
| Included | 158 | 100,0% |
| Missing | 0 | 0,0% |
| Invalid^a^ | 0 | 0,0% |
| Dropped^b^ | 0 | 0,0% |
| Total | 158 | 100,0% |
| a. Risk difference less than -1 or greater than 1, or nonpositive variance or standard error. | | |
| b. Studies with either no success cases or no failure cases. | | |

| **Effect Size Estimates for Subgroup Analysis** | | | | | | |
| --- | --- | --- | --- | --- | --- | --- |
|  | Effect Size | Std. Error | Z | Sig. (2-tailed) | 95% Confidence Interval | |
|  |  |  |  |  | Lower | Upper |
| East Asia and Pacific | ,391 | ,0367 | 10,649 | <,001 | ,319 | ,463 |
| Europe and Central Asia | ,457 | ,0404 | 11,319 | <,001 | ,378 | ,536 |
| Latin America and the Caribbean | ,343 | ,0645 | 5,320 | <,001 | ,217 | ,470 |
| Middle East and North Africa | ,503 | ,0488 | 10,303 | <,001 | ,407 | ,598 |
| Multicountry | ,347 | ,1639 | 2,117 | ,034 | ,026 | ,668 |
| North America | ,375 | ,0662 | 5,656 | <,001 | ,245 | ,504 |
| South Asia | ,482 | ,0553 | 8,717 | <,001 | ,374 | ,591 |
| Sub-Saharan Africa | ,507 | ,0616 | 8,245 | <,001 | ,387 | ,628 |
| Overall | ,438 | ,0197 | 22,279 | <,001 | ,400 | ,477 |

# BURNOUT

| **Case Processing Summary** | | |
| --- | --- | --- |
|  | N | Percent |
| Included | 94 | 100,0% |
| Missing | 0 | 0,0% |
| Invalid^a^ | 0 | 0,0% |
| Dropped^b^ | 0 | 0,0% |
| Total | 94 | 100,0% |
| a. Risk difference less than -1 or greater than 1, or nonpositive variance or standard error. | | |
| b. Studies with either no success cases or no failure cases. | | |

| **Effect Size Estimates for Subgroup Analysis** | | | | | | |
| --- | --- | --- | --- | --- | --- | --- |
|  | Effect Size | Std. Error | Z | Sig. (2-tailed) | 95% Confidence Interval | |
|  |  |  |  |  | Lower | Upper |
| East Asia and Pacific | ,515 | ,0470 | 10,965 | <,001 | ,423 | ,607 |
| Europe and Central Asia | ,460 | ,0443 | 10,393 | <,001 | ,373 | ,547 |
| Latin America and the Caribbean | ,336 | ,0833 | 4,035 | <,001 | ,173 | ,499 |
| Middle East and North Africa | ,545 | ,0629 | 8,654 | <,001 | ,421 | ,668 |
| Multicountry | ,461 | ,2072 | 2,226 | ,026 | ,055 | ,867 |
| North America | ,419 | ,0494 | 8,479 | <,001 | ,322 | ,515 |
| South Asia | ,486 | ,0295 | 16,506 | <,001 | ,428 | ,544 |
| Sub-Saharan Africa | ,412 | ,1498 | 2,752 | ,006 | ,119 | ,706 |
| Overall | ,464 | ,0233 | 19,937 | <,001 | ,418 | ,510 |

# PTSD

| **Case Processing Summary** | | |
| --- | --- | --- |
|  | N | Percent |
| Included | 53 | 100,0% |
| Missing | 0 | 0,0% |
| Invalid^a^ | 0 | 0,0% |
| Dropped^b^ | 0 | 0,0% |
| Total | 53 | 100,0% |
| a. Risk difference less than -1 or greater than 1, or nonpositive variance or standard error. | | |
| b. Studies with either no success cases or no failure cases. | | |

| **Effect Size Estimates for Subgroup Analysis** | | | | | | |
| --- | --- | --- | --- | --- | --- | --- |
|  | Effect Size | Std. Error | Z | Sig. (2-tailed) | 95% Confidence Interval | |
|  |  |  |  |  | Lower | Upper |
| East Asia and Pacific | ,259 | ,0494 | 5,233 | <,001 | ,162 | ,356 |
| Europe and Central Asia | ,248 | ,0314 | 7,891 | <,001 | ,186 | ,309 |
| Latin America and the Caribbean | ,219 | ,0692 | 3,166 | ,002 | ,083 | ,355 |
| Middle East and North Africa | ,444 | ,1008 | 4,402 | <,001 | ,246 | ,641 |
| Multicountry | ,133 | ,0030 | 43,957 | <,001 | ,127 | ,139 |
| North America | ,235 | ,0417 | 5,646 | <,001 | ,154 | ,317 |
| South Asia | ,286 | ,0248 | 11,536 | <,001 | ,238 | ,335 |
| Sub-saharan Africa | ,559 | ,0177 | 31,537 | <,001 | ,525 | ,594 |
| Overall | ,264 | ,0221 | 11,910 | <,001 | ,220 | ,307 |

# SUICIDE

| **Case Processing Summary** | | |
| --- | --- | --- |
|  | N | Percent |
| Included | 15 | 100,0% |
| Missing | 0 | 0,0% |
| Invalid^a^ | 0 | 0,0% |
| Dropped^b^ | 0 | 0,0% |
| Total | 15 | 100,0% |
| a. Risk difference less than -1 or greater than 1, or nonpositive variance or standard error. | | |
| b. Studies with either no success cases or no failure cases. | | |

| **Effect Size Estimates for Subgroup Analysis** | | | | | | |
| --- | --- | --- | --- | --- | --- | --- |
|  | Effect Size | Std. Error | Z | Sig. (2-tailed) | 95% Confidence Interval | |
|  |  |  |  |  | Lower | Upper |
| East Asia and Pacific | ,090 | ,0154 | 5,843 | <,001 | ,060 | ,120 |
| Europe and Central Asia | ,048 | ,0100 | 4,769 | <,001 | ,028 | ,067 |
| Latin America and the Caribbean | ,074 | ,0088 | 8,433 | <,001 | ,057 | ,091 |
| North America | ,084 | ,0016 | 51,132 | <,001 | ,080 | ,087 |
| Overall | ,067 | ,0077 | 8,767 | <,001 | ,052 | ,082 |

# HEADACHES

| **Case Processing Summary** | | |
| --- | --- | --- |
|  | N | Percent |
| Included | 12 | 100,0% |
| Missing | 0 | 0,0% |
| Invalid^a^ | 0 | 0,0% |
| Dropped^b^ | 0 | 0,0% |
| Total | 12 | 100,0% |
| a. Risk difference less than -1 or greater than 1, or nonpositive variance or standard error. | | |
| b. Studies with either no success cases or no failure cases. | | |

| **Effect Size Estimates for Subgroup Analysis** | | | | | | |
| --- | --- | --- | --- | --- | --- | --- |
|  | Effect Size | Std. Error | Z | Sig. (2-tailed) | 95% Confidence Interval | |
|  |  |  |  |  | Lower | Upper |
| East Asia and Pacific | ,429 | ,2685 | 1,599 | ,110 | -,097 | ,955 |
| Europe and Central Asia | ,360 | ,0657 | 5,479 | <,001 | ,231 | ,489 |
| Middle East and North Africa | ,770 | ,0275 | 27,989 | <,001 | ,716 | ,824 |
| Multicountry | ,738 | ,0195 | 37,917 | <,001 | ,700 | ,776 |
| North America | ,341 | ,0317 | 10,737 | <,001 | ,279 | ,403 |
| South Asia | ,579 | ,0956 | 6,055 | <,001 | ,392 | ,766 |
| Overall | ,526 | ,0729 | 7,220 | <,001 | ,384 | ,669 |

# SLEEP DISORDERS

| **Case Processing Summary** | | |
| --- | --- | --- |
|  | N | Percent |
| Included | 54 | 100,0% |
| Missing | 0 | 0,0% |
| Invalid^a^ | 0 | 0,0% |
| Dropped^b^ | 0 | 0,0% |
| Total | 54 | 100,0% |
| a. Risk difference less than -1 or greater than 1, or nonpositive variance or standard error. | | |
| b. Studies with either no success cases or no failure cases. | | |

| **Effect Size Estimates for Subgroup Analysis** | | | | | | |
| --- | --- | --- | --- | --- | --- | --- |
|  | Effect Size | Std. Error | Z | Sig. (2-tailed) | 95% Confidence Interval | |
|  |  |  |  |  | Lower | Upper |
| East Asia and Pacific | ,397 | ,0509 | 7,799 | <,001 | ,297 | ,497 |
| Europe and Central Asia | ,307 | ,0503 | 6,105 | <,001 | ,209 | ,406 |
| Latin America and the Caribbean | ,329 | ,1706 | 1,931 | ,054 | -,005 | ,664 |
| Middle East and North Africa | ,373 | ,0433 | 8,603 | <,001 | ,288 | ,458 |
| Multicountry | ,320 | ,1004 | 3,188 | ,001 | ,123 | ,517 |
| North America | ,500 | ,0870 | 5,749 | <,001 | ,330 | ,671 |
| South Asia | ,337 | ,0873 | 3,860 | <,001 | ,166 | ,508 |
| Subsaharan Africa | ,244 | ,0635 | 3,835 | <,001 | ,119 | ,368 |
| Overall | ,363 | ,0258 | 14,041 | <,001 | ,312 | ,414 |

# SKIN RELATED MORBIDITY

| **Case Processing Summary** | | |
| --- | --- | --- |
|  | N | Percent |
| Included | 14 | 100,0% |
| Missing | 0 | 0,0% |
| Invalid^a^ | 0 | 0,0% |
| Dropped^b^ | 0 | 0,0% |
| Total | 14 | 100,0% |
| a. Risk difference less than -1 or greater than 1, or nonpositive variance or standard error. | | |
| b. Studies with either no success cases or no failure cases. | | |

| **Effect Size Estimates for Subgroup Analysis** | | | | | | |
| --- | --- | --- | --- | --- | --- | --- |
|  | Effect Size | Std. Error | Z | Sig. (2-tailed) | 95% Confidence Interval | |
|  |  |  |  |  | Lower | Upper |
| East Asia and Pacific | ,478 | ,0263 | 18,147 | <,001 | ,427 | ,530 |
| Europe and Central Asia | ,639 | ,1040 | 6,145 | <,001 | ,435 | ,843 |
| Latin America and the Caribbean | ,696 | ,0138 | 50,345 | <,001 | ,669 | ,723 |
| Middle East and North Africa | ,451 | ,0301 | 14,962 | <,001 | ,392 | ,510 |
| North America | ,201 | ,0061 | 33,175 | <,001 | ,189 | ,213 |
| South Asia | ,100 | ,0198 | 5,055 | <,001 | ,061 | ,139 |
| Overall | ,513 | ,0642 | 7,993 | <,001 | ,388 | ,639 |

# VIOLENCE

| **Effect Size Estimates for Subgroup Analysis** | | | | | | |
| --- | --- | --- | --- | --- | --- | --- |
|  | Effect Size | Std. Error | Z | Sig. (2-tailed) | 95% Confidence Interval | |
|  |  |  |  |  | Lower | Upper |
| East Asia and Pacific | ,171 | ,0358 | 4,788 | <,001 | ,101 | ,241 |
| Europe and Central Asia | ,302 | ,0097 | 31,185 | <,001 | ,283 | ,321 |
| Latin America and the Caribbean | ,575 | ,1122 | 5,126 | <,001 | ,355 | ,795 |
| Middle East and North Africa | ,806 | ,1062 | 7,594 | <,001 | ,598 | 1,014 |
| Multicountry | ,737 | ,0147 | 50,177 | <,001 | ,708 | ,765 |
| South Asia | ,187 | ,0036 | 51,476 | <,001 | ,180 | ,194 |
| Overall | ,478 | ,0803 | 5,953 | <,001 | ,321 | ,635 |
