# Supplementary material for: Impacts for health and care workers of Covid-19 and other public health emergencies of international concern: living systematic review, meta-analysis and policy recommendations
Source: Hum Resour Health. 2024 Jan 25;22:10. doi: 10.1186/s12960-024-00892-2 (PMC10809470; doi:10.1186/s12960-024-00892-2)
Supplement: Supplementary file 2 — Additional file 2. Flow for assessing eligibility criteria. [file 12960_2024_892_MOESM2_ESM.docx]

**Included studies**

Included studies can be access here:

<https://ihmt-my.sharepoint.com/:x:/g/personal/ifronteira_ihmt_unl_pt/EcY66wPBC2VHvwt1c_HYBiMBPjrlNO3l25YbvBM2bV4B8A?e=hI6BGs>
