# Supplementary material for: Impacts for health and care workers of Covid-19 and other public health emergencies of international concern: living systematic review, meta-analysis and policy recommendations
Source: Hum Resour Health. 2024 Jan 25;22:10. doi: 10.1186/s12960-024-00892-2 (PMC10809470; doi:10.1186/s12960-024-00892-2)
Supplement: Supplementary file 3 — Additional file 3. Interrater agreement. [file 12960_2024_892_MOESM3_ESM.docx]

## Excluded studies

Three thousand, seven hundred and eighty-three studies were excluded after assessment of eligibility criteria to the title and abstract.

Five hundred and eighty-five studies were excluded after reading the full text: 1 was a duplicate, 4 were in a language not considered in the LSR, for 193 it was not possible to access the full text or were abstracts from conferences or posters and 235 did not meet at least one for the four eligibility criteria.

A total of 345 studies, after applying the Critical Appraisal Tool, were considered to present high risk of bias / low quality and were, thus, also excluded from the LSR.

Infographic of excluded studies after full text assessment

Tables of excluded studies are available here <https://ihmt-my.sharepoint.com/:f:/g/personal/ifronteira_ihmt_unl_pt/Ep30gqsutd1OhC4u0it7mHoBnkCvGYWdaKSSGkmps2xrew?e=LufCp1>
