# Supplementary material for: Impacts for health and care workers of Covid-19 and other public health emergencies of international concern: living systematic review, meta-analysis and policy recommendations
Source: Hum Resour Health. 2024 Jan 25;22:10. doi: 10.1186/s12960-024-00892-2 (PMC10809470; doi:10.1186/s12960-024-00892-2)
Supplement: Supplementary file 4 — Additional file 4. Data collection forms. [file 12960_2024_892_MOESM4_ESM.pdf]

HEALTH IMPACTS FOR HEALTH AND CARE WORKERS OF COVID-19 AND OTHER PUBLIC HEALTH EMERGENCIES

Please complete the survey below.

Thank you!

Rayyan ID __________________________________

Reviewer and study identification

Reviewer ______ Date ______

Title

Year __________________________________

Journal

Authors

ELIGIBILITY CRITERIA (2nd check)

The study participants are or include HCW?

◯ Yes
◯ No

The study addresses the impact of COVID-19, SARS, MERS, Ebola, Zika or Influenza A?

◯ Yes
◯ No

The study addresses morbidity, disability, mortality, violence against health care workers, attrition, performance and/or quality of life or includes cost-effective and culturally relevant interventions to address these?

◯ Yes
◯ No

Is it a quantitative observational (i.e., cohort, case-control, cross-sectional, and ecological), experimental, quasi-experimental, mixed methods or qualitative study?

◯ Yes
◯ No

Included (proceed) ◯ Included (proceed)

Comments

Excluded ◯ Excluded

RISK OF BIAS

Type of study (as mentioned by the author)

◯ Quantitative (includes observational, experimental and quasi-experimental)
◯ Qualitative
◯ Mixed methods

Critical appraisal tool
(chose the critical appraisal tool more adequate to the study design. In case of a mixed methods study, select the checklist for qualitative research for the qualitative part and then the checklist for quantitative part more adequate to the design of the quantitative part).

☐ Checklist for Analytical Cross Sectional Studies
☐ Checklist for Case Control Studies
☐ Checklist for Cohort Studies
☐ Checklist for Prevalence Studies (use only when the aim as stated by the authors is specifically to estimate/compute/ describe prevalence)
☐ Checklist for Qualitative Research
☐ Checklist for Quasi-Experimental Studies
☐ Checklist for Experimental Studies
☐ Ecological study

CHECKLIST FOR ANALYTICAL CROSS SECTIONAL STUDIES

	Yes	No	Unclear	Not applicable
1. Were the criteria for inclusion in the sample clearly defined?	◯	◯	◯	◯
2. Were the study subjects and the setting described in detail?	◯	◯	◯	◯
3. Was the exposure measured in a valid and reliable way?	◯	◯	◯	◯
4. Were objective, standard criteria used for measurement of the condition?	◯	◯	◯	◯

5. Were confounding factors identified?	◯	◯	◯	◯
6. Were strategies to deal with confounding factors stated?	◯	◯	◯	◯
7. Were the outcomes measured in a valid and reliable way?	◯	◯	◯	◯
8. Was appropriate statistical analysis used?	◯	◯	◯	◯

Score

____

Quality score (confirm and check) ◯ Low quality

Quality score (confirm and check) ◯ Medium quality

Quality score (confirm and check) ◯ High quality

Comments

Overall appraisal

◯ Include
◯ Exclude
◯ Seek further information

CHECKLIST FOR CASE CONTROL STUDIES

	Yes	No	Unclear	Not applicable
1. Were the groups comparable other than the presence of disease in cases or the absence of disease in controls?	◯	◯	◯	◯
2. Were cases and controls matched appropriately?	◯	◯	◯	◯
3. Were the same criteria used for identification of cases and controls?	◯	◯	◯	◯
4. Was exposure measured in a standard, valid and reliable way?	◯	◯	◯	◯
5. Was exposure measured in the same way for cases and controls?	◯	◯	◯	◯

6. Were confounding factors identified?	◯	◯	◯	◯
7. Were strategies to deal with confounding factors stated?	◯	◯	◯	◯
8. Were outcomes assessed in a standard, valid and reliable way for cases and controls?	◯	◯	◯	◯
9. Was the exposure period of interest long enough to be meaningful?	◯	◯	◯	◯
10. Was appropriate statistical analysis used?	◯	◯	◯	◯

Quality score	__________________________
Quality score (confirm and check)	◯ Low quality
Quality score (confirm and check)	◯ Medium quality
Quality score (confirm and check)	◯ High quality
Comments	

Overall appraisal

◯ Include
◯ Exclude
◯ Seek further information

CHECKLIST FOR COHORT STUDIES

	Yes	No	Unclear	Not applicable
1. Were the two groups similar and recruited from the same population?	◯	◯	◯	◯
2. Were the exposures measured similarly to assign people to both exposed and unexposed groups?	◯	◯	◯	◯
3. Was the exposure measured in a valid and reliable way?	◯	◯	◯	◯
4. Were confounding factors identified?	◯	◯	◯	◯

5. Were strategies to deal with confounding factors stated?	◯	◯	◯	◯
6. Were the groups/participants free of the outcome at the start of the study (or at the moment of exposure)?	◯	◯	◯	◯
7. Were the outcomes measured in a valid and reliable way?	◯	◯	◯	◯
8. Was the follow up time reported and sufficient to be long enough for outcomes to occur?	◯	◯	◯	◯
9. Was follow up complete, and if not, were the reasons to loss to follow up described and explored?	◯	◯	◯	◯
10. Were strategies to address incomplete follow up utilized?	◯	◯	◯	◯
11. Was appropriate statistical analysis used?	◯	◯	◯	◯

Quality score	______________________
Quality score (confirm and check)	◯ Low quality
Quality score (confirm and check)	◯ Medium quality
Quality score (confirm and check)	◯ High quality
Comments	

Overall appraisal

◯ Include
◯ Exclude
◯ Seek further information

CHECKLIST FOR STUDIES REPORTING PREVALENCE DATA

	Yes	No	Unclear	Not applicable
1. Was the sample frame appropriate to address the target population?	◯	◯	◯	◯
2. Were study participants sampled in an appropriate way?	◯	◯	◯	◯
3. Was the sample size adequate?	◯	◯	◯	◯
4. Were the study subjects and the setting described in detail?	◯	◯	◯	◯
5. Was the data analysis conducted with sufficient coverage of the identified sample?	◯	◯	◯	◯
6. Were valid methods used for the identification of the condition?	◯	◯	◯	◯
7. Was the condition measured in a standard, reliable way for all participants?	◯	◯	◯	◯
8. Was there appropriate statistical analysis?	◯	◯	◯	◯
9. Was the response rate adequate, and if not, was the low response rate managed appropriately?	◯	◯	◯	◯

Quality score ________________________________

Quality score (confirm and check) ◯ Low quality

Quality score (confirm and check) ◯ Medium quality

Quality score (confirm and check) ◯ High quality

Comments

Overall appraisal

◯ Include
◯ Exclude
◯ Seek further information

CHECKLIST FOR QUALITATIVE RESEARCH

	Yes	No	Unclear	Not applicable
1. Is there congruity between the stated philosophical perspective and the research methodology?	◯	◯	◯	◯
2. Is there congruity between the research methodology and the research question or objectives?	◯	◯	◯	◯
3. Is there congruity between the research methodology and the methods used to collect data?	◯	◯	◯	◯
4. Is there congruity between the research methodology and the representation and analysis of data?	◯	◯	◯	◯
5. Is there congruity between the research methodology and the interpretation of results?	◯	◯	◯	◯
6. Is there a statement locating the researcher culturally or theoretically?	◯	◯	◯	◯
7. Is the influence of the researcher on the research, and vice- versa, addressed?	◯	◯	◯	◯
8. Are participants, and their voices, adequately represented?	◯	◯	◯	◯
9. Is the research ethical according to current criteria or, for recent studies, and is there evidence of ethical approval by an appropriate body?	◯	◯	◯	◯
10. Do the conclusions drawn in the research report flow from the analysis, or interpretation, of the data?	◯	◯	◯	◯

Quality score ______________________________

Quality score (confirm and check) ◯ Low quality

Quality score (confirm and check) ◯ Medium quality

Quality score (confirm and check) ◯ High quality

Comments

Overall appraisal

◯ Include
◯ Exclude
◯ Seek further information

CHECKLIST FOR QUASI-EXPERIMENTAL STUDIES

	Yes	No	Unclear	Not applicable
1. Is it clear in the study what is the 'cause' and what is the 'effect' (i.e. there is no confusion about which variable comes first)?	◯	◯	◯	◯
2. Were the participants included in any comparisons similar?	◯	◯	◯	◯
3. Were the participants included in any comparisons receiving similar treatment/care, other than the exposure or intervention of interest?	◯	◯	◯	◯
4. Was there a control group?	◯	◯	◯	◯
5. Were there multiple measurements of the outcome both pre and post the intervention/exposure?	◯	◯	◯	◯
6. Was follow up complete and if not, were differences between groups in terms of their follow up adequately described and analyzed?	◯	◯	◯	◯
7. Were the outcomes of participants included in any comparisons measured in the same way?	◯	◯	◯	◯
8. Were outcomes measured in a reliable way?	◯	◯	◯	◯
9. Was appropriate statistical analysis used?	◯	◯	◯	◯

Quality score ______________________________

Quality score (confirm and check) ◯ Low quality

Quality score (confirm and check) ◯ Medium quality

Quality score (confirm and check) ◯ High quality

Comments

Overall appraisal

◯ Include
◯ Exclude
◯ Seek further information

CHECKLIST FOR EXPERIMENTAL STUDIES

	Yes	No	Unclear	Not applicable
1. Was true randomization used for assignment of participants to treatment groups?	◯	◯	◯	◯
2. Was allocation to treatment groups concealed?	◯	◯	◯	◯
3. Were treatment groups similar at the baseline?	◯	◯	◯	◯
4. Were participants blind to treatment assignment?	◯	◯	◯	◯
5. Were those delivering treatment blind to treatment assignment?	◯	◯	◯	◯
6. Were outcomes assessors blind to treatment assignment?	◯	◯	◯	◯
7. Were treatment groups treated identically other than the intervention of interest?	◯	◯	◯	◯
8. Was follow up complete and if not, were differences between groups in terms of their follow up adequately described and analyzed?	◯	◯	◯	◯
9. Were participants analyzed in the groups to which they were randomized?	◯	◯	◯	◯
10. Were outcomes measured in the same way for treatment groups?	◯	◯	◯	◯
11. Were outcomes measured in a reliable way?	◯	◯	◯	◯

12. Was appropriate statistical analysis used? ◯ ◯ ◯ ◯

13. Was the trial design appropriate, and any deviations from the standard RCT design (individual randomization, parallel groups) accounted for in the conduct and analysis of the trial? ◯ ◯ ◯ ◯

Quality score ______________________________

Quality score (confirm and check) ◯ Low quality

Quality score (confirm and check) ◯ Medium quality

Quality score (confirm and check) ◯ High quality

Comments

Overall appraisal

◯ Include
◯ Exclude
◯ Seek further information

DATA COLLECTION

This study adresses:
(Please indicate which question(s) the study adresses)

◯ Review question 1 - What are the short and long-term impacts, in terms of morbidity, disability, mortality, violence against health care workers, attrition, performance and quality of life of COVID-19 pandemic and other public health emergencies (SARS, MERS, Ebola, Zika, Influenza A) on health care workers?
◯ Review question 2 - What are the cost-effective and culturally relevant interventions to address short- and long-term morbidity, disability, mortality, violence against health care workers, attrition, performance and quality of life of COVID-19 pandemic and other public health emergencies (SARS, MERS, Ebola, Zika, Influenza A) on health care workers?
◯ Both review questions

Outcome (indicate all that apply)

☐ skin related morbidity
☐ headaches/ migraines
☐ other morbidity: ______
☐ disability
☐ violence
☐ attrition
☐ performance
☐ quality of life
☐ workplace hazards
☐ stress / distress
☐ anxiety
☐ depression
☐ burnout
☐ other mental health problems ______
☐ unplanned absenteeism
☐ intention to leave the profession
☐ workplace violence
☐ other: ______
☐ Insomnia
☐ PTSD
☐ Well being
☐ Stigma
☐ Suicidal ideation

QUALITATIVE DATA EXTRACTION TOOL

Methodology

Phenomenon of interest

Setting (include geographical and cultural)

Participants

Data analysis

Findings

QUANTITATIVE DATA EXTRACTION

Aims of the study

Setting

Study design

Follow-up or study duration

Participants (including subject characteristics)

Intervention (if applicable)

Outcomes and outcome measurements

Method of data analysis and results (prevalence, incidence, proportion, OR, risk ratio, relative risk and 95% confidence intervals)
