# Supplementary material for: Impacts for health and care workers of Covid-19 and other public health emergencies of international concern: living systematic review, meta-analysis and policy recommendations
Source: Hum Resour Health. 2024 Jan 25;22:10. doi: 10.1186/s12960-024-00892-2 (PMC10809470; doi:10.1186/s12960-024-00892-2)
Supplement: Supplementary file 5 — Additional file 5. Critical appraisal tools, score coding and GRADE. [file 12960_2024_892_MOESM5_ESM.docx]

Cost-effective and culturally relevant interventions to address short- and long-term impact of COVID-19 pandemic and other PHEICs on HCW

| **Author (year)** | **Design** | **Intervention(s) provided by enterprise to HCW** | **Outcome – access by and demand from HCW; uptake of interventions by services where they work** | **Impact observed of the support/ interventions/ services: what works or not** |
| --- | --- | --- | --- | --- |
| **Otared et al (2021)** (400) | Iran, Hospital setting  Randomized trial  The study population comprised all healthcare workers with anxiety and depression symptoms who had approached psychotherapy clinics at Tabriz city during COVID-19.  Both treatment and control group members were chosen via a psychiatric semi-structured diagnostic interview. Twenty participants were randomly selected for online ACT group-based therapy and twenty participants were randomly assigned to a control group who did not receive any treatment and were appointed to the waiting list.  40 participants joined this study (treatment group n= 20, mean age= 33.40, SD= 4.49), (control group n= 20, mean age=31.50, SD= 5.35).  Outcomes: Beck Depression Inventory, Beck Anxiety Inventory, Quality of Life Index | **Acceptance and Commitment Therapy:** eight sessions of online group-based therapy. Each session lasted about 75 min, and a multi-protocol ACT was employed following the protocol given by Hayes, Strosahl, & Wilson (2012). | Not mentioned | Decrease in the levels of **anxiety** and of **depression**; Increased in the levels of **quality of live**  (Results of the ANCOVA for post-test scores show that the effectiveness of ACT on depression (F=39.54, p <.05, hp2= 0.52), anxiety (F= 155.07, p <0.05, hp2= 0.81), quality of life (F= 27.35, p <.05, hp2= 0.42), acceptance and act (F= 44.96, p <.05,hp2= 0.55) of health workers is significant in the treatment group) |
| **Chen et al (2006)** (192) | Taiwan, Hospital setting  Cohort study  116 subjects from the nursing staff of the largest obligatory SARS designated treatment hospital in Taiwan.  Questionnaires (Zung's self-rating anxiety scale, Zung's self-rating depression scale, Pittsburgh sleep quality index) were administered before caring for SARS patients, 2 weeks after caring for SARS patients under the prevention program, 1 month after the program began, and 1 month after the hospital returned to normal functions. The staff cared for SARS patients for 3 months in total. | **In-service training, manpower allocation, gathering sufficient protective equipment, and establishment of a mental health team:** prevention plan based on information provided by WHO and Centers for Disease Control adjusted to conform to the hospital's environment and equipment. In-service training, manpower allocation, gathering sufficient protective equipment, and establishment of a mental health team were included. In order to guard against cross-infection in the hospital, several measures were taken with regard to handling procedure for SARS cases in general isolation room that included space, staff preparation, and environment. | Not mentioned | **Anxiety** levels 2 weeks after the implementation of the prevention program and while caring for SARS patients were significantly lower than anxiety level before caring for SARS patients (z=−2.68;p=0.075). The anxiety level month after caring for SARS patients was also significantly lower than the anxiety levels before care of SARS patients (z=−4.45;p<0.0001). After caring for SARS patients for 3 months and 1 month after the hospital returned to normal operations, nursing personnel exhibited anxiety levels significantly lower than that prior to caring for SARS patients (z=−6.58;p<0.0001).  The levels of depression 2 weeks after initiation of the SARS prevention program and while caring for SARS patients were significantly lower than the level before caring for SARS patients (z=−4.58;p<0.0001). At 1 month after caring for SARS patients, the level of depression was also significantly lower than the levels of depression levels before the staff began to care for SARS patients (z=−4.80;p<0.0001). After the hospital had returned to normal operations, the level of depression was significantly lower than that prior to taking care of SARS patients (z=−6.37;p<0.0001).  The mean score in **sleep quality** at 2 weeks after the SARS prevention program began when the staff was caring for SARS patients was significantly lower than the mean score before they began caring for SARS patients (z=−2.79;p=0.0053). At 1 month after caring for SARS patients and at 1 month after the hospital resumed normal operations, the mean score (z=−3.14;p=0.0017) was also significantly lower than the score before the nursing care began (z=−3.37;p=0.0008). |
| **Chang, Hsu and Ouyang (2022)** (185) | Taiwan, hospital  Cluster-randomized, pre- and post-test, controlled trial using parallel-groups of emergency department nurses  39 and 36 participants were recruited and allocated randomly into the intervention and control groups, respectively.  the intervention group received a 12-session workplace violence plus standard 1-hour in-service class about hospital safety policies and procedures organized by the hospital. The control group received the standard 1-hour in-service class only, which maintained their routine training class at the hospital. A video conference was used because of the COVID-19 pandemic and subdivision strategy (team-based segregation in ED).  outcome measures: Goal Commitment Scale, Occupational Coping Self-Efficacy Questionnaire for Nurses, Attitudes Toward Aggression in Emergency Room, Confidence in Managing Aggressive Behavior, Attitudes Toward Aggressive Behavior Questionnaire | **Integrated Workplace Violence Management Intervention:** 12-session course with 12 components. Each session lasted at least 1 h. The core contents of the intervention were centred first on the improvement of nurses’ awareness of workplace violence, such as identification of patients/visitors at high-risk of violent behaviour, the motivations of perpetrators, the causal factors for PVV (internal, external, and interactional causes), possible triggers of violence, and cues to impending aggression. Secondly, the intervention focused on interaction, management, prevention, and post-incident action, such as danger assessment, communication skills for a potentially threatening situation, problem solving, conflict management, and anger management. Finally, the intervention facilitated nurses to be competent in developing assertiveness techniques, engaging in more complicated interactions, and proactive violence management. | Not mentioned | Enables staff to acquire data capacities in assessment, prevention and management of **workplace violence**  The intervention had positive effects on developing stronger goal commitment, improving occupational coping self-efficacy, increasing confidence in ability to **deal with violent situations**, and modifying attitudes toward the causes and management of patient and visitor violence in Emergency Department nurses  For goal commitment, the significant group x time interaction revealed that nurses in the intervention group as compared to the control group had a greater improvement in goal commitment after completion of WPV-PMTP (Β = 4.02, p < 0.001, 95% CI = 3.13–4.91, Marginal R2 = 0.54). Moreover, nurses in the intervention group compared to the control group had a significant increase in occupational coping self-efficacy (Β = 4.83, p < 0.001, 95% CI = 2.76–6.91, Marginal R2 = 0.45). Nurses in the intervention group also had a significant increase in confidence in managing violence (Β = 4.58, p < 0.001, 95% CI = 2.71–6.45, Marginal R2 = 0.58) and attitudes toward aggressive behavior (Β = 9.15, p < 0.001, 95% CI = 7.39–10.91, Marginal R2 = 0.72). However, attitudes toward aggression in ED compared to the control group showed only a borderline of statistical significance (Β = 1.67, p = 0.050, 95% CI = −1 × 10−3–3.33, Marginal R2 = 0.29). |
| **Kostovich et al** (187)**.** | Acute care units, Hospital, USA  Mixed methods design: A one group, pretest/post-test design was used for assessing change in RNs followed by a one group, post-test only design with patients.  15 RNs working on acute care units. Nurses were included if they worked permanent full-time day or evening 8-, 10-, or 12-hour shifts; they were excluded if they were currently in orientation.  22 Patients were recruited after the nurses had completed their training. Patients were included if they met the following criteria: (a) currently being cared for by a RN who received the training, (b) anticipated discharge within 24 to 48 hours, (c) not in isolation, (d) alert and oriented to person, place, time, and (e) able to understand and answer the questions.  **Measures:** Professional Quality of Life Scale (ProQOL), Presence of Nursing Scale-RN Version (PONS-RN), Presence of Nursing Scale (PONS) Mindfulness Awareness Attention Scale (MAAS), Functional Assessment of Chronic Illness Therapy Spiritual Wellbeing (FACIT-Sp) scale, Client Satisfaction Survey (CSQ-8), Schmidt Perception of Nursing Care Survey (SPNCS) | **Internet Mantram Program:** 1-hour sessions (four individual, self-conducted modules and two synchronous group feedback sessions) completed over a three-month period. After each module, participants were asked content knowledge questions; between sessions, nurses were expected to practice Mantram Repetition Program tools. During the feedback sessions, the facilitator addressed barriers and successes of the program, answered questions, and provided feedback and suggestions for application in the workplace. | 66% completed the program  60% continued to use their Mantram, 53% used it within the past week, 60% practiced slowing down while at work | There was a significant increase in peace as a dimension of spiritual well-being, compassion satisfaction as a dimension of professional quality of life and mindfulness. There was no change in **burnout** or **secondary traumatic stress** related to professional quality of life  Mindfulness RN: pre mean (sd) 3.22 (1.18); post mean (sd) 4.42 (0.66) difference mean (sd) 1.19 (1.48) p<0.01  Professional quality of life RN: Compassion Satisfaction pre mean (sd) 41.27 (4.43) post mean (sd) 42.47 (4.48) difference mean (sd)1.20 (2.93) p=0.04;  Peace RN: pre mean (sd) 11.87 (2.44) ) post mean (sd) 13.14 (2.14) difference mean (sd) 1.28 (1.82) p=0.03 |
| **Zaghini et al (2021)** (191) | Italy, University Hospital transformed during the pandemic into a SARS-CoV-2 Hospital  Experimental mixed methods study. In the quantitative phase, a self-report questionnaire (Health and Safety Executive Management Standards Work-Related Stress Indicator Tool, Nursing Questionnaire on Organizational Health, The Nursing Quality of Life scale) was administered for detecting levels of work-related stress, job satisfaction and quality of life, before (T0) and after (T1) the implementation of the interventions.  322 nurses directly working on caring for SARS-CoV-2 positive patients. | **Proactive management interventions:** Before the arrival of the first SARS-CoV-2 patients the following interventions were made: reorganize care settings, distribution of resources to ensure adequate staffing levels, maintaining the ratio of nurses to patients, updating nurses’ clinical knowledge and abilities to comply with specific health needs of SARS-CoV-2 (191) | During the “Initial” period, there was an increase in the level of work-related stress, linked mainly to a perceived deterioration in respect to work demands. In the weeks following the very first emergency, there was an improvement in the situation and emerges the efficacy of the interventions implemented. Greater control of the working process greater possibilities for exercising one's autonomy, discretion and initiative. | Decrease in work-related **stress**, namely stress due to control, lack of support from the boss and colleagues, change and relationships. Increase in score in **job satisfaction** and **quality of life**, including quality of physical life due to physical performance and work activity would increase. Increase in **working presence** (days).  Average work-related stress score in T0 is higher than in T1, and job satisfaction and quality of life scores are on average higher in T1 than the values in T0 (respectively p <.001 and p <.01). Average satisfaction scores increased during the emergency period (p <.001); satisfaction referring specifically to the organization (p <.001) and management (p <.001). Quality of physical life due to physical performance (p = .010) and work activity (p = .005) increased. |
| **Klatt et al (2020)** (188) | Medical Center, USA  non-randomized single arm, pre/post study  Outcommes: Maslach Burnout Inventory (MBI), Perceived Stress Scale (PSS), Connor-Davidson Resilience Scale (CD-RISC), Utrecht Work Engagement Scale (UWES).  267 employees of the medical center | **Mindfulness in Motion:** replicable mindfulness intervention designed for easy delivery at work (utilizing gentle yoga stretches along with teaching mindful awareness skill development). The weekly group MIM meeting includes reflection, didactic learning about the science of mindfulness, community building, mindfulness meditation, and gentle yoga stretches, conducted with relaxing music in the background. Individual practices to be done in between the weekly meetings are audio or video practices that mimic the weekly yoga/mindfulness meditation portion of the weekly meeting, with the same music repeated in the background. These individual practices are accessed via smartphone or computer | Offering the program onsite multiple days/times per week, inclusion of interprofessional employees of the health system, and the creation of videos to ensure high fidelity of intervention delivery contributed to adherence to the program | By eight-week intervention end, there was a highly significant decrease in the in the **emotional exhaustion** and **depersonalization** with highly significant increase in the **personal accomplishment** as compared to baseline. Highly significant decrease in **stress** and increased in **resilience**.  There was a significant 27% reduction in participants meeting burnout criteria by intervention end as compared to baseline. By eight-week intervention end, there was also a highly significant decrease in the in the emotional exhaustion (p < 0.00001) and depersonalization score (p < 0.001), with highly significant increase in the personal accomplishment (p < 0.00001) Maslach Burnout Inventory subscales as compared to baseline.  The vigour subscale, representing energy levels and persistence through obstacles, showed significant increase pre to post intervention (p < 0.00001). The dedication subscale, assessing concentration and involvement in one’s work, also showed a significant increase from pre to post (p < 0.00001). Finally, the absorption subscale, measuring attachment and ability to positively get lost in one’s work, showed a significant increase pre to post intervention (p < 0.00001).  Regarding perceived stress and resilience, there was a highly significant decrease in scores on the Perceived Stress Scale (PSS) (p < 0.00001) pre-post MIM, while resilience, as measured by the Connor Davidson Resiliency Scale (CDRS) significantly increased (p < 0.0001). |
| **Lee et al (2020)** (189) | Hospital, Kazakhstan  Randomized control trial. Control group (n=60): no intervention; intervention group (n=52): short-term psychotherapy based on the coping strategy (Asimov method)  Outcomes: Maslach Emotional Burnout Questionnaire (MBI-HSS MP) | **Individual short-term psychotherapy based on Asimov’s coping strategy:** Individual short-term strategy (duration: 9-12 times a month for 50 minutes) psychotherapy (Asimov’s method) based on a coping which consists of five successive steps: i) 1st step – differentiation and self-knowledge; ii) awareness of the state through induced images; iii) awareness of the state through spontaneous images with closed eyes; iv) awareness of the state through spontaneous images with open eyes; v) environmentally friendly behaviour. Individual short-term psychotherapy based on a coping strategy as conducted (duration: 9-12 times a month for 50 minutes). Sessions of short-term psychotherapy were carried out using video communication. | Decrease uptake due to working schedule | Consistently positive effect on **burnout** (emotional exhaustion, depersonalization and professional accomplishment) and **stress**  6 months after the start of the study the EE in the intervention group (24.7±2.1), in contrast to the control (28.3±4.2) decreased (p=0.019). DP in the IG group (8.4±1.3), in contrast to the CG group (10.2±2.0) decreased (p = 0.028). PA [CG (33.1±1.9) and IG (31.9±2.8)] did not show significant differences (p = 0.067).  12 months after: EE in the IG group (18.9 ± 4.9) showed a significant decrease compared to the CG group (p = 0.017). DP in the IG group with an index of 8.4±1.3 were reduced in contrast to CG (11.2±0.9) (p = 0.01). PA in the CG group was 33.1±1.9 and in the IG group 42.2±3.9 (p = 0.021) |
| **Dincer and Inangil (2021)** (401) | University hospital, Turkey  Randomized control trial  nurses caring for COVID-19 patients: 35 in intervention group, 37 in control group  Inclusion criteria a) not having any psychiatric diagnoses, b) not taking any courses about coping with anxiety and stress, and c) volunteering to participate in the study.  Outcomes: subjective units of distress scale, State-Trait Anxiety Inventory, and Burnout Inventory | **Single online group Emotional Freedom Techniques:** session began by presenting a picture of the acupressure points and showing how to gently tap on them using index and middle fingers. After this demonstration, the participants followed the basic steps of an emotional freedom technique session: i) identify an anxiety-evoking issue; ii) creating a personal acceptance and reminder statement in the general form of "I accept myself despite this…"; iii) Tapping seven times on each acupressure point; iv) After tapping these points, the affirmation/reminder statement is repeated; v) A sequence of physical movements and vocalizations called “The Nine Gamut Procedure” is carried out; vi) Steps 3 and 4 are repeated. | Not mentioned | Reduction in the levels of **stress**, **anxiety** and **burnout**.  Mean stress score reduction on the post-test for the intervention group was highly significant (IG 2.85 ± 1.21 vs CG 7.40 ± 1.53; p<.001).  Mean anxiety score reduction on the post-test for the intervention group was highly significant (IG 32.25 ± 4.67 vs 64.43 ± 7.68; p<.001)  Mean burnout score reduction on the post-test for the intervention group was highly significant (IG 2.48 ± 1.06 vs 3.43 ± 0.76; p<.001)) |
| **Waterman et al (2018)** (402) | Ebola Treatment Centres, Sierra Leone  Pre post intervention study  Outcomes: Generalised Anxiety Disorder 7 (GAD7), Patient Health Questionnaire 9 (PHQ9), Post-Traumatic Stress Checklist - Civilian version (PCL-C), Perceived Stress Scale (PSS), Insomnia Severity Index (ISI) | **Cognitive behavioural therapy for anxiety and depression:** Phase 1: 2-hour workshop based on the concept of Psychological First Aid allowed to discuss challenges and impact of work, ways of coping achievements. Participants were referred to phase 2 as necessary following completion of the screening questionnaire at phase 1, but they were also able to attend any other sessions if they so wished. Phase 2: 2-hour workshops which focused on one of six different common mental health difficulties. Each of the Phase 2 workshops focussed on psychoeducation about the specific problem, followed by discussion of a range of simple coping strategies based on behavioural and cognitive approaches that staff could use as self-help. If participants met or exceeded total GAD-7 and PHQ-9 scores of 8 and 10 respectively, or if they had a combined score of 21 or above they were considered eligible and were invited to attend phase 3 Phase 3: participants were in small groups and met on a weekly basis with their facilitators who guided them through a low intensity cognitive behavioural therapy programme that included behavioural activation, minimising avoidance, problem solving and coping with anxiety (402) | The uptake seen throughout the intervention also demonstrates the willingness of staff to attend a mental health intervention, which had been a concern prior to conducting the study given the high levels of mental Health stigma  This research demonstrated the feasibility of delivering this type of intervention by training in-country staff as facilitators | Each phase of the 3-phase intervention for **depression** and **anxiety** appears to have been effective in reducing mental health symptoms.  Brief Cognitive behavioural therapy-based interventions targeting depression and anxiety amongst health care workers providing emergency response can be beneficial in reducing clinical symptoms.  Phase 2 significant improvement in measures of stress (27.77 ± 7.63 vs 23.37 ± 6.02; t(29) = 2.26; p < .05), anxiety (16.88 ± 3.83 vs 13.76 ± 6.77; t(36) = 2.55; p < 0.05), depression (22.10 ± 4.31 vs 15.56 ± 9,16; t(33) = 3.83; p < .01), behavoural problems (1.30 ± 1.34 vs 0.53 ± 1.11; t(29) = 3.16; p < .05) and alcohol usage (3.69 ± 4.53 vs 1.54 ± 2.86; t(25) = 2.48; p < .05).  Phase 3 Wellbeing Screening Measure (44.61 ± 16.05 vs 33.93 ± 15.75; t(73) = 4.69; p < .01) and clinical measures for PTSD (59.39 ± 17.86 vs 46.41 ± 19.53; t(70) = 4.16; p < .01), stress (23.58 ± 5.50 vs 20.58 ± 4.44; t(73) = 3.66; p < .01), sleep (24.23 ± 8.91 vs 19.60 ± 7.63; t(73) = 3.38; p < .01), anxiety (13.52 ± 6.35 vs 10.40 ± 6.48; t(72) = 2.93; p < .05), depression (15.32 ± 8.23 vs 12.60 ± 7.70; t(72) = 7.14 p < .05), anger (10.60 ± 6.11 vs 7.43 ± 5.87; t(73) = 3.40; p < .01) and relationship difficulties (27.61 ± 5.87 vs 23.78 ± 6.05; t(73) = 4.25; p < .01)  Measure from phase 1 to end of phase 3, relating to stress (F(3, 51) = 7.89; p < .01), depression (F(3, 84) = 11.68; p < .01), anxiety (F(3, 78) = 3.40; p < .05), behaviour (F(3, 84) = 6.08; p < .01) and relation-ships (F(3, 69) = 3.72; p < .05). There were no significant differences in sleep. |
