# Supplementary material for: Impacts for health and care workers of Covid-19 and other public health emergencies of international concern: living systematic review, meta-analysis and policy recommendations
Source: Hum Resour Health. 2024 Jan 25;22:10. doi: 10.1186/s12960-024-00892-2 (PMC10809470; doi:10.1186/s12960-024-00892-2)
Supplement: Supplementary file 6 — Additional file 6. Forest plots and funnel plots for meta-analysis of the outcomes. [file 12960_2024_892_MOESM6_ESM.docx]

## Search strategy

| **DATABASE** | **SEARCH STRATEGIES** | **N** |
| --- | --- | --- |
| **PUBMED** | (((COVID-19[mj] OR SARS-CoV-2[mj] OR Severe Acute Respiratory Syndrome Coronavirus 2[tiab] OR Coronavirus Disease 2019[tiab] OR 2019 Novel Coronavirus[tiab] OR 2019 New Coronavirus[tiab] OR Wuhan Coronavirus[tiab] OR COVID-19[tiab] OR SARS-CoV-2[tiab] OR 2019-nCoV[tiab] OR HCoV-19[tiab] OR nCoV-2019[tiab] OR Novel Coronavirus 2019-nCoV[tiab] OR Alpha Variant[tiab] OR Beta Variant[tiab] OR Gamma Variant[tiab] OR Delta Variant[tiab] OR Delta Plus Variant[tiab] OR Omicron Variant[tiab] OR Lambda Variant[tiab] OR Influenza A Virus[mj] OR Influenza A Virus*[tiab] OR Influenza Viruses Type A[tiab] OR Middle East Respiratory Syndrome Coronavirus[mj] OR Middle East Respiratory Syndrome Coronavirus[tiab] OR Middle East Respiratory Syndrome Related Coronavirus[tiab] OR MERS[tiab] OR MERS-CoV[tiab] OR Hemorrhagic Fevers, Viral[mj] OR Viral Hemorrhagic Fever*[tiab] OR SARS Virus[mj] OR Severe Acute Respiratory Syndrome Virus[tiab] OR SARS[tiab] OR SARS-COV[tiab] OR Influenza A Virus, H1N1 Subtype[mj] OR H1N1 Virus*[tiab] OR Hemorrhagic Fever, Ebola[mj] OR Ebola[tiab] OR Zika Virus Infection[mj] OR Zika[tiab] OR Public Health Emergenc*[tiab] OR Public Health Emergency Preparedness[tiab] OR COVID[tiab] OR Pandemia[tiab] OR Pandemic*[tiab]) AND (Health Workforce[mj] OR Workforce*[tiab] OR Health Manpower[tiab] OR Health Personnel[mj] OR Health Personnel*[tiab] OR Health Care Provider*[tiab] OR Healthcare Provider*[tiab] OR Health Care Worker*[tiab] OR Healthcare Worker*[tiab] OR Health Care Professional*[tiab] OR Healthcare Professional*[tiab] OR Human Resources for Health[tiab] OR Health Worker*[tiab] OR Allied Health Professional*[tiab] OR Healthcare Assistant*[tiab] OR Health Care Assistant*[tiab] OR Healthcare Support Worker*[tiab] OR Health Care Support Worker*[tiab] OR Caregivers[mj] OR Caregiver*[ti] OR Licensed Practical Nurses[mj] OR Nursing Staff[mj] OR Nurses[mj] OR Nurse*[ti] OR Nursing Personnel*[tiab] OR Nursing Staff*[ti] OR Professional Nurse*[ti] OR Nursing Associate*[ti] OR Nursing Professional*[ti] OR Nursing Assistant*[ti] OR Auxiliary Nurse*[ti] OR Nursing Auxiliar*[ti] OR Licensed Practical Nurse*[ti] OR Nursing Team*[ti] OR Dentist*[ti] OR Doctor*[ti] OR Physicians[mj] OR Physician*[ti] OR Pharmacist*[ti] OR Physiotherapist*[ti] OR Midwive*[ti] OR Community Health Worker*[tiab] OR Community-Based Provider*[tiab] OR Laboratory Staff*[tiab] OR Paramedical Staff*[tiab] OR Paramedical Personnel*[tiab] OR Paramedic*[ti] OR Population Program Specialist*[tiab])) AND (Mental Health[mj] OR Mental Health[ti] OR Psychological Distress[mj] OR Psychological Distress*[ti] OR Emotional Distress*[ti] OR Emotional Stress*[ti] OR Behavioral Symptoms[mj] OR Behavioral Symptom*[ti] OR Affective Symptom*[ti] OR Aggression*[ti] OR Agonistic Behavior[ti] OR Bullying[ti] OR Catatonia[ti] OR Delusion*[ti] OR Depersonalization[ti] OR Depression[ti] OR Malingering[ti] OR Mental Fatigue[ti] OR Alert Fatigue[ti] OR Compassion Fatigue[ti] OR Obsessive Behavior[ti] OR Stalking[ti] OR Paranoid Behavior[ti] OR Psychogenic Polydipsia[ti] OR Problem Behavior[ti] OR Schizophrenic Language[ti] OR Self-Injurious Behavior[ti] OR Self-Mutilation*[ti] OR Suicide[ti] OR Psychological Burnout[ti] OR Caregiver Burden[ti] OR Financial Stress*[ti] OR Occupational Stress*[ti] OR Wandering Behavior[ti] OR Substance-Related Disorders[mj] OR Substance-related Disorder*[ti] OR Drug Use Disorder*[ti] OR Substance Abuse*[ti] OR Substance Dependence[ti] OR Chemical Dependence[ti] OR Drug Dependence[ti] OR Prescription Drug Abuse[ti] OR Substance Use[ti] OR Drug Abuse*[ti] OR Drug Habituation[ti] OR Personal Protective Equipment[mj] OR Personal Protective Equipment*[ti] OR PPE[ti] OR Stress Disorders, Post-Traumatic[mj] OR Post-Traumatic Stress Disorder*[ti] OR Post-Traumatic Neurose*[ti] OR PTSD[ti] OR Posttraumatic Neurose*[ti] OR Chronic Post-Traumatic Stress Disorder*[ti] OR Moral Injur*[ti] OR Acute Post-Traumatic Stress Disorder*[ti] OR Social Discrimination[mh] OR Social Discrimination*[ti] OR Disability Discrimination*[ti] OR Ableism[ti] OR Social Stigma[mj] OR Social Stigma*[ti] OR Workplace Violence[mj] OR Workplace Violence*[ti] OR Hazardous Substances[mj] OR Hazardous Substance*[ti] OR Hazardous Material*[ti] OR Hazardous Chemical*[ti] OR Environmental Toxic Substance*[ti] OR Biohazard*[ti] OR Quality of Life[mj] OR Quality of Life[ti] OR Health Related Quality Of Life[ti] OR HRQOL[ti] OR Work Performance[mj] OR Work Performance*[ti] OR Job Performance[ti] OR Absenteeism[mj] OR Absenteeism[ti] OR Personnel Turnover[mj] OR Personnel Turnover*[ti] OR Employee Turnover*[ti] OR Attitude of Health Personnel[mj] OR Attitude of Health Personnel*[ti] OR Staff Attitude*[ti] OR Mortality[mj] OR Mortalit*[ti] OR Case Fatality Rate*[ti] OR Death Rate*[ti])) NOT (Letter*[tw] OR Editorial*[tw] OR Release*[tw]) AND (English[lang] OR Portuguese[lang] OR Spanish[lang] OR French[lang] OR Italian[lang] OR Hindi[lang]) AND ("2000/01/01"[PDAT] : "2022/03/01"[PDAT]) | **3872** |
| **EMBASE** | ('coronavirus disease 2019'/exp OR '2019 novel coronavirus':ti,ab OR '2019-ncov':ti,ab OR 'covid 19':ti,ab OR 'covid 2019':ti,ab OR 'covid-19':ti,ab OR 'sars coronavirus 2':ti,ab OR 'sars-cov-2':ti,ab OR 'wuhan coronavirus':ti,ab OR 'coronavirus disease 2019':ti,ab OR 'coronavirus infection 2019':ti,ab OR 'ncov 2019':ti,ab OR 'new coronavirus':ti,ab OR 'novel coronavirus 2019':ti,ab OR 'novel coronavirus':ti,ab OR 'severe acute respiratory syndrome 2':ti,ab OR 'covid':ti,ab OR 'Alpha Variant':ti,ab OR 'Beta Variant':ti,ab OR 'Gama Variant':ti,ab OR 'Delta Variant':ti,ab OR 'Delta Plus Variant':ti,ab OR 'Omicron Variant':ti,ab OR 'Lambda Variant':ti,ab OR 'pandemia':ti,ab OR 'pandemic*':ti,ab OR 'severe acute respiratory syndrome'/mj OR 'sars':ti,ab OR 'sars coronavirus infection':ti,ab OR 'sars-cov infection':ti,ab OR 'sars-associated coronavirus infection':ti,ab OR 'sars-related coronavirus infection':ti,ab OR 'severe acute respiratory syndrome':ti,ab OR 'middle east respiratory syndrome'/mj OR 'mers coronavirus infection':ti,ab OR 'mers infection':ti,ab OR 'mers virus infection':ti,ab OR 'mers-cov infection':ti,ab OR 'middle east respiratory syndrome':ti,ab OR 'middle east respiratory syndrome coronavirus infection':ti,ab OR 'middle east respiratory syndrome infection':ti,ab OR 'virus hemorrhagic fever'/mj OR 'epidemic haemorrhagic fever':ti,ab OR 'epidemic hemorrhagic fever':ti,ab OR 'viral haemorrhagic fevers':ti,ab OR 'viral hemorrhagic fevers':ti,ab OR 'virus haemorrhagic fever':ti,ab OR 'virus hemorrhagic fever':ti,ab OR 'influenza a virus (h1n1)'/mj OR 'h1n1':ti,ab OR 'h1n1 influenza a virus':ti,ab OR 'h1n1 influenza virus':ti,ab OR 'h1n1 subtype':ti,ab OR 'h1n1 virus':ti,ab OR 'virus h1n1':ti,ab OR 'ebola hemorrhagic fever'/mj OR 'ebola':ti,ab OR 'ebola fever':ti,ab OR 'ebola hemorrhagic fever':ti,ab OR 'zika fever'/mj OR 'zikv infection':ti,ab OR 'zika fever':ti,ab OR 'zika virus infection':ti,ab) AND ('health workforce'/mj OR 'health care labour force':ti,ab OR 'health care manpower':ti,ab OR 'health care work force':ti,ab OR 'health care workforce':ti,ab OR 'health labor force':ti,ab OR 'health labour force':ti,ab OR 'health manpower':ti,ab OR 'health work force':ti,ab OR 'health workforce*':ti,ab OR 'healthcare labor force':ti,ab OR 'healthcare labour force':ti,ab OR 'healthcare manpower':ti,ab OR 'healthcare work force':ti,ab OR 'healthcare workforce':ti,ab OR 'health care labor force':ti,ab OR 'health care personnel'/mj OR 'health care personnel*':ti,ab OR 'health care practitioner*':ti,ab OR 'health care professional*':ti,ab OR 'health care provider*':ti,ab OR 'health care worker*':ti,ab OR 'health personnel*':ti,ab OR 'health worker*':ti,ab OR 'healthcare personnel':ti,ab OR 'healthcare practitioner':ti,ab OR 'healthcare professional':ti,ab OR 'healthcare provider':ti,ab OR 'healthcare worker*':ti,ab OR 'caregiver'/mj OR 'caregiver*':ti,ab OR 'human resources for health':ti,ab OR 'licensed practical nurse'/mj OR 'licensed practical nurse*':ti,ab OR 'licensed vocational nurse':ti,ab OR 'nursing staff'/mj OR 'hospital nursing staff':ti,ab OR 'nurse staffing':ti,ab OR 'nursing manpower':ti,ab OR 'nursing personnel':ti,ab OR 'nursing staff':ti,ab OR 'nurse'/mj OR 'nurse*':ti,ab OR 'nursing associate*':ti,ab OR 'nursing assistant'/mj OR 'nursing assistant*':ti,ab OR 'auxiliary nurse*':ti,ab OR 'nursing auxiliar*':ti,ab OR 'team nursing'/mj OR 'team nursing':ti,ab OR 'dentist'/mj OR 'dentist*':ti,ab OR 'physician'/mj OR 'doctor*':ti,ab OR 'physician*':ti,ab OR 'private physician':ti,ab OR 'pharmacist'/mj OR 'pharmacist*':ti,ab OR 'physiotherapist'/mj OR 'physical therapist*':ti,ab OR 'physiotherapist*':ti,ab OR 'midwife'/mj OR 'midwife':ti,ab OR 'midwifery':ti,ab OR 'midwives':ti,ab OR 'health auxiliary'/mj OR 'auxiliary health worker':ti,ab OR 'community health worker*':ti,ab OR 'medical auxiliary':ti,ab OR 'community-based provider*':ti,ab OR 'laboratory staff*':ti,ab OR 'paramedical personnel'/mj OR 'healthcare assistant':ti,ab OR 'healthcare support worker':ti,ab OR 'para medical personnel':ti,ab OR 'paramedical personnel':ti,ab OR 'paramedical professional':ti,ab OR 'paramedical staff':ti,ab) AND ('mental health'/mj OR 'mental condition':ti OR 'mental health':ti OR 'distress syndrome'/mj OR 'distress':ti OR 'distress syndrome':ti OR 'dystress syndrome':ti OR 'psychological distress':ti OR 'behavior'/mj OR 'behavior':ti OR 'behavioral symptoms':ti OR 'behaviour':ti OR 'behavioural symptoms':ti OR 'emotional disorder'/mj OR 'affective symptoms':ti OR 'emotion disorder':ti OR 'emotional disorder':ti OR aggression*:ti OR 'agonistic behavior':ti OR 'bullying'/mj OR 'bullying':ti OR 'catatonia'/mj OR 'catatonia':ti OR 'catatony':ti OR delusion*:ti OR depersonalization:ti OR 'depression'/mj OR 'depression':ti OR 'depressive disease':ti OR 'depressive disorder':ti OR 'depressive state':ti OR 'depressive symptom':ti OR 'depressive syndrome':ti OR malingering:ti OR 'mental fatigue'/mj OR 'mental fatigue':ti OR 'alert fatigue (health care)'/mj OR 'health personnel alert fatigue':ti OR 'compassion fatigue':ti OR 'obsessive behavior':ti OR 'paranoid behavior':ti OR 'psychogenic polydipsia':ti OR 'problem behavior':ti OR 'schizophrenic language':ti OR 'automutilation'/mj OR 'automutilation':ti OR 'self injury':ti OR 'self mutilation':ti OR 'self-injuring behaviour':ti OR 'self-injurious behavior':ti OR 'self-injurious behaviour':ti OR 'selfmutilation':ti OR 'self directed violence':ti OR 'self-mutilation':ti OR 'suicide'/mj OR 'suicidal death':ti OR 'suicide':ti OR 'suicide death':ti OR 'burnout'/mj OR 'burn-out':ti OR 'burnout':ti OR 'burnout syndrome':ti OR 'psychological burn-out':ti OR 'psychological burnout':ti OR 'caregiver burden'/mj OR 'caregiver burden':ti OR 'caregiver strain':ti OR 'financial stress'/mj OR 'financial stress':ti OR 'job stress'/mj OR 'job related stress':ti OR 'job stress':ti OR 'occupational stress':ti OR 'professional stress':ti OR 'work related stress':ti OR 'work stress':ti OR 'wandering behavior'/mj OR 'wandering behavior':ti OR 'wandering behaviour':ti OR 'drug dependence'/mj OR 'drug addict':ti OR 'drug addiction':ti OR 'drug dependence':ti OR 'drug dependency':ti OR 'drug habituation':ti OR 'drug physical dependence':ti OR 'substance addiction':ti OR 'substance dependence':ti OR 'substance dependency':ti OR 'substance use disorder':ti OR 'substance use disorders':ti OR 'substance-related disorder':ti OR 'substance-related disorders':ti OR 'prescription drug abuse':ti OR 'protective equipment'/mj OR 'ppe (personal protective equipment)':ti OR 'personal protection equipment':ti OR 'personal protective equipment':ti OR 'protective devices':ti OR 'protective equipment':ti OR 'posttraumatic stress disorder'/mj OR 'ptsd':ti OR 'ptsd (posttraumatic stress disorder)':ti OR 'post-traumatic stress':ti OR 'post-traumatic stress disorder':ti OR 'post-traumatic stress disorders':ti OR 'posttraumatic neurosis':ti OR 'posttraumatic psychic syndrome':ti OR 'posttraumatic stress':ti OR 'posttraumatic stress disorder':ti OR 'posttraumatic syndrome':ti OR 'traumatic stress':ti OR 'traumatic stress disorder':ti OR 'traumatic stress disorders':ti OR 'moral injur*':ti OR 'acute post-traumatic stress disorder*':ti OR 'social discrimination'/mj OR 'social discrimination':ti OR 'disability discrimination'/mj OR 'able-ism':ti OR 'ableism':ti OR 'disability discrimination':ti OR 'disability-based discrimination':ti OR 'disability-related discrimination':ti OR 'disableism':ti OR 'social stigma'/mj OR 'social stigma':ti OR 'social stigmatisation':ti OR 'social stigmatization':ti OR 'workplace violence'/mj OR 'workplace violence':ti OR 'hazardous substances':ti OR 'hazardous material*':ti OR 'hazardous chemical*':ti OR 'environmental toxic substance*':ti OR biohazard*:ti OR 'quality of life'/mj OR 'hrql':ti OR 'health related quality of life':ti OR 'life quality':ti OR 'quality of life':ti OR 'job performance'/mj OR 'job performance':ti OR 'work performance':ti OR 'working performance':ti OR 'absenteeism'/mj OR 'absenteeism':ti OR 'disability absence':ti OR 'work absence':ti OR 'work absenteeism':ti OR 'work day loss':ti OR 'work time loss':ti OR 'personnel management'/mj OR 'personnel management':ti OR 'personnel turnover':ti OR 'staff management':ti OR 'employee turnover*':ti OR 'health personnel attitude'/mj OR 'attitude of health personnel':ti OR 'health care personnel attitude':ti OR 'health personnel attitude':ti OR 'healthcare personnel attitude':ti OR 'staff attitude*':ti OR 'mortality'/mj OR 'mortality':ti OR 'mortality rate'/mj OR 'death rate':ti OR 'fatality rate':ti OR 'mortality rate':ti) NOT (letter*:ti,ab,kw OR editorial*:ti,ab,kw OR release*:ti,ab,kw) AND ([english]/lim OR [french]/lim OR [hindi]/lim OR [italian]/lim OR [portuguese]/lim OR [spanish]/lim) AND [01-01-2000]/sd NOT [01-03-2022]/sd AND [embase]/lim NOT ([embase]/lim AND [medline]/lim) | **1224** |
| **SCOPUS** | ALL(COVID-19 OR SARS-CoV-2 OR "Severe Acute Respiratory Syndrome Coronavirus 2" OR "Coronavirus Disease 2019" OR "2019 Novel Coronavirus" OR "2019 New Coronavirus" OR "Wuhan Coronavirus" OR COVID-19 OR SARS-CoV-2 OR 2019-nCoV OR HCoV-19 OR nCoV-2019 OR "Novel Coronavirus 2019-nCoV" OR "Alpha Variant" OR "Beta Variant" OR "Gamma Variant" OR "Delta Variant" OR "Delta Plus Variant" OR "Omicron Variant" OR "Lambda Variant" OR "Influenza A Virus" OR "Influenza Viruses Type A" OR "Middle East Respiratory Syndrome Coronavirus" OR "Middle East Respiratory Syndrome Related Coronavirus" OR MERS OR MERS-CoV OR "Viral Hemorrhagic Fever" OR "SARS Virus" OR "Severe Acute Respiratory Syndrome Virus" OR SARS OR SARS-COV OR "H1N1 Virus" OR Ebola OR "Zika Virus Infection" OR Zika OR "Public Health Emergencies" OR "Public Health Emergency Preparedness" OR COVID OR Pandemia OR Pandemic*) AND TITLE("Health Care Providers" OR "Healthcare Providers" OR "Healthcare Workers" OR "Health Care Workers" OR "Health Care Professionals" OR "Healthcare Professionals" OR "Health Workers" OR "Health Personnel" OR "Health Professionals" OR Physician* OR "Allied Health Personnel" OR "Allied Health Professionals" OR "Healthcare Assistants" OR "Health Care Assistants" OR "Healthcare Support Workers" OR "Health Care Support Workers" OR Paramedic* OR "Population Program Specialists" OR "Community Health Workers" OR "Community-Based Providers" OR Caregiver* OR "Licensed Practical Nurses" OR "Nursing Staff" OR Nurse* OR "Nursing Personnel" OR "Professional Nurses" OR "Nursing Associate" OR "Nursing Professionals" OR "Nursing Assistant" OR "Auxiliary Nurses" OR "Nursing Auxiliary" OR "Licensed Practical Nurses" OR "Nursing Team" OR Dentists OR Doctors OR Physicians OR Pharmacists OR Physiotherapists OR Midwives OR "Community Health Workers" OR "Community-Based Providers" OR "Laboratory Staff" OR "Paramedical Staff" OR "Paramedical Personnel") AND TITLE("Mental Health" OR "Psychological Distress" OR "Emotional Distress" OR "Emotional Stress" OR "Behavioral Symptoms" OR "Affective Symptoms" OR Aggression* OR "Agonistic Behavior" OR Bullying OR Catatonia OR Delusion* OR Depersonalization OR Depression OR Malingering OR "Mental Fatigue" OR "Alert Fatigue" OR "Compassion Fatigue" OR "Obsessive Behavior" OR Stalking OR "Paranoid Behavior" OR "Psychogenic Polydipsia" OR "Problem Behavior" OR "Schizophrenic Language" OR "Self-Injurious Behavior" OR "Self-Mutilation" OR Suicide OR "Psychological Burnout" OR "Caregiver Burden" OR "Financial Stress" OR "Occupational Stress" OR "Wandering Behavior" OR "Substance-Related Disorders" OR "Drug Use Disorders" OR "Substance Abuse" OR "Substance Dependence" OR "Chemical Dependence" OR "Drug Dependence" OR "Prescription Drug Abuse" OR "Substance Use" OR "Drug Abuse" OR "Drug Habituation" OR "Personal Protective Equipment" OR PPE OR "Post-Traumatic Stress Disorders" OR "Post-Traumatic Neuroses" OR PTSD OR "Posttraumatic Neuroses" OR "Chronic Post-Traumatic Stress Disorders" OR "Moral Injury" OR "Acute Post-Traumatic Stress Disorders" OR "Social Discrimination" OR "Disability Discrimination" OR Ableism OR "Social Stigma" OR "Workplace Violence" OR "Hazardous Substances" OR "Hazardous Materials" OR "Hazardous Chemicals" OR "Environmental Toxic Substances" OR Biohazard* OR "Quality of Life" OR "Health Related Quality Of Life" OR HRQOL OR "Work Performance" OR "Job Performance" OR Absenteeism OR Absenteeism OR "Personnel Turnover" OR "Employee Turnover" OR "Attitude of Health Personnel" OR "Staff Attitude" OR Mortality OR Mortalit* OR "Case Fatality Rate" OR "Case Fatality Rates" OR "Death Rate" OR "Death Rates") AND (LIMIT-TO(DOCTYPE, "ar")) AND (LIMIT-TO(LANGUAGE, "English") OR LIMIT-TO(LANGUAGE, "Spanish") OR LIMIT-TO(LANGUAGE, "French") OR LIMIT-TO(LANGUAGE, "Portuguese")) OR LIMIT-TO(LANGUAGE, "Italian")) OR LIMIT-TO(LANGUAGE, "Hindi")) AND (LIMIT-TO(PUBYEAR, 2022) OR LIMIT-TO(PUBYEAR, 2021) OR LIMIT-TO(PUBYEAR, 2020) OR (LIMIT-TO(PUBYEAR, 2019) OR LIMIT-TO(PUBYEAR, 2018) OR LIMIT-TO(PUBYEAR, 2017) OR (LIMIT-TO(PUBYEAR, 2016) OR LIMIT-TO(PUBYEAR, 2015) OR LIMIT-TO(PUBYEAR, 2014) OR (LIMIT-TO(PUBYEAR, 2013) OR LIMIT-TO(PUBYEAR, 2012) OR LIMIT-TO(PUBYEAR, 2011) OR (LIMIT-TO(PUBYEAR, 2010) OR LIMIT-TO(PUBYEAR, 2009) OR LIMIT-TO(PUBYEAR, 2008) OR (LIMIT-TO(PUBYEAR, 2007) OR LIMIT-TO(PUBYEAR, 2006) OR LIMIT-TO(PUBYEAR, 2005) OR (LIMIT-TO(PUBYEAR, 2004) OR LIMIT-TO(PUBYEAR, 2003) OR LIMIT-TO(PUBYEAR, 2002) OR (LIMIT-TO(PUBYEAR, 2001) OR LIMIT-TO(PUBYEAR, 2000)) | **1178** |
| **LILACS** | (covid-19 OR sars-cov-2 OR "Severe Acute Respiratory Syndrome Coronavirus 2" OR "Coronavirus Disease 2019" OR "2019 Novel Coronavirus" OR "2019 New Coronavirus" OR "Wuhan Coronavirus" OR 2019-ncov OR hcov-19 OR ncov-2019 OR "Novel Coronavirus 2019-nCoV" OR "Influenza A Virus" OR "Middle East Respiratory Syndrome Coronavirus" OR MERS OR "Viral Hemorrhagic Fever" OR "SARS Virus" OR "Severe Acute Respiratory Syndrome Virus" OR SARS OR "H1N1 Virus" OR Ebola OR zika OR "Public Health Emergencies" OR covid OR pandemia* OR pandemic* OR "Sindrome Respiratória Aguda Grave 2" OR "Novo Coronavirus" OR "Alpha Variant" OR "Beta Variant" OR "Gamma Variant" OR "Delta Variant" OR "Delta Plus Variant" OR "Omicron Variant" OR "Lambda Variant") AND ("Health Workforce" OR "Health Manpower" OR "Health Personnel" OR "Health Personnels" OR "Health Care Providers" OR "Healthcare Providers" OR "Health Care Workers" OR "Healthcare Workers" OR "Health Care Professionals" OR "Healthcare Professionals" OR caregiver* OR Nursing OR Nurse* OR Dentist* OR Doctor* OR Physician* OR Pharmacist* OR Physiotherapist* OR Midwive* OR "Community Health Workers" OR "Community-Based Providers" OR "Laboratory Staff" OR Paramedical OR "força de trabalho em saúde" OR "recursos humanos em saúde" OR "pessoal de saúde" OR "provedores de saúde" OR "trabalhadores em saúde" OR "profissionais de saúde" OR cuidador* OR enfermeir* OR enfermagem OR dentista* OR médico* OR farmacêutico* OR fisioterapeuta* OR parteira* OR "trabalhadores comunitários de saúde" OR "provedores de base comunitária" OR "equipe de laboratório" OR paramédico*) AND (ti:("Mental Health" OR "Psychological Distress" OR "Emotional Distress" OR "Emotional Stress" OR "Behavioral Symptoms" OR "Affective Symptoms" OR Aggression* OR "Agonistic Behavior" OR bullying OR catatonia OR delusion* OR depersonalization OR depression OR malingering OR "Mental Fatigue" OR "Alert Fatigue" OR "Compassion Fatigue" OR "Obsessive Behavior" OR stalking OR "Paranoid Behavior" OR "Psychogenic Polydipsia" OR "Problem Behavior" OR "Schizophrenic Language" OR "Self-Injurious Behavior" OR "Self-Mutilation" OR suicide OR "Psychological Burnout" OR "Caregiver Burden" OR "Financial Stress" OR "Occupational Stress" OR "Wandering Behavior" OR "Substance-Related Disorders" OR "Drug Use Disorders" OR "Substance Abuse" OR "Substance Dependence" OR "Chemical Dependence" OR "Drug Dependence" OR "Prescription Drug Abuse" OR "Substance Use" OR "Drug Abuse" OR "Drug Habituation" OR "Personal Protective Equipment" OR PPE OR "Post-Traumatic Stress Disorders" OR "Post-Traumatic Neuroses" OR PTSD OR "Posttraumatic Neuroses" OR "Chronic Post-Traumatic Stress Disorders" OR "Moral Injury" OR "Acute Post-Traumatic Stress Disorders" OR "Social Discrimination" OR "Disability Discrimination" OR ableism OR "Social Stigma" OR "Workplace Violence" OR "Hazardous Substances" OR "Hazardous Materials" OR "Hazardous Chemicals" OR "Environmental Toxic Substances" OR biohazard* OR "Quality of Life" OR "Health Related Quality Of Life" OR HRQOL OR "Work Performance" OR "Job Performance" OR absenteeism OR "Personnel Turnover" OR "Employee Turnover" OR "Attitude of Health Personnel" OR "Staff Attitude" OR "Saúde mental" OR "Angústia psicológica" OR "Angústia emocional" OR "Estresse emocional" OR "Sintomas comportamentais" OR "Sintomas afetivos" OR agressão OR "Comportamento agonista" OR intimidação OR catatonia OR delusão OR despersonalização OR depressão OR simulação OR "Fadiga mental" OR "Fadiga de alerta" OR "Fadiga de compaixão" OR "Comportamento obsessivo" OR perseguição OR "Comportamento paranóico" OR "Polidipsia psicogênica" OR "Comportamento problemático" OR "Linguagem esquizofrênica" OR "Comportamento autoagressor" OR "Auto-mutilação" OR suicídio OR "Desgaste psicológico" OR "Carga do cuidador" OR "Estresse financeiro" OR "Estresse ocupacional" OR "Comportamento errante" OR "Transtornos relacionados ao uso de substâncias" OR "Transtornos do uso de drogas" OR "Abuso de substâncias" OR "Dependência de substância" OR "Dependência química" OR "Dependência de drogas" OR "Abuso de medicamentos prescritos" OR "Uso de substâncias" OR "Abuso de drogas" OR "Habituação a drogas" OR "Equipamento de proteção pessoal" OR "Equipamento de proteção individual" OR EPI OR "Estresse pós-traumático Distúrbios" OR "Neuroses pós-traumáticas" OR "Transtornos de estresse pós-traumático crônico" OR "Lesão moral" OR "Transtornos de estresse pós-traumático agudo" OR "Discriminação social" OR "Discriminação por deficiência" OR ableismo OR "Estigma social" OR "Violência no local de trabalho" OR "Substâncias perigosas" OR "Materiais perigosos" OR "Produtos químicos perigosos" OR "Substâncias tóxicas para o meio ambiente" OR "risco biológico" OR "Qualidade de vida" OR "Qualidade de vida relacionada à saúde" OR QVRS OR "Desempenho no trabalho" OR absentismo OR "Rotatividade de pessoal" OR "Rotatividade de funcionários" OR "Atitude do pessoal de saúde" OR "Salud mental" OR "Estrés emocional" OR agresión* OR "Comportamiento agonista" OR intimidación OR delirio OR despersonalización OR depresión OR "Fatiga mental" OR "Comportamiento obsesivo" OR "Comportamiento paranoico" OR "Polidipsia psicógena" OR "Lenguaje esquizofrénico" OR "Comportamiento autodestructivo" OR autolesión OR "Tensión psicológica" OR "Estrés financiero" OR "Estrés ocupacional" OR "Conducta errante" OR "Trastornos por consumo de sustancias" OR "Trastornos por consumo de drogas" OR "Abuso de sustancias" OR "Dependencia de sustancias" OR "Dependencia de sustancias químicas" OR "Dependencia de drogas" OR "Abuso de medicamentos recetados" OR "Uso de sustancias" OR "Equipaje de protección personal" OR "Trastornos de estrés postraumático" OR "Neurosis postraumática" OR TEPT OR "Trastornos de estrés postraumático crónico" OR "Lesión moral" OR "Trastornos de estrés postraumático" OR "Discriminación social" OR "Discriminación por discapacidad" OR "Violencia en el lugar de trabajo" OR "Sustancias peligrosas" OR "Materiales peligrosos" OR "Productos químicos peligrosos" OR "Sustancias tóxicas para el medio ambiente" OR "peligro biológico" OR "Calidad de vida" OR "Calidad de vida relacionada con la salud" OR CVRS OR "Rendimiento en el trabajo" OR ausentismo OR "Rotación de personal" OR "Actitud de personal sanitario" OR Mortalit* OR Mortalid* OR "Case Fatality Rate" OR "Death Rate")) AND (db:("LILACS")) AND (year_cluster:[2000 TO 2022]) | **166** |
| **WHO COVID-19** | ("COVID 19" OR "SARS CoV 2" OR "Alpha Variant" OR "Beta Variant" OR "Gamma Variant" OR "Delta Variant" OR "Delta Plus Variant" OR "Omicron Variant" OR "Lambda Variant" OR "Influenza A virus" OR "Middle East Respiratory Syndrome" OR Coronavirus OR "Hemorrhagic Fevers Viral" OR "SARS Virus" OR Ebola OR "Zika Virus" OR Pandemic) AND ("Health Personnel" OR "Health Workers") AND db:("PREPRINT-MEDRXIV" OR "COVIDWHO") | **318** |
| **CLINICAL TRIALS** | ("COVID 19" OR "SARS CoV 2" OR "Middle East Respiratory Syndrome" OR Coronavirus OR "Hemorrhagic Fevers Viral" OR "SARS Virus" OR Ebola OR "Zika Virus" OR Pandemic) AND ("Health Personnel" OR "Health Workers") | **331** |
| **ILO** | (COVID OR Coronavirus OR Pandemic) AND ("Health Personnel" OR "Health Workers") | **100** |
