# Supplementary figures and images for: Impacts for health and care workers of Covid-19 and other public health emergencies of international concern: living systematic review, meta-analysis and policy recommendations
Source: Hum Resour Health. 2024 Jan 25;22:10. doi: 10.1186/s12960-024-00892-2 (PMC10809470; doi:10.1186/s12960-024-00892-2)

**Flow for assessing eligibility criteria.**


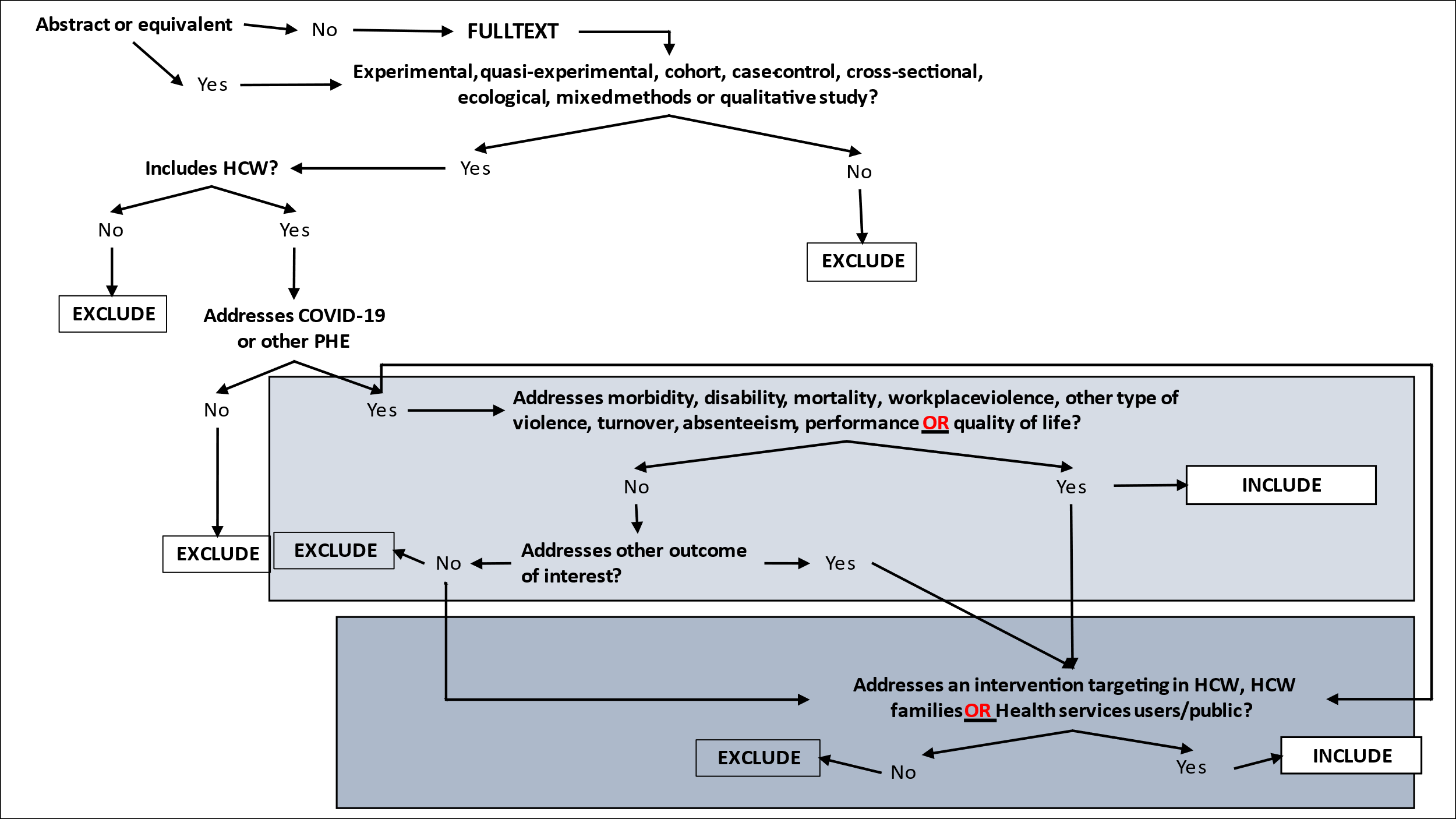

Supplement: Supplementary file 7 — Additional file 7. Included studies. [file 12960_2024_892_MOESM7_ESM.docx]
