# Supplementary material for: Impacts for health and care workers of Covid-19 and other public health emergencies of international concern: living systematic review, meta-analysis and policy recommendations
Source: Hum Resour Health. 2024 Jan 25;22:10. doi: 10.1186/s12960-024-00892-2 (PMC10809470; doi:10.1186/s12960-024-00892-2)
Supplement: Supplementary file 8 — Additional file 8. Excluded studies. [file 12960_2024_892_MOESM8_ESM.docx]

Number and percentage of references assessed, sensitivity, specificity and kappa statistics (interrater agreement) per pair of reviewers

| **Reviewers** | | **IF** | **VM** | **WA** |
| --- | --- | --- | --- | --- |
| **VM** | **N (%) assessed** | 2352 (42.2%) |  |  |
|  | **Sensitivity** | 711/746 (95.3 %) |  |  |
|  | **Specificity** | 1579/1606 (98.3%) |  |  |
|  | **Kappa statistics (p-value)** | 0.939 (p<0.01) |  |  |
| **WA** | **N (%) assessed** | 2300 (41.3%) | 1740 (31.2%) |  |
|  | **Sensitivity** | 599/683 (87.7%) | 553/633 (87.4%) |  |
|  | **Specificity** | 1570/1617 (97.1%) | 1048/1107 (94.7%) |  |
|  | **Kappa statistics (p-value)** | 0.861 (p<0.01) | 0.826 (p<0.01) |  |
| **RL** | **N (%) assessed** |  |  | 560 (10.0%) |
|  | **Sensitivity** |  |  | 207/218 (95.0%) |
|  | **Specificity** |  |  | 285/342 (83.3%) |
|  | **Kappa statistics (p-value)** |  |  | 0.754 (p<0.01) |
