# Supplementary material for: Impacts for health and care workers of Covid-19 and other public health emergencies of international concern: living systematic review, meta-analysis and policy recommendations
Source: Hum Resour Health. 2024 Jan 25;22:10. doi: 10.1186/s12960-024-00892-2 (PMC10809470; doi:10.1186/s12960-024-00892-2)
Supplement: Supplementary file 9 — Additional file 9. Impact of PHEICs on HCW. [file 12960_2024_892_MOESM9_ESM.docx]

Impact of PHEICs on HCW^[[1]](#footnote-1)^

| **Outcome** | **Specificity of determinants during the PHEIC** | | | | | |
| --- | --- | --- | --- | --- | --- | --- |
|  | **PHEIC related** | **Enterprise related** | | **Worker related** | |  |
|  |  | **Policies, programs, practices** | **Conditions of work** | **Professional** | **Socio-demographic, personal traits and individual health** |  |
| **Anxiety (n=518)** | Working in high incidence areas (1,2), in frontline services (2–13), with infected patients (9,14–21)  Fear of being infected and of infecting others (17,22–27)  Previous experience with other PHEICs (28–30)  Stigma and discrimination (31,32) | Organizational support (33,34)  Lack of PPE (6,15,19,35–37)  Insufficient or lack of training and education (13,19,23,33)  Perceived readiness of workplace for PHEICs (24,36,38,39) | Lack of employer support (40)  Long working hours (8,29,41) and workload (42,43)  Fixed term (44), private or public private (39) contracts, independent contractor (45)  Frontline services (2–13)  Working with infected patients (9,14–21)  Telehealth (40) | Education level (1,22,46,47)  Direct contact with patients (48)  Job satisfaction (49)  Years of service/ experience (21,33,38,50,51) | Female (6,16,19,24,30,33,37,42,46,52–69)  Living alone (3,43,48,63,67,70,71)  Having a chronic disease (19,43,69,72)  Pre-existing mental conditions (19,36,49,69,70,73,74)  Younger age (15,19,46,51,56,75–77)  Low social support (20,78)  Ethnicity (36)  Use of social media (79) |  |
| **Depression (n=503)** | Fear of being infected (23,80)  Being infected (20,23,55,59,78,81,82,82–85)  Direct contact with patients (63,84,86,87)  Stigma (36,76,88) | Changes in clinical and operational practices and role (39,89,90)  Preparedness to treat PHEIC patient (23,36,39,91,92)  Lack of PPE (15,93) | Changes in clinical and operational practices and role (39,89,90)  Preparedness to treat PHEIC patient (23,36,39,91,92)  Frontline services (4,31,41,52,56,59,74,88,94–97)  Long working hours (41,88,98,99) and workload (42,93,100)  Violence (23,101)  Direct contact with patients (63,84,86,87)  Lack of employer support (40) | Years of service/ experience (21,50,95,98,102–104)  Public services (55,89) | Female (20,23,30,39,42,54,56,62,68,69,78,82,82,93,95,102,105–108)  Leisure and rest time (23,30)  Use of social media (23,79,108)  Sleep disorders (91,101,109)  Younger age (51,66,69,91,92,106,110–116)  Having a chronic disease (20,42,69,92,101,117)  Pre-existing mental conditions (36,55,69,74,76)  Low social support (77,78,118)  Marital status (66)  Income (3,39,89,100,119)  Substance use (3,92,100) |  |
| **Stress/ distress (n=486)** | Fear of contracting infection (120–126)  Fear of infecting others (127–129)  Infected colleagues (129,130)  Death of a close person due to PHEICs (131)  Screening and Tracing activities (132) | Training of PHEIC (130,133,134) | Changes to clinical and operational practices and role (89,90,124,135–137)  Non supportive leadership (120,138)  Long working hours (69,120,132,139) and workload (140–142)  Access and use PPE (26,88,90,120,122,125,129–131)  Direct contact with patients (20,87,130,140,143–146)  Frontline services (2,4,10,24,93,96,102,135,140,147) | Years of service/ experience (21,91,140)  Job satisfaction (49) | Use of social media (148)  Income (89,128,132,149)  Female (24,55,56,62,69,80,91,93,95,96,105,125,130,134,140,144,150,151)  Younger age (56,62,65,69,93)  Low social support (20,141,152)  Physical illness/ chronic disease (69,126,147)  Pre-existing mental conditions (49,69,74,93) |  |
| **Burnout (n=235)** | Fear of infecting others (153) or being infected (154) |  | Long working hours (154–156) and workload (77,132,157–165)  Access and use of PPE (107,155,161,166–169)  Non supportive leadership (166,170)  Direct contact with patients (107,146,160,171–178)  Frontline services (59,168,179–186)  Work pressure (153) and conflicts (54)  Changes in tasks (54)  Workplace violence (187,188) | Job satisfaction (186,188,189) | Female (53,54,59,107,137,155,173,183,188,190–196)  Younger age (137,167,173,186,189,191,196,197)  Stress (22,176,198–200)  Poor sleep quality (160,201)  Social support (59,77,162)  Depression (22,178,202)  Pre-existing mental conditions (54,203)  Suicide ideation (22) |  |
| **PTSD (n=84)** | Exposure to PHEICs (83)  Fear of infecting others (25)  Being infected (104,204) |  | Frontline services (11,183,205–208)  Access and use PPE (15,93,209,210)  Workload (93,210,211)  Direct contact with patients (15,212)  Team cohesion (76)  Organizational support (213,214) |  | Medical problems (209)  Social support (76,209)  Previous mental health conditions (76,209)  Psychological distress (211,215,216)  Burnout (13,25)  Stress (211,217–219)  Media consumption (76) |  |
| **Suicidal ideation (n=18)** | Exposure to PHEICs (83)  Infected relative/friend (220)  Being hospitalized (221) |  | Organizational support (222) | Years of work (51) | Younger age (220,223)  Living alone (220,221)  Male (220)  Increased alcohol consumption (100,220)  Income (51,100,220)  Previous mental health conditions (220,221,224)  Family and social support (222,225)  Use of media (222)  Psychotropic drug use (100) |  |
| **Other mental health problems (n=51)** | Quarantine (226,227)  PHEIC risk perception (227) |  | Frontline services (184,206,228)  Professional support (227,229)  Long working hours (230)  Job crafting measures (231) | Job contentment (232)  Severe fatigue (233) | Pre-existing mental conditions (233)  Sleep quality (232,233)  Quality of life (234)  Stress (235) |  |
| **Sleep disorders (n=90)** | Working with infected patients (97)  Stigma (36) |  | Long working hours (41)  Frontline services (11,41,51,52,78,236–238) |  | Financial problems (239)  Pre-existing mental conditions (36,74)  Ethnicity (36)  Low social support (77) |  |
| **Headaches (n=11)** |  |  | Use of PPE (240,241)  Use of PPE for prolonged time (242,243) |  | Pre-existing primary headache (240,242,244,245) |  |
| **Skin related morbidity (n=19)** | Working with infected patients (147) |  | Use of PPE for prolonged time (147,246–249)  Frequency of use of PPE (247) | Direct contact with patients (147)  Negative impact on worker performance (250)  Occupation (246) | Female (246)  Previously diagnosed skin diseases and allergies (251) |  |
| **Violence (n=32)** | Lockdown and restriction of movements except for essential workers including HCW (23,252)  Misinformation on HCW (252)  Quarantine/ observation period (252,253)  Lack of public acknowledge (254)  Contact with infected patients/ working PHEIC area (255–258) | Failure to produce identification documents (252)  Lack of public acknowledgement (254) | Unsupportive environment (254,259)  Lack of guidelines or appropriate measures on implementing necessary health protocols (259)  Workload (256) | Risk of transmitting the disease (88,260)  Being known as a HCW (260,261)  Years of service/ experience (256,257,262) | Suffering violence prior to PHEIC (256)  Having family members suffering violence (256) |  |
| **Well-being (n=9)** |  | Psychological support PHEIC (263) | Frontline services (264) |  | Burnout (265)  Resilience (265)  Depression (263)  Stress (263) |  |
| **Quality of life (n=70)** | COVID-19 Anxiety (266,267) | Leadership style (166)  Redeployment(268) | Availability of PPE (167) | Workload (167,234,269,270)  Professional experience (271)  Lack of knowledge on PHEICs (272)  Fatigue (272)  Dual practice(270) | Female (270)  Younger age (167)?  Burnout (166,167,198,273)  Stress (198)  Anxiety (274)  Depression (94,274)  Psychological resilience (274,275)  Health status (276)  Sleep quality (274,276)  Physical activity level (276)  Social support (269) |  |
| **Unplanned absenteeism (n=11)** | Adoption of non-pharmacological measures (277)  Being vaccinated (278)  Fear of the PHEIC (279)  Stigma (279) |  |  | Department (280) | PTSD (279)  Younger age (281) |  |
| **Leaving the occupation (n=20)** | Stigma (282)  Fear (283)  Low trust in PPE (110) |  | Redeployment (282)  Increased working hours (282)  Staffing (157,284)  Private sector (285) |  | Younger age (110,285)  Depression (110)  Stress (110)  Psychiatric symptoms (284)  Burnout (158)  Resilience (286)  Anxiety (284) |  |

References:

1. Guo WP, Min Q, Gu WW, Yu L, Xiao X, Yi WB, et al. Prevalence of mental health problems in frontline healthcare workers after the first outbreak of COVID-19 in China: a cross-sectional study. Health Qual LIFE OUTCOMES. 2021 Mar 22;19(1).

2. Chen Y, Li W. Influencing Factors Associated With Mental Health Outcomes Among Dental Medical Staff in Emergency Exposed to Coronavirus Disease 2019: A Multicenter Cross-Sectional Study in China. Front Psychiatry [Internet]. 2021;12. Available from: ["https://www.embase.com/search/results?subaction=viewrecord&id=L636393479&from=export", "http://dx.doi.org/10.3389/fpsyt.2021.736172"]

3. Baminiwatta A, De Silva S, Hapangama A, Basnayake K, Abayaweera C, Kulasinghe D, et al. Impact of COVID-19 on the mental health of frontline and non-frontline healthcare workers in Sri Lanka. Ceylon Med J. 2021;66(1):16–31.

4. Heidarijamebozorgi M, Jafari H, Sadeghi R, Sheikhbardsiri H, Kargar M, Gharaghani M. The prevalence of depression, anxiety, and stress among nurses during the coronavirus disease 2019: A comparison between nurses in the frontline and the second line of care delivery. Nurs Midwifery Stud [Internet]. 2021;10(3):188–93. Available from: https://www.scopus.com/inward/record.uri?eid=2-s2.0-85111738688&doi=10.4103%2fnms.nms_103_20&partnerID=40&md5=315dac9a79128d6ae37bfde645093c82

5. Saeed BA, Shabila NP, Aziz AJ. Stress and anxiety among physicians during the COVID-19 outbreak in the Iraqi Kurdistan Region: An online survey. PLoS One. 2021;16(6):e0253903.

6. Pouralizadeh M, Bostani Z, Maroufizadeh S, Ghanbari A, Khoshbakht M, Alavi SA, et al. Anxiety and depression and the related factors in nurses of Guilan University of Medical Sciences hospitals during COVID-19: A web-based cross-sectional study. Int J Afr Nurs Sci. 2020;13:100233.

7. Kim SC, Quiban C, Sloan C, Montejano A. Predictors of poor mental health among nurses during COVID-19 pandemic. Nurs Open. 2021;8(2):900–7.

8. Zhou Y, Sun Z, Wang Y, Xing C, Sun L, Shang Z, et al. The prevalence of PTSS under the influence of public health emergencies in last two decades: A systematic review and meta-analysis. Clin Psychol Rev. 2021;83:101938.

9. Franzoi IG, Granieri A, Sauta MD, Agnesone M, Gonella M, Cavallo R, et al. Anxiety, Post-Traumatic Stress, and Burnout in Health Professionals during the COVID-19 Pandemic: Comparing Mental Health Professionals and Other Healthcare Workers. Healthc Basel. 2021;9(6).

10. Antonijevic J, Binic I, Zikic O, Manojlovic S, Tosic-Golubovic S, Popovic N. Mental health of medical personnel during the COVID-19 pandemic. Brain Behav. 2020;10(12):e01881.

11. Cai Z, Cui Q, Liu Z, Li J, Gong X, Liu J, et al. Nurses endured high risks of psychological problems under the epidemic of COVID-19 in a longitudinal study in Wuhan China. J Psychiatr Res. 2020;131:132–7.

12. Salvador AP, Jaloto A, Zuanazzi AC, Gonçalves AP, Machado GM, De Francisco Carvalho L. Impact of anxiety, stress, and burnout symptoms in Brazilian health professionals during the COVID-19 pandemic. Arch Psychiatry Psychother [Internet]. 2021;23(1):7–13. Available from: ["https://www.embase.com/search/results?subaction=viewrecord&id=L2011720985&from=export", "http://dx.doi.org/10.12740/APP/133639"]

13. Caillet A, Coste C, Sanchez R, Allaouchiche B. Psychological Impact of COVID-19 on ICU Caregivers. Anaesth Crit Care Pain Med. 2020 Dec;39(6):717–22.

14. Park C, Hwang JM, Jo S, Bae SJ, Sakong J. COVID-19 Outbreak and Its Association with Healthcare Workers’ Emotional Stress: a Cross-Sectional Study. J Korean Med Sci. 2020;35(41):e372.

15. Arnetz JE, Goetz CM, Sudan S, Arble E, Janisse J, Arnetz BB. Personal Protective Equipment and Mental Health Symptoms Among Nurses During the COVID-19 Pandemic. J Occup Env Med. 2020;62(11):892–7.

16. Fernández-Arana A, Olórtegui-Yzú A, Vega-Dienstmaier JM, Cuesta MJ. Depression and anxiety symptoms and perceived stress in health professionals in the context of COVID-19: Do adverse childhood experiences have a modulating effect? Brain Behav. 2022;12(1):e2452.

17. Ren C, Zhou D, Fan Y, Li B, Zhang W, Shen Y, et al. Prevalence and influencing factors of anxiety and depression symptoms among surgical nurses during COVID-19 pandemic: A large-scale cross-sectional study. Nurs Open. 2022;9(1):752–64.

18. Kumar S, Banerjee A, Ahmad A. A cross-sectional study on mental health and cardiovascular reactivity among fresh resident doctors during COVID19 pandemic in India. Int J Curr Pharm Res [Internet]. 2021;13(3):52–4. Available from: ["https://www.embase.com/search/results?subaction=viewrecord&id=L2012301874&from=export", "http://dx.doi.org/10.22159/ijcpr.2021v13i3.42094"]

19. Cag Y, Erdem H, Gormez A, Ankarali H, Hargreaves S, Ferreira-Coimbra J, et al. Anxiety among front-line health-care workers supporting patients with COVID-19: A global survey. Gen Hosp Psychiatry. 2021;68:90–6.

20. Asnakew S, Amha H, Kassew T. Mental Health Adverse Effects of COVID-19 Pandemic on Health Care Workers in North West Ethiopia: A Multicenter Cross-Sectional Study. Neuropsychiatr Treat. 2021;17:1375–84.

21. Nadeem F, Sadiq A, Raziq A, Iqbal Q, Haider S, Saleem F, et al. Depression, Anxiety, and Stress Among Nurses During the COVID-19 Wave III: Results of a Cross-Sectional Assessment. J Multidiscip Heal. 2021;14:3093–101.

22. Pappa S, Athanasiou N, Sakkas N, Patrinos S, Sakka E, Barmparessou Z, et al. From Recession to Depression? Prevalence and Correlates of Depression, Anxiety, Traumatic Stress and Burnout in Healthcare Workers during the COVID-19 Pandemic in Greece: A Multi-Center, Cross-Sectional Study. Int J Environ Res Public Health. 2021 Mar;18(5).

23. Hasan MT, Sahadat H, Farhana S, Afifa A, Abid Hasan K, Kamrun Nahar K, et al. Prevalence of anxiety and depressive symptoms among physicians during the COVID-19 pandemic in Bangladesh: a cross-sectional study. 2020; Available from: https://medrxiv.org/cgi/content/short/2020.12.08.20245829

24. Saddik B, Elbarazi I, Temsah MH, Saheb Sharif-Askari F, Kheder W, Hussein A, et al. Psychological Distress and Anxiety Levels Among Health Care Workers at the Height of the COVID-19 Pandemic in the United Arab Emirates. Int J Public Health. 2021;66:1604369–1604369.

25. Mosheva M, Gross R, Hertz-Palmor N, Hasson-Ohayon I, Kaplan R, Cleper R, et al. The association between witnessing patient death and mental health outcomes in frontline COVID-19 healthcare workers. Depress ANXIETY. 2021 Apr;38(4):468–79.

26. Sampaio F, Sequeira C, Teixeira L. Nurses’ Mental Health During the Covid-19 Outbreak: A Cross-Sectional Study. J Occup Env Med. 2020;62(10):783–7.

27. Morawa E, Schug C, Geiser F, Beschoner P, Jerg-Bretzke L, Albus C, et al. Psychosocial burden and working conditions during the COVID-19 pandemic in Germany: The VOICE survey among 3678 health care workers in hospitals. J Psychosom Res. 2021;144:110415.

28. Styra R, Hawryluck L, Mc Geer A, Dimas M, Sheen J, Giacobbe P, et al. Surviving SARS and living through COVID-19: Healthcare worker mental health outcomes and insights for coping. PloS One. 2021;16(11):e0258893–e0258893.

29. Pang Y, Fang H, Li L, Chen M, Chen Y. Predictive factors of anxiety and depression among nurses fighting coronavirus disease 2019 in China. Int J Ment Health Nurs. 2021;30(2):524–32.

30. Matsumoto Y, Fujino J, Shiwaku H, Miyajima M, Doi S, Hirai N, et al. Factors affecting mental illness and social stress in hospital workers treating COVID-19: Paradoxical distress during pandemic era. J Psychiatr Res. 2021;137:298–302.

31. Chatterjee SS, Bhattacharyya R, Bhattacharyya S, Gupta S, Das S, Banerjee BB. Attitude, practice, behavior, and mental health impact of COVID-19 on doctors. Indian J Psychiatry. 2020;62(3):257–65.

32. Monterrosa-Castro A, Redondo-Mendoza V, Mercado-Lara M. Psychosocial factors associated with symptoms of generalized anxiety disorder in general practitioners during the COVID-19 pandemic. J Investig Med. 2020;68(7):1228–34.

33. Baraka AAE, Ramadan FH, Hassan EA. Predictors of critical care nurses’ stress, anxiety, and depression in response to COVID-19 pandemic. Nurs Crit Care. 2021;

34. Labrague LJ, De Los Santos JAA. COVID-19 anxiety among front-line nurses: Predictive role of organisational support, personal resilience and social support. J Nurs Manag. 2020;28(7):1653–61.

35. Dong H shuo, Gao J jing, Dong YX, Han C xia, Sun L. Prevalence of insomnia and anxiety among healthcare workers during the COVID-19 pandemic in Jilin Province. Braz J Med Biol Res. 2021;54(9).

36. Khanal P, Devkota N, Dahal M, Paudel K, Joshi D. Mental health impacts among health workers during COVID-19 in a low resource setting: a cross-sectional survey from Nepal. Glob Health. 2020;16(1):89.

37. Pazmiño Erazo EE, Alvear Velásquez MJ, Saltos Chávez IG, Pazmiño Pullas DE. Factors Associated With Psychiatric Adverse Effects in Healthcare Personnel During the COVID-19 Pandemic in Ecuador. Rev Colomb Psiquiatr [Internet]. 2021;50(3):166–75. Available from: ["https://www.embase.com/search/results?subaction=viewrecord&id=L2011342282&from=export", "http://dx.doi.org/10.1016/j.rcp.2020.12.007"]

38. Cho M, Kim O, Pang Y, Kim B, Jeong H, Lee J, et al. Factors affecting frontline Korean nurses’ mental health during the COVID-19 pandemic. Int Nurs Rev. 2021;68(2):256–65.

39. Santos KMR dos, Galvão MHR, Gomes SM, Souza TA de, Medeiros A de A, Barbosa IR. Depressão e ansiedade em profissionais de enfermagem durante a pandemia da covid-19. Esc Anna Nery Rev Enferm [Internet]. 2021;25:e20200370–e20200370. Available from: https://www.scielo.br/scielo.php?script=sci_arttext&pid=S1414-81452021000500201

40. Gainer DM, Nahhas RW, Bhatt NV, McCormack J, McMerrill A, Sultan H. Factors associated with depression and anxiety symptoms among U.S. physicians during the first phase of the COVID-19 pandemic. Int J Health Promot Educ [Internet]. 2022; Available from: ["https://www.embase.com/search/results?subaction=viewrecord&id=L2014975059&from=export", "http://dx.doi.org/10.1080/14635240.2022.2028264"]

41. Elmahdy MA, Shebl EM. Mental health outcomes among health care workers exposed to covid-19 pandemic, qalyoubia governorate: Cross-sectional survey. Egypt J Hosp Med [Internet]. 2021;84(1):1945–54. Available from: https://www.scopus.com/inward/record.uri?eid=2-s2.0-85119611847&doi=10.21608%2fEJHM.2021.178615&partnerID=40&md5=e8ff1115c97841a30a4866b6179be877

42. Arslan HN, Karabekiroglu A, Terzi O, Dundar C. The effects of the COVID-19 outbreak on physicians’ psychological resilience levels. Postgrad Med. 2021;133(2):223–30.

43. Koksal E, Dost B, Terzi Ö, Ustun YB, Özdin S, Bilgin S. Evaluation of Depression and Anxiety Levels and Related Factors Among Operating Theater Workers During the Novel Coronavirus (COVID-19) Pandemic. J Perianesth Nurs. 2020;35(5):472–7.

44. Chen X, Arber A, Gao J, Zhang L, Ji M, Wang D, et al. The mental health status among nurses from low-risk areas under normalized COVID-19 pandemic prevention and control in China: A cross-sectional study. Int J Ment Health Nurs. 2021;30(4):975–87.

45. Paolocci G, Bussotti P, Pompili C, Muzi G, Gambelunghe A, Dell’Omo M, et al. Impact of the COVID-19 pandemic and work-related stress in Umbrian healthcare workers during Phase 1 in Italy. Med Lav. 2021;112(6):486–95.

46. Mi T, Yang X, Sun S, Li X, Tam CC, Zhou Y, et al. Mental Health Problems of HIV Healthcare Providers During the COVID-19 Pandemic: The Interactive Effects of Stressors and Coping. AIDS Behav. 2021 Jan;25(1):18–27.

47. Sharma SK, Mudgal SK, Thakur K, Parihar A, Chundawat DS, Joshi J. Anxiety, depression and quality of life (QOL) related to COVID-19 among frontline health care professionals: A multicentric cross-sectional survey. J Fam Med Prim Care. 2021;10(3):1383–9.

48. Fatemeh T, Leila H, Mahmood M, Mehran Z, Hassan T, Azadeh Fathi D, et al. Anxiety and Depression in Health Workers and General Population During COVID-19 Epidemic in IRAN: A Web-Based Cross-Sectional Study. 2020; Available from: https://medrxiv.org/cgi/content/short/2020.05.05.20089292

49. Da Rosa P, Brown R, Pravecek B, Carotta C, Garcia AS, Carson P, et al. Factors associated with nurses emotional distress during the COVID-19 pandemic. Appl Nurs Res. 2021;62:151502.

50. Gupta S, Prasad AS, Dixit PK, Padmakumari P, Abhisheka K. Survey of prevalence of anxiety and depressive symptoms among 1124 healthcare workers during the coronavirus disease 2019 pandemic across India. Med J Armed Forces India [Internet]. 2021;77:S404–12. Available from: ["https://www.embase.com/search/results?subaction=viewrecord&id=L2007661920&from=export", "http://dx.doi.org/10.1016/j.mjafi.2020.07.006"]

51. Zhou Y, Wang W, Sun Y, Qian W, Liu Z, Wang R, et al. The prevalence and risk factors of psychological disturbances of frontline medical staff in china under the COVID-19 epidemic: Workload should be concerned. J Affect Disord. 2020;277:510–4.

52. Lai J, Ma S, Wang Y, Cai Z, Hu J, Wei N, et al. Factors Associated With Mental Health Outcomes Among Health Care Workers Exposed to Coronavirus Disease 2019. JAMA Netw Open. 2020 Mar 2;3(3):e203976.

53. Civantos AM, Byrnes Y, Chang C, Prasad A, Chorath K, Poonia SK, et al. Mental health among otolaryngology resident and attending physicians during the COVID-19 pandemic: National study. Head Neck. 2020;42(7):1597–609.

54. Lasalvia A, Bodini L, Amaddeo F, Porru S, Carta A, Poli R, et al. The Sustained Psychological Impact of the COVID-19 Pandemic on Health Care Workers One Year after the Outbreak-A Repeated Cross-Sectional Survey in a Tertiary Hospital of North-East Italy. Int J Env Res Public Health. 2021;18(24).

55. Ghaleb Y, Lami F, Al Nsour M, Rashak HA, Samy S, Khader YS, et al. Mental health impacts of COVID-19 on healthcare workers in the Eastern Mediterranean Region: a multi-country study. J Public Health Oxf. 2021;43:iii34–42.

56. Elkholy H, Tawfik F, Ibrahim I, Salah El-Din W, Sabry M, Mohammed S, et al. Mental health of frontline healthcare workers exposed to COVID-19 in Egypt: A call for action. Int J Soc Psychiatry. 2021;67(5):522–31.

57. Luceño-Moreno L, Talavera-Velasco B, García-Albuerne Y, Martín-García J. Symptoms of posttraumatic stress, anxiety, depression, levels of resilience and burnout in Spanish health personnel during the COVID-19 pandemic. Int J Environ Res Public Health. 2020;17(15):5514.

58. Elawady MA, Abd-Elraouf MSED. Effect of coronavirus disease 2019 pandemic on mental health among health care workers and others. Egypt J Hosp Med [Internet]. 2021;85(1):3306–12. Available from: https://www.scopus.com/inward/record.uri?eid=2-s2.0-85119601679&doi=10.21608%2fEJHM.2021.197373&partnerID=40&md5=4d1f56c6d658deddd823d360c5936071

59. Karacan FA, Yilmaz S, Kirpinar I. Psychosocial Adjustment of Healthcare Professionals During the COVID-19 Pandemic: Resident Doctors, Nurses, and Caregivers Need Extra Attention. Med J Bakirkoy [Internet]. 2021;17(4):375–85. Available from: ["https://www.embase.com/search/results?subaction=viewrecord&id=L2016404761&from=export", "http://dx.doi.org/10.4274/BMJ.galenos.2021.46338"]

60. Kurt O, Deveci SE, Oguzoncul AF. Levels of anxiety and depression related to covid-19 among physicians: An online cross-sectional study from turkey. Ann Clin Anal Med [Internet]. 2020;11:S288–93. Available from: ["https://www.embase.com/search/results?subaction=viewrecord&id=L2004647153&from=export", "http://dx.doi.org/10.4328/ACAM.20206"]

61. Xu L, You D, Li C, Zhang X, Yang R, Kang C, et al. Two-stage mental health survey of first-line medical staff after ending COVID-19 epidemic assistance and isolation. Eur Arch Psychiatry Clin Neurosci. 2022;272(1):81–93.

62. Arafa A, Mohammed Z, Mahmoud O, Elshazley M, Ewis A. Depressed, anxious, and stressed: What have healthcare workers on the frontlines in Egypt and Saudi Arabia experienced during the COVID-19 pandemic? J Affect Disord. 2021;278:365–71.

63. Nayak BS, Sahu PK, Ramsaroop K, Maharaj S, Mootoo W, Khan S, et al. Prevalence and factors associated with depression, anxiety and stress among healthcare workers of Trinidad and Tobago during COVID-19 pandemic: a cross-sectional study. BMJ Open. 2021;11(4):e044397.

64. Onchonga D, Ngetich E, Makunda W, Wainaina P, Wangeshi D, Viktoria P. Anxiety and depression due to 2019 SARS-CoV-2 among frontier healthcare workers in Kenya. Heliyon. 2021;7(3):e06351.

65. Elsaie ML, Hasan MS, Zaky MS, Hussein SM, Kadah AS, Omar AM. Implication of COVID-19 on the mental health of Egyptian dermatologists: A cross-sectional study. J Cosmet Dermatol. 2021;20(10):3066–73.

66. He L, Wang J, Zhang L, Wang F, Dong W, Zhao W. Risk Factors for Anxiety and Depressive Symptoms in Doctors During the Coronavirus Disease 2019 Pandemic. Front Psychiatry [Internet]. 2021;12. Available from: ["https://www.embase.com/search/results?subaction=viewrecord&id=L635402189&from=export", "http://dx.doi.org/10.3389/fpsyt.2021.687440"]

67. Kirk AHP, Chong SL, Kam KQ, Huang W, Ang LSL, Lee JH, et al. Psychosocial impact of the COVID-19 pandemic on paediatric healthcare workers. Ann Acad Med Singap. 2021;50(3):203–11.

68. Liu S, Yang L, Zhang C, Xu Y, Cai L, Ma S, et al. Gender differences in mental health problems of healthcare workers during the coronavirus disease 2019 outbreak. J Psychiatr Res. 2021;137:393–400.

69. Azizi M, Kamali M, Moosazadeh M, Aarabi M, Ghasemian R, Hasannezhad Reskati M, et al. Assessing mental health status among Iranian healthcare workers in times of the COVID-19 pandemic: A web-based cross-sectional study. Brain Behav. 2021;11(8):e2304.

70. Debski M, Abdelaziz HK, Sanderson J, Wild S, Assaf O, Wiper A, et al. Mental Health Outcomes Among British Healthcare Workers-Lessons From the First Wave of the Covid-19 Pandemic. J Occup Environ Med. 2021 Aug;63(8):E549–55.

71. Sheikhbardsiri H, Doustmohammadi MM, Afshar PJ, Heidarijamebozorgi M, Khankeh H, Beyramijam M. Anxiety, stress and depression levels among nurses of educational hospitals in Iran: Time of performing nursing care for suspected and confirmed COVID-19 patients. J Educ Health Promot. 2021;10:447.

72. Si MY, Su XY, Jiang Y, Wang WJ, Gu XF, Ma L, et al. Psychological impact of COVID-19 on medical care workers in China. Infect Poverty. 2020;9(1):113.

73. Zhou P, Du N, Diao D, OuYang Y, Kankanam Pathiranage HS. Investigation on the Influencing Factors of Mental Health of Healthcare Workers for Aid in Hubei during the Outbreak of COVID-19. Ann Work Expo Health. 2021;65(7):833–42.

74. Şahin MK, Aker S, Şahin G, Karabekiroğlu A. Prevalence of Depression, Anxiety, Distress and Insomnia and Related Factors in Healthcare Workers During COVID-19 Pandemic in Turkey. J Community Health. 2020;45(6):1168–77.

75. Salehiniya H, Abbaszadeh H. Prevalence of corona-associated anxiety and mental health disorder among dentists during the COVID-19 pandemic. Neuropsychopharmacol Rep. 2021;41(2):223–9.

76. Hennein R, Mew EJ, Lowe SR. Socio-ecological predictors of mental health outcomes among healthcare workers during the COVID-19 pandemic in the United States. PLOS ONE. 2021 Feb 5;16(2).

77. Tiete J, Guatteri M, Lachaux A, Matossian A, Hougardy JM, Loas G, et al. Mental Health Outcomes in Healthcare Workers in COVID-19 and Non-COVID-19 Care Units: A Cross-Sectional Survey in Belgium. Front Psychol. 2020;11:612241.

78. Bhattacharya PK, Prakash J. Impact of COVID-19 pandemic on the emotional well-being of healthcare workers: A multinational cross-sectional survey. Indian J Crit Care Med [Internet]. 2021;25(5):479–81. Available from: ["https://www.embase.com/search/results?subaction=viewrecord&id=L2007130772&from=export", "http://dx.doi.org/10.5005/jp-journals-10071-23833"]

79. He Q, Fan B, Xie B, Liao Y, Han X, Chen Y, et al. Mental health conditions among the general population, healthcare workers and quarantined population during the coronavirus disease 2019 (COVID-19) pandemic. Psychol Health Med. 2022;27(1):186–98.

80. Gorini A, Fiabane E, Sommaruga M, Barbieri S, Sottotetti F, La Rovere MT, et al. Mental health and risk perception among Italian healthcare workers during the second month of the Covid-19 pandemic. Arch Psychiatr Nurs. 2020 Dec;34(6):537–44.

81. Lee AM, Wong JG, McAlonan GM, Cheung V, Cheung C, Sham PC, et al. Stress and psychological distress among SARS survivors 1 year after the outbreak. Can J Psychiatry. 2007;52(4):233–40.

82. Motahedi S, Aghdam NF, Khajeh M, Baha R, Aliyari R, Bagheri H, et al. Anxiety and depression among healthcare workers during COVID-19 pandemic: A cross-sectional study. Heliyon. 2021;7(12):e08570.

83. Amsalem D, Lazarov A, Markowitz JC, Naiman A, Smith TE, Dixon LB, et al. Psychiatric symptoms and moral injury among US healthcare workers in the COVID-19 era. BMC PSYCHIATRY. 2021 Nov 5;21(1).

84. Hayat K, Arshed M, Fiaz I, Afreen U, Khan FU, Khan TA, et al. Impact of COVID-19 on the Mental Health of Healthcare Workers: A Cross-Sectional Study From Pakistan. Front PUBLIC Health. 2021 Apr 26;9.

85. Prekazi L, Hajrullahu V, Bahtiri S, Kryeziu B, Hyseni B, Taganoviq B, et al. The Impact of Coping Skills in Post-traumatic Growth of Healthcare Providers: When Mental Health Is Deteriorating Due to COVID-19 Pandemic. Front Psychol. 2021;12:791568.

86. Alsairafi Z, Naser AY, Alsaleh FM, Awad A, Jalal Z. Mental Health Status of Healthcare Professionals and Students of Health Sciences Faculties in Kuwait during the COVID-19 Pandemic. Int J Env Res Public Health. 2021;18(4).

87. Dziedzic B, Kobos E, Sienkiewicz Z, Idzik A. Mental Health of Nurses during the Fourth Wave of the COVID-19 Pandemic in Poland. Int J Env Res Public Health. 2022;19(3).

88. Han S, Choi S, Cho SH, Lee J, Yun JY. Associations between the working experiences at frontline of COVID-19 pandemic and mental health of Korean public health doctors. BMC PSYCHIATRY. 2021 Jun 9;21(1).

89. Lum A, Goh YL, Wong KS, Seah J, Teo G, Ng JQ, et al. Impact of COVID-19 on the mental health of Singaporean GPs: a cross-sectional study. BJGP Open. 2021;5(4).

90. Mediavilla R, Fernandez-Jimenez E, Martinez-Ales G, Moreno-Kustner B, Martinez-Morata I, Jaramillo F, et al. Role of access to personal protective equipment, treatment prioritization decisions, and changes in job functions on health workers’ mental health outcomes during the initial outbreak of the COVID-19 pandemic. J Affect Disord. 2021 Aug 12;295:405–9.

91. Youssef N, Mostafa A, Ezzat R, Yosef M, Kassas ME. Mental health status of health-care professionals working in quarantine and non-quarantine egyptian hospitals during the covid-19 pandemic. East Mediterr Health J [Internet]. 2020;26(10):1155–64. Available from: https://www.scopus.com/inward/record.uri?eid=2-s2.0-85092800474&doi=10.26719%2femhj.20.116&partnerID=40&md5=8c73e19a2bfebcc41aebacdc06de8643

92. Wayessa ZJ, Melesse GT, Amaje Hadona E, Wako WG. Prevalence of depressive symptoms due to COVID-19 and associated factors among healthcare workers in Southern Ethiopia. SAGE Open Med [Internet]. 2021;9. Available from: ["https://www.embase.com/search/results?subaction=viewrecord&id=L2013151678&from=export", "http://dx.doi.org/10.1177/20503121211032810"]

93. Gilleen J, Santaolalla A, Valdearenas L, Salice C, Fusté M. Impact of the COVID-19 pandemic on the mental health and well-being of UK healthcare workers. BJPsych Open. 2021;7(3):e88.

94. An Y, Yang Y, Wang A, Li Y, Zhang Q, Cheung T, et al. Prevalence of depression and its impact on quality of life among frontline nurses in emergency departments during the COVID-19 outbreak. J Affect Disord. 2020 Nov 1;276:312–5.

95. Jemal K, Deriba BS, Geleta TA. Psychological Distress, Early Behavioral Response, and Perception Toward the COVID-19 Pandemic Among Health Care Workers in North Shoa Zone, Oromiya Region. Front Psychiatry. 2021;12:628898.

96. Jemal K, Deriba BS, Geleta TA, Tesema M, Awol M, Mengistu E, et al. Self-Reported Symptoms of Depression, Anxiety, and Stress Among Healthcare Workers in Ethiopia During the COVID-19 Pandemic: A Cross-Sectional Study. Neuropsychiatr Treat. 2021;17:1363–73.

97. Su TP, Lien TC, Yang CY, Su YL, Wang JH, Tsai SL, et al. Prevalence of psychiatric morbidity and psychological adaptation of the nurses in a structured SARS caring unit during outbreak: a prospective and periodic assessment study in Taiwan. J Psychiatr Res. 2007;41(1):119–30.

98. Zakeri MA, Rahiminezhad E, Salehi F, Ganjeh H, Dehghan M. Burnout, Anxiety, Stress, and Depression Among Iranian Nurses: Before and During the First Wave of the COVID-19 Pandemic. Front Psychol. 2021;12:789737.

99. Valaine L, Ancāne G, Utināns A, Briģis Ģ. Mental Health and Associated Demographic and Occupational Factors among Health Care Workers during the COVID-19 Pandemic in Latvia. Med Kaunas. 2021;57(12).

100. Oliveira MM, Treichel C, Bakolis I, Alves PF, Coimbra VCC, Cavada GP, et al. Mental health of nursing professionals during the COVID-19 pandemic: a cross-sectional study. Rev Saude Publica. 2022;56:8.

101. Dehon E, Clair E. Prevalence and predictors of distress among emergency medicine physicians during COVID-19. Acad Emerg Med [Internet]. 2021;28:S147. Available from: ["https://www.embase.com/search/results?subaction=viewrecord&id=L635077390&from=export", "http://dx.doi.org/10.1111/acem.14249"]

102. Napoli G. Stress and depressive symptoms among Italian mental health nurses during the COVID-19 pandemic, a cross-sectional study. Arch Psychiatr Nurs. 2022;36:41–7.

103. Htay MNN, Marzo RR, AlRifai A, Kamberi F, El-Abasiri RA, Nyamache JM, et al. Immediate impact of COVID-19 on mental health and its associated factors among healthcare workers: A global perspective across 31 countries. J Glob Health. 2020 Dec;10(2).

104. Villalba-Arias J, Estigarribia G, Bogado JA, Méndez J, Toledo S, Barrios I, et al. Mental health issues and psychological risk factors among Paraguayan healthcare workers during the COVID-19 pandemic. J Ment Health. 2021;1–8.

105. Zhang C, Peng D, Lv L, Zhuo K, Yu K, Shen T, et al. Individual perceived stress mediates psychological distress in medical workers during covid-19 epidemic outbreak in Wuhan. Neuropsychiatr Dis Treat [Internet]. 2020;16:2529–37. Available from: ["https://www.embase.com/search/results?subaction=viewrecord&id=L2005410644&from=export", "http://dx.doi.org/10.2147/NDT.S266151"]

106. Rodolfo R, Valentina S, Francesca P, Giorgio Di L, Antinisca Di M, Alberto S, et al. Mental health outcomes among front and second line health workers associated with the COVID-19 pandemic in Italy. 2020; Available from: https://medrxiv.org/cgi/content/short/2020.04.16.20067801

107. Coleman JR, Abdelsattar JM, Glocker RJ. COVID-19 Pandemic and the Lived Experience of Surgical Residents, Fellows, and Early-Career Surgeons in the American College of Surgeons. J Am Coll Surg. 2021;232(2):119-135.e20.

108. Lu P, Li X, Lu L, Zhang Y. The psychological states of people after Wuhan eased the lockdown. PLOS ONE. 2020 Nov 12;15(11).

109. Chen X, Liu P, Lei GF, Tong L, Wang H, Zhang XQ. Sleep Quality and the Depression-Anxiety-Stress State of Frontline Nurses Who Perform Nucleic Acid Sample Collection During COVID-19: A Cross-Sectional Study. Psychol Res Behav Manag. 2021;14:1889–900.

110. Norkiene I, Jovarauskaite L, Kvedaraite M, Uppal E, Phull MK, Chander H, et al. “Should I Stay, or Should I Go?” Psychological Distress Predicts Career Change Ideation among Intensive Care Staff in Lithuania and the UK Amid COVID-19 Pandemic. Int J Environ Res Public Health. 2021 Mar;18(5).

111. Küppers L, Amarell N, Thielmann A, Filbert AL, Schmidt M, Kasten S, et al. High psychological burden of young family physicians early in the COVID-19 pandemic: Results from an exploratory survey. Z Allgemeinmed [Internet]. 2021;97(7):325–31. Available from: ["https://www.embase.com/search/results?subaction=viewrecord&id=L2013578774&from=export", "http://dx.doi.org/10.3238/zfa.2021.0325-0331"]

112. Arshad MS, Hussain I, Nafees M, Majeed A, Imran I, Saeed H, et al. Assessing the Impact of COVID-19 on the Mental Health of Healthcare Workers in Three Metropolitan Cities of Pakistan. Psychol Res Behav Manag. 2020;13:1047–55.

113. Awano N, Oyama N, Akiyama K, Inomata M, Kuse N, Tone M, et al. Anxiety, Depression, and Resilience of Healthcare Workers in Japan During the Coronavirus Disease 2019 Outbreak. Intern Med. 2020;59(21):2693–9.

114. Doan QH, Tran NN, Than MH, Nguyen HT, Bui VS, Nguyen DH, et al. Depression, Anxiety and Associated Factors among Frontline Hospital Healthcare Workers in the Fourth Wave of COVID-19: Empirical Findings from Vietnam. Trop Med Infect Dis. 2021;7(1).

115. Xing LQ, Xu ML, Sun J, Wang QX, Ge DD, Jiang MM, et al. Anxiety and depression in frontline health care workers during the outbreak of Covid-19. Int J Soc Psychiatry. 2021;67(6):656–63.

116. Ghio L, Patti S, Piccinini G, Modafferi C, Lusetti E, Mazzella M, et al. Anxiety, Depression and Risk of Post-Traumatic Stress Disorder in Health Workers: The Relationship with Burnout during COVID-19 Pandemic in Italy. Int J Environ Res Public Health. 2021 Sep;18(18).

117. Chui PL, Chong MC, Abdullah KL, Ramoo V, Tang LY, Lee WL, et al. The COVID-19 Global Pandemic and Its Impact on the Mental Health of Nurses in Malaysia. Healthc Basel. 2021;9(10).

118. Tatsuno J, Unoki T, Sakuramoto H, Hamamoto M. Effects of social support on mental health for critical care nurses during the coronavirus disease 2019 (COVID-19) pandemic in Japan: A web-based cross-sectional study. Acute Med Surg. 2021;8(1):e645.

119. Mekhemar M, Attia S, Dörfer C, Conrad J. Dental Nurses’ Mental Health in Germany: A Nationwide Survey during the COVID-19 Pandemic. Int J Env Res Public Health. 2021;18(15).

120. Ness MM, Saylor J, Di Fusco LA, Evans K. Healthcare providers’ challenges during the coronavirus disease (COVID-19) pandemic: A qualitative approach. Nurs Health Sci. 2021 Jun;23(2):389–97.

121. George CE, Inbaraj LR, Rajukutty S, de Witte LP. Challenges, experience and coping of health professionals in delivering healthcare in an urban slum in India during the first 40 days of COVID-19 crisis: a mixed method study. BMJ Open. 2020;10(11):e042171.

122. Zhang Y, Wang C, Pan W, Zheng J, Gao J, Huang X, et al. Stress, Burnout, and Coping Strategies of Frontline Nurses During the COVID-19 Epidemic in Wuhan and Shanghai, China. Front Psychiatry [Internet]. 2020;11. Available from: ["https://www.embase.com/search/results?subaction=viewrecord&id=L633333831&from=export", "http://dx.doi.org/10.3389/fpsyt.2020.565520"]

123. Asaoka H, Sasaki N, Kuroda R, Tsuno K, Kawakami N. Workplace Bullying and Patient Aggression Related to COVID-19 and its Association with Psychological Distress among Health Care Professionals during the COVID-19 Pandemic in Japan. Tohoku J Exp Med. 2021;255(4):283–9.

124. Galletta M, Piras I, Finco G, Meloni F, D’Aloja E, Contu P, et al. Worries, Preparedness, and Perceived Impact of Covid-19 Pandemic on Nurses’ Mental Health. Front Public Health. 2021;9:566700.

125. Gonzalo RM, Ana RG, Patricia CA, Laura AL, Nathalia GT, Luis C, et al. Short-term emotional impact of COVID-19 pandemic on Spaniard health workers. J Affect Disord. 2021;278:390–4.

126. Shacham M, Hamama-Raz Y, Kolerman R, Mijiritsky O, Ben-Ezra M, Mijiritsky E. COVID-19 Factors and Psychological Factors Associated with Elevated Psychological Distress among Dentists and Dental Hygienists in Israel. Int J Environ Res Public Health. 2020 Apr;17(8).

127. Mazza C, Colasanti M, Ricci E, Di Giandomenico S, Marchetti D, Fontanesi L, et al. The covid-19 outbreak and psychological distress in healthcare workers: The role of personality traits, attachment styles, and sociodemographic factors. Sustain Switz [Internet]. 2021;13(9). Available from: https://www.scopus.com/inward/record.uri?eid=2-s2.0-85105803461&doi=10.3390%2fsu13094992&partnerID=40&md5=2edd7ad38b341f68e448aaff0e612837

128. Costa D de S, Paula JJ de, Serpa AL de O, Diaz AP, Rocha MCM da, Pinto AL de CB, et al. Preditores de sofrimento psicológico e prevalência de transtornos mentais autodeclarados em profissionais de saúde e na população em geral durante a pandemia de Covid-19 no Brasil. Rev Bras Psicoter Online [Internet]. 2021;23(3):47–70. Available from: https://rbp.celg.org.br/audiencia_pdf.asp?aid2=400&nomeArquivo=v23n3a06.pdf

129. Cebrián-Cuenca A, Mira JJ, Caride-Miana E, Fernández-Jiménez A, Orozco-Beltrán D. Sources of psychological distress among primary care physicians during the COVID-19 pandemic’s first wave in Spain: a cross-sectional study. Prim Health Care Res Dev. 2021;22:e55.

130. Badru OA, Oloko KO, Hassan AO, Yusuf OB, Abdur-Razaq UA, Yakub S. Prevalence and correlates of psychological distress amongst healthcare workers during the COVID-19 pandemic: An online survey. Afr J Psychiatr. 2021;27:1617.

131. Erquicia J, Valls L, Barja A, Gil S, Miquel J, Leal-Blanquet J, et al. Emotional impact of the Covid-19 pandemic on healthcare workers in one of the most important infection outbreaks in Europe. Med Clin (Barc). 2020 Nov 27;155(10):434–40.

132. Menon GR, Yadav J, Aggarwal S, Singh R, Kaur S, Chakma T, et al. Psychological distress and burnout among healthcare worker during COVID-19 pandemic in India-A cross-sectional study. PLoS One. 2022;17(3):e0264956.

133. Andlib S, Inayat S, Azhar K, Aziz F. Burnout and psychological distress among Pakistani nurses providing care to COVID-19 patients: A cross-sectional study. Int Nurs Rev. 2022;

134. Jang OJ, Chung YI, Lee JW, Kim HC, Seo JS. Emotional Distress of the COVID-19 Cluster Infection on Health Care Workers Working at a National Hospital in Korea. J Korean Med Sci. 2021;36(47):e324.

135. Al-Mansour K, Alfuzan A, Alsarheed D, Alenezi M, Abogazalah F. Work-Related Challenges among Primary Health Centers Workers during COVID-19 in Saudi Arabia. Int J Env Res Public Health. 2021;18(4).

136. Alyahya SA, Al-Mansour KA, Alkohaiz MA, Almalki MA. Association between role conflict and ambiguity and stress among nurses in primary health care centers in Saudi Arabia during the coronavirus disease 2019 pandemic: A cross-sectional study. Med Baltim. 2021;100(37):e27294.

137. Gramaglia C, Marangon D, Azzolina D, Guerriero C, Lorenzini L, Probo M, et al. The Mental Health Impact of 2019-nCOVID on Healthcare Workers From North-Eastern Piedmont, Italy. Focus on Burnout. Front PUBLIC Health. 2021 May 11;9.

138. Zaghini F, Fiorini J, Livigni L, Carrabs G, Sili A. A mixed methods study of an organization’s approach to the COVID-19 health care crisis. Nurs OUTLOOK. 2021 Oct;69(5):793–804.

139. Mo Y, Deng L, Zhang L, Lang Q, Liao C, Wang N, et al. Work stress among Chinese nurses to support Wuhan in fighting against COVID-19 epidemic. J Nurs Manag. 2020;28(5):1002–9.

140. Linzer M, Stillman M, Brown R, Taylor S, Nankivil N, Poplau S, et al. Preliminary Report: US Physician Stress During the Early Days of the COVID-19 Pandemic. Mayo Clin Proc Innov Qual Outcomes [Internet]. 2021;5(1):127–36. Available from: ["https://www.embase.com/search/results?subaction=viewrecord&id=L2011143601&from=export", "http://dx.doi.org/10.1016/j.mayocpiqo.2021.01.005"]

141. Dutour M, Kirchhoff A, Janssen C, Meleze S, Chevalier H, Levy-Amon S, et al. Family medicine practitioners’ stress during the COVID-19 pandemic: a cross-sectional survey. BMC Fam Pr. 2021;22(1):36.

142. Chen HM, Liu CC, Yang SY, Wang YR, Hsieh PL. Factors Related to Care Competence, Workplace Stress, and Intention to Stay among Novice Nurses during the Coronavirus Disease (COVID-19) Pandemic. Int J Env Res Public Health. 2021;18(4).

143. Al-Amer RM, Malak MZ, Aburumman G, Darwish M, Nassar MS, Randall S. Prevalence and predictors of depression, anxiety, and stress among Jordanian nurses during the coronavirus disease 2019 pandemic. Int J Ment Health [Internet]. 2021; Available from: https://www.scopus.com/inward/record.uri?eid=2-s2.0-85105985483&doi=10.1080%2f00207411.2021.1916701&partnerID=40&md5=8a0967515da37174dd48a4ed41e234fd

144. Ayalew M, Deribe B, Abraham Y, Reta Y, Tadesse F, Defar S, et al. Prevalence and determinant factors of mental health problems among healthcare professionals during COVID-19 pandemic in southern Ethiopia: multicentre cross-sectional study. BMJ Open. 2021;11(12):e057708.

145. Chen CH, Yang PH, Kuo FL, Yeh IJ, Su CY. Experience of 2003 SARS has a negative psychological impact on healthcare workers in the COVID-19 pandemic: a cross-sectional study. Säo Paulo Med J [Internet]. 2021;139(1):65–71. Available from: http://www.scielo.br/scielo.php?script=sci_arttext&pid=S1516-31802021000100065

146. Ofei-Dodoo S, Loo-Gross C, Kellerman R. Burnout, Depression, Anxiety, and Stress Among Family Physicians in Kansas Responding to the COVID-19 Pandemic. J Am Board Fam Med. 2021;34(3):522–30.

147. Nguyen C, Young FG, McElroy D, Singh A. Personal protective equipment and adverse dermatological reactions among healthcare workers: Survey observations from the COVID-19 pandemic. Medicine (Baltimore) [Internet]. 2022;101(9):e29003. Available from: https://www.scopus.com/inward/record.uri?eid=2-s2.0-85125691189&doi=10.1097%2fMD.0000000000029003&partnerID=40&md5=cf568e115a2fd4f1d2d49cbd8cd3b3fe

148. Munawar K, Choudhry FR. Exploring stress coping strategies of frontline emergency health workers dealing Covid-19 in Pakistan: A qualitative inquiry. Am J Infect Control. 2021;49(3):286–92.

149. GebreEyesus FA, Tarekegn TT, Amlak BT, Shiferaw BZ, Emeria MS, Geleta OT, et al. Levels and predictors of anxiety, depression, and stress during COVID-19 pandemic among frontline healthcare providers in Gurage zonal public hospitals, Southwest Ethiopia, 2020: A multicenter cross-sectional study. PLoS One. 2021;16(11):e0259906.

150. Gómez-Salgado J, Ortega-Moreno M, Soriano G, Fagundo-Rivera J, Allande-Cussó R, Ruiz-Frutos C. History of contact with the SARS-COV-2 virus and the sense of coherence in the development of psychological distress in the occupational health professionals in Spain. Sci Prog. 2021;104(2):368504211026121.

151. Gong H, Zhang SX, Nawaser K, Afshar Jahanshahi A, Xu X, Li J, et al. The Mental Health of Healthcare Staff Working During the COVID-19 Crisis: Their Working Hours as a Boundary Condition. J Multidiscip Heal. 2021;14:1073–81.

152. Nie A, Su X, Zhang S, Guan W, Li J. Psychological impact of COVID-19 outbreak on frontline nurses: A cross-sectional survey study. J Clin Nurs. 2020;29(21):4217–26.

153. Azoulay E, Pochard F, Reignier J, Argaud L, Bruneel F, Courbon P, et al. Symptoms of Mental Health Disorders in Critical Care Physicians Facing the Second COVID-19 Wave: A Cross-Sectional Study. Chest. 2021;160(3):944–55.

154. Zhang X, Jiang Y, Yu H, Jiang Y, Guan Q, Zhao W, et al. Psychological and occupational impact on healthcare workers and its associated factors during the COVID-19 outbreak in China. Int Arch Occup Environ Health. 2021 Aug;94(6):1441–53.

155. Alrawashdeh HM, Al-Tammemi AB, Alzawahreh MK, Al-Tamimi A, Elkholy M, Al Sarireh F, et al. Occupational burnout and job satisfaction among physicians in times of COVID-19 crisis: a convergent parallel mixed-method study. BMC Public Health. 2021;21(1):811.

156. Teo I, Chay J, Cheung YB, Sung SC, Tewani KG, Yeo LF, et al. Healthcare worker stress, anxiety and burnout during the COVID-19 pandemic in Singapore: A 6-month multi-centre prospective study. PloS One. 2021;16(10):e0258866.

157. Lasater KB, Aiken LH, Sloane DM, French R, Martin B, Reneau K, et al. Chronic hospital nurse understaffing meets COVID-19: an observational study. BMJ Qual Saf. 2021;30(8):639–47.

158. Jawad MK, Al-Reda DAARA, Armeah WA, Abdulhussein AJ. Assessment of the burnout level in health care worker during covd 19. Indian J Forensic Med Toxicol [Internet]. 2021;15(3):1220–7. Available from: ["https://www.embase.com/search/results?subaction=viewrecord&id=L2007667577&from=export", "http://dx.doi.org/10.37506/ijfmt.v15i3.15478"]

159. Liu X, Chen J, Wang D, Li X, Wang E, Jin Y, et al. COVID-19 Outbreak Can Change the Job Burnout in Health Care Professionals. Front Psychiatry [Internet]. 2020;11. Available from: ["https://www.embase.com/search/results?subaction=viewrecord&id=L633726370&from=export", "http://dx.doi.org/10.3389/fpsyt.2020.563781"]

160. Alsulimani LK, Farhat AM, Borah RA, AlKhalifah JA, Alyaseen SM, Alghamdi SM, et al. Health care worker burnout during the COVID-19 pandemic: A cross-sectional survey study in Saudi Arabia. Saudi Med J. 2021;42(3):306–14.

161. Bruyneel A, Smith P, Tack J, Pirson M. Prevalence of burnout risk and factors associated with burnout risk among ICU nurses during the COVID-19 outbreak in French speaking Belgium. Intensive Crit Care Nurs. 2021;65:103059.

162. Butera S, Brasseur N, Filion N, Bruyneel A, Smith P. Prevalence and Associated Factors of Burnout Risk Among Intensive Care and Emergency Nurses Before and During the Coronavirus Disease 2019 Pandemic: A Cross-Sectional Study in Belgium. J Emerg Nurs. 2021;47(6):879–91.

163. Önen Sertöz Ö, Kuman Tunçel Ö, Sertöz N, Hepdurgun C, İşman Haznedaroğlu D, Bor C. Burnout in Healthcare Professionals During the Covid-19 Pandemic in a Tertiary Care University Hospital: Evaluation of the Need for Psychological Support. Turk Psikiyatri Derg. 2021;32(2):75–86.

164. Montoya V, Donnini K, Gauthier-Loiselle M, Sanon M, Cloutier M, Maitland J, et al. Mental Health and Health-Related Quality of Life Among Nephrology Nurses: A Survey-Based Cross-Sectional Study. Nephrol Nurs J. 2021;48(5):447–61.

165. Roberts R, Wong A, Jenkins S, Neher A, Sutton C, O’Meara P, et al. Mental health and well-being impacts of COVID-19 on rural paramedics, police, community nurses and child protection workers. Aust J Rural Health. 2021;29(5):753–67.

166. Ness MM, Saylor J, DiFusco LA, Evans K. Leadership, professional quality of life and moral distress during COVID-19: A mixed-methods approach. J Nurs Manag. 2021;29(8):2412–22.

167. Moreno-Mulet C, Sanso N, Carrero-Planells A, Lopez-Deflory C, Galiana L, Garcia-Pazo P, et al. The Impact of the COVID-19 Pandemic on ICU Healthcare Professionals: A Mixed Methods Study. Int J Environ Res Public Health. 2021 Sep;18(17).

168. Gemine R, Davies GR, Tarrant S, Davies RM, James M, Lewis K. Factors associated with work-related burnout in NHS staff during COVID-19: a cross-sectional mixed methods study. BMJ Open. 2021;11(1):e042591.

169. Hussain M, Amjad MB, Ahsan J, Minhas SO. Implementation Of National Institute Of Health Guidelines And Other Factors Contributing To Work-Related Burnout In Covid Isolation Ward And ICU Physicians. J Ayub Med Coll Abbottabad. 2021;33(2):283–8.

170. Prasad K, McLoughlin C, Stillman M, Poplau S, Goelz E, Taylor S, et al. Prevalence and correlates of stress and burnout among U.S. healthcare workers during the COVID-19 pandemic: A national cross-sectional survey study. EClinicalMedicine [Internet]. 2021;35. Available from: ["https://www.embase.com/search/results?subaction=viewrecord&id=L2012048610&from=export", "http://dx.doi.org/10.1016/j.eclinm.2021.100879"]

171. Khan N, Palepu A, Dodek P, Salmon A, Leitch H, Ruzycki S, et al. Cross-sectional survey on physician burnout during the COVID-19 pandemic in Vancouver, Canada: the role of gender, ethnicity and sexual orientation. BMJ Open. 2021;11(5):e050380.

172. Cahill AG, Olshavsky ME, Newport DJ, Benzer J, Chambers KM, Custer J, et al. Occupational Risk Factors and Mental Health Among Frontline Health Care Workers in a Large US Metropolitan Area During the COVID-19 Pandemic. Prim Care Companion CNS Disord [Internet]. 2022;24(2). Available from: https://www.scopus.com/inward/record.uri?eid=2-s2.0-85126389443&doi=10.4088%2fPCC.21m03166&partnerID=40&md5=d33edf4596c709e85a01695c4ab7095c

173. Di Giuseppe M, Nepa G, Prout TA, Albertini F, Marcelli S, Orrù G, et al. Stress, Burnout, and Resilience among Healthcare Workers during the COVID-19 Emergency: The Role of Defense Mechanisms. Int J Env Res Public Health. 2021;18(10).

174. Duarte I, Teixeira A, Castro L, Marina S, Ribeiro C, Jácome C, et al. Burnout among Portuguese healthcare workers during the COVID-19 pandemic. BMC Public Health. 2020;20(1):1885.

175. Nishimura Y, Miyoshi T, Hagiya H, Kosaki Y, Otsuka F. Burnout of Healthcare Workers amid the COVID-19 Pandemic: A Japanese Cross-Sectional Survey. Int J Env Res Public Health. 2021;18(5).

176. Kim JS, Choi JS. Factors Influencing Emergency Nurses’ Burnout During an Outbreak of Middle East Respiratory Syndrome Coronavirus in Korea. Asian Nurs Res. 2016 Dec;10(4):295–9.

177. Kim SC, Rankin L, Ferguson J. Nurses’ mental health from early COVID-19 pandemic to vaccination. J Nurs Sch. 2021;

178. Sung CW, Chen CH, Fan CY, Chang JH, Hung CC, Fu CM, et al. Mental health crisis in healthcare providers in the COVID-19 pandemic: a cross-sectional facility-based survey. BMJ Open. 2021;11(7):e052184.

179. Tarhan Ş, Şimşek GÖ, Tecirli ND, Uçan ES, Atik M, İtil BO, et al. Facing the pandemic: Burnout in physicians in turkey. Turk Thorac J [Internet]. 2021;22(6):439–45. Available from: ["https://www.embase.com/search/results?subaction=viewrecord&id=L2014311575&from=export", "http://dx.doi.org/10.5152/TurkThoracJ.2021.20240"]

180. Ali SK, Shah J, Talib Z. COVID-19 and mental well-being of nurses in a tertiary facility in Kenya. PLoS One. 2021;16(7):e0254074.

181. Lee HA, Ahn MH, Byun S, Lee HK, Kweon YS, Chung S, et al. How COVID-19 Affected Healthcare Workers in the Hospital Locked Down due to Early COVID-19 Cases in Korea. J Korean Med Sci. 2021;36(47):e325.

182. Yılmaz Y, Erdoğan A, Bahadır E. Fear, Anxiety, Burnout, and Insomnia Levels of Healthcare Workers during COVID-19 Pandemic in Turkey. Psychiatr Danub. 2021;33:350–6.

183. Chen R, Sun C, Chen JJ, Jen HJ, Kang XL, Kao CC, et al. A Large-Scale Survey on Trauma, Burnout, and Posttraumatic Growth among Nurses during the COVID-19 Pandemic. Int J Ment Health Nurs. 2021;30(1):102–16.

184. Lin YY, Pan YA, Hsieh YL, Hsieh MH, Chuang YS, Hsu HY, et al. COVID-19 Pandemic Is Associated with an Adverse Impact on Burnout and Mood Disorder in Healthcare Professionals. Int J Env Res Public Health. 2021;18(7).

185. Gonçalves JV, Castro L, Rêgo G, Nunes R. Burnout Determinants among Nurses Working in Palliative Care during the Coronavirus Disease 2019 Pandemic. Int J Env Res Public Health. 2021;18(7).

186. Ibrahim F, Samsudin EZ, Chen XW, Toha HR. The Prevalence and Work-Related Factors of Burnout Among Public Health Workforce During the COVID-19 Pandemic. J Occup Env Med. 2022;64(1):e20–7.

187. Sengul H, Bulut A, Kahraman B. The impact of changing processes in the COVID-19 pandemic on health care workers’ burnout syndrome: Web-based questionnaire study. Haseki Tip Bul [Internet]. 2021;59:36–44. Available from: ["https://www.embase.com/search/results?subaction=viewrecord&id=L2013008206&from=export", "http://dx.doi.org/10.4274/haseki.galenos.2021.7130"]

188. Vitale E, Lupo R, Calabrò A, Cornacchia M, Conte L, Marchisio D, et al. Mapping potential risk factors in developing burnout syndrome between physicians and registered nurses suffering from an aggression in Italian emergency departments. J Psychopathol [Internet]. 2021;27(3):148–55. Available from: https://www.scopus.com/inward/record.uri?eid=2-s2.0-85117367543&doi=10.36148%2f2284-0249-425&partnerID=40&md5=3dbddd457dc3ad6e20ae1af8a1f49bec

189. Nguyen J, Liu A, McKenney M, Liu H, Ang D, Elkbuli A. Impacts and challenges of the COVID-19 pandemic on emergency medicine physicians in the United States. Am J Emerg Med. 2021;48:38–47.

190. Vitale E, Casolaro S. Anxiety, burnout and depression levels according to sex and years of work experience in italian nurses engaged in the care of covid-19 patients. J Evid-Based Psychother [Internet]. 2021;21(1):83–96. Available from: https://www.scopus.com/inward/record.uri?eid=2-s2.0-85108065262&doi=10.24193%2fjebp.2021.1.6&partnerID=40&md5=1056952e50ddc2dcda2e21b6c0b426d9

191. Khasne RW, Dhakulkar BS, Mahajan HC, Kulkarni AP. Burnout among healthcare workers during COVID-19 pandemic in india: Results of a questionnaire-based survey. Indian J Crit Care Med [Internet]. 2020;24(8):664–71. Available from: ["https://www.embase.com/search/results?subaction=viewrecord&id=L632900300&from=export", "http://dx.doi.org/10.5005/jp-journals-10071-23518"]

192. Banerjee S, Lim KHJ, Murali K, Kamposioras K, Punie K, Oing C, et al. The impact of COVID-19 on oncology professionals: results of the ESMO Resilience Task Force survey collaboration. ESMO Open. 2021;6(2):100058.

193. Brera AS, Arrigoni C, Dellafiore F, Odone A, Magon A, Nania T, et al. Burnout syndrome and its determinants among healthcare workers during the first wave of the Covid-19 outbreak in Italy: a cross-sectional study to identify sex-related differences. Med Lav. 2021;112(4):306–19.

194. Lange M, Joo S, Couette PA, Le Bas F, Humbert X. Impact on mental health of the COVID-19 outbreak among general practitioners during the sanitary lockdown period. Ir J Med Sci. 2022;191(1):93–6.

195. Stone KW, Kintziger KW, Jagger MA, Horney JA. Public Health Workforce Burnout in the COVID-19 Response in the U.S. Int J Environ Res Public Health [Internet]. 2021 Jan [cited 2021 Oct 18];18(8):4369. Available from: https://www.mdpi.com/1660-4601/18/8/4369

196. Zhou LL, Zhang SE, Liu J, Wang HN, Liu L, Zhou JJ, et al. Demographic Factors and Job Characteristics Associated With Burnout in Chinese Female Nurses During Controlled COVID-19 Period: A Cross-Sectional Study. Front Public Health. 2021;9:757113.

197. Dillon EC, Stults CD, Deng S, Martinez M, Szwerinski N, Koenig PT, et al. Women, Younger Clinicians’, and Caregivers’ Experiences of Burnout and Well-being During COVID-19 in a US Healthcare System. J Gen Intern Med. 2022;37(1):145–53.

198. Li X, Jiang T, Sun J, Shi L, Liu J. The relationship between occupational stress, job burnout and quality of life among surgical nurses in Xinjiang, China. BMC Nurs [Internet]. 2021;20(1). Available from: https://www.scopus.com/inward/record.uri?eid=2-s2.0-85115841287&doi=10.1186%2fs12912-021-00703-2&partnerID=40&md5=02e811e43eaded9dfc56c2a8d0610694

199. Lee JY, Kim M, Jhon M, Kim H, Kang HJ, Ryu S, et al. The association of gratitude with perceived stress among nurses in Korea during COVID-19 outbreak. Arch Psychiatr Nurs. 2021;35(6):647–52.

200. Sarboozi Hoseinabadi T, Kakhki S, Teimori G, Nayyeri S. Burnout and its influencing factors between frontline nurses and nurses from other wards during the outbreak of Coronavirus Disease -COVID-19- in Iran. Invest Educ Enferm. 2020;38(2).

201. Wang B, Lu Q, Sun F, Zhang R. The relationship between sleep quality and psychological distress and job burnout among chinese psychiatric nurses. Ind Health [Internet]. 2021;59(6):427–35. Available from: https://www.scopus.com/inward/record.uri?eid=2-s2.0-85120380478&doi=10.2486%2findhealth.2020-0249&partnerID=40&md5=85c09554ef564dd6b6c61823c2691213

202. Alkhamees AA, Assiri H, Alharbi HY, Nasser A, Alkhamees MA. Burnout and depression among psychiatry residents during COVID-19 pandemic. Hum Resour Health. 2021;19(1):46.

203. Yetneberk T, Firde M, Eshetie D, Tiruneh A, Moore J. The prevalence of burnout syndrome and its association with adherence to safety and practice standards among anesthetists working in Ethiopia. Ann Med Surg [Internet]. 2021;69. Available from: ["https://www.embase.com/search/results?subaction=viewrecord&id=L2014401753&from=export", "http://dx.doi.org/10.1016/j.amsu.2021.102777"]

204. Işik M, Kirli U, Özdemir PG. The Mental Health of Healthcare Professionals During the COVID-19 Pandemic. Turk Psikiyatri Derg. 2021;32(4):225–34.

205. Yang X, Chen D, Chen Y, Wang N, Lyv C, Li Y, et al. Geographical distribution and prevalence of mental disorders among healthcare workers in China: A cross-sectional country-wide survey: A cross-sectional study to assess mental disorders of healthcare workers in China. Int J Health Plann Manage. 2021;36(5):1561–74.

206. Alonso J, Vilagut G, Mortier P, Ferrer M, Alayo I, Aragón-Peña A, et al. Mental health impact of the first wave of COVID-19 pandemic on Spanish healthcare workers: A large cross-sectional survey. Rev Psiquiatr Salud Ment Engl Ed. 2021;14(2):90–105.

207. Bassi M, Negri L, Delle Fave A, Accardi R. The relationship between post-traumatic stress and positive mental health symptoms among health workers during COVID-19 pandemic in Lombardy, Italy. J Affect Disord. 2021;280:1–6.

208. Apple SJ, Wilson J, Gao H, Ma X, Saad A, Huang A, et al. 38 Posttraumatic Stress in Emergency Department Health Care Workers During the COVID-19 Outbreak in Brooklyn, New York. Ann Emerg Med [Internet]. 2021;78(2):S20. Available from: ["https://www.embase.com/search/results?subaction=viewrecord&id=L2013858371&from=export", "http://dx.doi.org/10.1016/j.annemergmed.2021.07.039"]

209. Asnakew S, Legas G, Muche Liyeh T, Belete A, Haile K, Yitbarek GY, et al. Prevalence of post-traumatic stress disorder on health professionals in the era of COVID-19 pandemic, Northwest Ethiopia, 2020: A multi-centered cross-sectional study. PLoS One. 2021;16(9):e0255340.

210. Ayotte BJ, Schierberl Scherr AE, Kellogg MB. PTSD Symptoms and Functional Impairment among Nurses Treating COVID-19 Patients. SAGE Open Nurs. 2022;8:23779608221074652.

211. Laurent A, Fournier A, Lheureux F, Poujol AL, Deltour V, Ecarnot F, et al. Risk and protective factors for the possible development of post-traumatic stress disorder among intensive care professionals in France during the first peak of the COVID-19 epidemic. Eur J Psychotraumatol. 2022;13(1):2011603.

212. Sarapultseva M, Zolotareva A, Kritsky I, Nasretdinova N, Sarapultsev A. Psychological Distress and Post-Traumatic Symptomatology among Dental Healthcare Workers in Russia: Results of a Pilot Study. Int J Environ Res Public Health. 2021 Jan;18(2).

213. Chatzittofis A, Constantinidou A, Artemiadis A, Michailidou K, Karanikola MNK. The Role of Perceived Organizational Support in Mental Health of Healthcare Workers During the COVID-19 Pandemic: A Cross-Sectional Study. Front Psychiatry. 2021;12:707293.

214. Cockerham M, Beier ME, Branson S, Boss L. Nurse Adaptability and Post-traumatic Stress Disorder Symptoms During the COVID-19 Pandemic: The Effects of Family and Perceived Organizational Support. Front Psychol. 2021;12:749763.

215. Bizri M, Kassir G, Tamim H, Kobeissy F, Hayek SE. Psychological distress experienced by physicians and nurses at a tertiary care center in Lebanon during the COVID-19 outbreak. J Health Psychol. 2021;1359105321991630.

216. Blekas A, Voitsidis P, Athanasiadou M, Parlapani E, Chatzigeorgiou AF, Skoupra M, et al. COVID-19: PTSD symptoms in Greek health care professionals. Psychol Trauma. 2020;12(7):812–9.

217. Miguel-Puga JA, Cooper-Bribiesca D, Avelar-Garnica FJ, Sanchez-Hurtado LA, Colin-Martínez T, Espinosa-Poblano E, et al. Burnout, depersonalization, and anxiety contribute to post-traumatic stress in frontline health workers at COVID-19 patient care, a follow-up study. Brain Behav. 2021;11(3):e02007.

218. Hines SE, Chin KH, Glick DR, Wickwire EM. Trends in Moral Injury, Distress, and Resilience Factors among Healthcare Workers at the Beginning of the COVID-19 Pandemic. Int J Env Res Public Health. 2021;18(2).

219. Leng M, Wei L, Shi X, Cao G, Wei Y, Xu H, et al. Mental distress and influencing factors in nurses caring for patients with COVID-19. Nurs Crit Care. 2021;26(2):94–101.

220. Bismark M, Scurrah K, Pascoe A, Willis K, Jain R, Smallwood N. Thoughts of suicide or self-harm among Australian healthcare workers during the COVID-19 pandemic. Aust N Z J Psychiatry. 2022;48674221075540.

221. Bruffaerts R, Voorspoels W, Jansen L, Kessler RC, Mortier P, Vilagut G, et al. Suicidality among healthcare professionals during the first COVID19 wave. J Affect Disord. 2021;283:66–70.

222. Hong S, Ai M, Xu X, Wang W, Chen J, Zhang Q, et al. Immediate psychological impact on nurses working at 42 government-designated hospitals during COVID-19 outbreak in China: A cross-sectional study. Nurs Outlook. 2021 Feb;69(1):6–12.

223. Bryant-Genevier J, Rao CY, Lopes-Cardozo B, Kone A, Rose C, Thomas I, et al. Symptoms of Depression, Anxiety, Post-Traumatic Stress Disorder, and Suicidal Ideation Among State, Tribal, Local, and Territorial Public Health Workers During the COVID-19 Pandemic - United States, March-April 2021. MMWR Morb Mortal Wkly Rep. 2021;70(26):947–52.

224. Mortier P, Vilagut G, Ferrer M, Serra C, Molina JD, López-Fresneña N, et al. Thirty-day suicidal thoughts and behaviors among hospital workers during the first wave of the Spain COVID-19 outbreak. Depress Anxiety. 2021;38(5):528–44.

225. Ortiz-Calvo E, Martínez-Alés G, Mediavilla R, González-Gómez E, Fernández-Jiménez E, Bravo-Ortiz MF, et al. The role of social support and resilience in the mental health impact of the COVID-19 pandemic among healthcare workers in Spain. J Psychiatr Res. 2022;148:181–7.

226. Lee SM, Kang WS, Cho AR, Kim T, Park JK. Psychological impact of the 2015 MERS outbreak on hospital workers and quarantined hemodialysis patients. Compr Psychiatry. 2018;87:123–7.

227. Cabello M, Izquierdo A, Leal I. Loneliness and not living alone is what impacted on the healthcare professional’s mental health during the COVID-19 outbreak in Spain. Health Soc Care Community. 2021;

228. Cardoso MFPT, Martins MMFP da S, Trindade L de L, Ribeiro OMPL, Fonseca EF. The COVID-19 pandemic and nurses’ attitudes toward death. Rev Latinoam Enferm Online [Internet]. 2021;29:e3448–e3448. Available from: http://www.scielo.br/scielo.php?script=sci_arttext&pid=S0104-11692021000100361

229. Bredicean C, Tamasan SC, Lungeanu D, Giurgi-Oncu C, Stoica IP, Panfil AL, et al. Burnout toll on empathy would mediate the missing professional support in the COVID-19 outbreak. Risk Manag Healthc Policy [Internet]. 2021;14:2231–44. Available from: ["https://www.embase.com/search/results?subaction=viewrecord&id=L2007497019&from=export", "http://dx.doi.org/10.2147/RMHP.S300578"]

230. Zhan YX, Zhao SY, Yuan J, Liu H, Liu YF, Gui LL, et al. Prevalence and Influencing Factors on Fatigue of First-line Nurses Combating with COVID-19 in China: A Descriptive Cross-Sectional Study. Curr Med Sci. 2020;40(4):625–35.

231. Llorente-Alonso M, García-Ael C, Topa G, Sanz-Muñoz ML, Muñoz-Alcalde I, Cortés-Abejer B. Can psychological empowerment prevent emotional disorders in presence of fear of COVID-19 in health workers? A cross-sectional validation study. J Clin Med [Internet]. 2021;10(8). Available from: ["https://www.embase.com/search/results?subaction=viewrecord&id=L2006922887&from=export", "http://dx.doi.org/10.3390/jcm10081614"]

232. Labrague LJ. Pandemic fatigue and clinical nurses’ mental health, sleep quality and job contentment during the covid-19 pandemic: The mediating role of resilience. J Nurs Manag. 2021;29(7):1992–2001.

233. Zhou Y, Ding H, Zhang Y, Zhang B, Guo Y, Cheung T, et al. Prevalence of poor psychiatric status and sleep quality among frontline healthcare workers during and after the COVID-19 outbreak: a longitudinal study. Transl Psychiatry. 2021;11(1):223.

234. Nikeghbal K, Kouhnavard B, Shabani A, Zamanian Z. Covid-19 Effects on the Mental Workload and Quality of Work Life in Iranian Nurses. Ann Glob Health. 2021;87(1):79.

235. Beiter KJ, Wiedemann RP, Thomas CL, Conrad EJ. Alcohol Consumption and COVID-19-Related Stress Among Health Care Workers: The Need for Continued Stress-Management Interventions. Public Health Rep. 2022;137(2):326–35.

236. Diaz F, Cornelius T, Bramley S, Venner H, Shaw K, Dong M, et al. The association between sleep and psychological distress among New York City healthcare workers during the COVID-19 pandemic. J Affect Disord. 2022;298:618–24.

237. Alshekaili M, Hassan W, Al Said N, Al Sulaimani F, Jayapal SK, Al-Mawali A, et al. Factors associated with mental health outcomes across healthcare settings in Oman during COVID-19: frontline versus non-frontline healthcare workers. BMJ Open. 2020 Oct 10;10(10):e042030.

238. Qi J, Xu J, Li BZ, Huang JS, Yang Y, Zhang ZT, et al. The evaluation of sleep disturbances for Chinese frontline medical workers under the outbreak of COVID-19. Sleep Med. 2020;72:1–4.

239. Ali M, Uddin Z, Ahsan NF, Haque MZ, Bairagee M, Khan SA, et al. Prevalence of mental health symptoms and its effect on insomnia among healthcare workers who attended hospitals during COVID-19 pandemic: A survey in Dhaka city. Heliyon. 2021;7(5):e06985.

240. Ong JJY, Bharatendu C, Goh Y, Tang JZY, Sooi KWX, Tan YL, et al. Headaches Associated With Personal Protective Equipment - A Cross-Sectional Study Among Frontline Healthcare Workers During COVID-19. Headache. 2020;60(5):864–77.

241. Jose MRM, David C, Alberto G, Belen R, Pablo M, Roshan H, et al. Mask-associated de novo headache in healthcare workers during the Covid-19 pandemic. 2020; Available from: https://medrxiv.org/cgi/content/short/2020.08.07.20167957

242. Joy KMN, Mahmud R, Rabbani G, Islam MK, Khalil MI, Kundu NC. Mask and Personal Protective Equipment (PPE) Associated Headache among the COVID-Time Physicians of Bangladesh. J Headache Pain [Internet]. 2021;22. Available from: ["https://www.embase.com/search/results?subaction=viewrecord&id=L636670146&from=export", "http://dx.doi.org/10.1186/s10194-021-01319-2"]

243. Marfil-Rivera A, Marfil-Garza BA, Fernández-Garza LE. [Headache associated with the use of personal protective equipment during the COVID-19 pandemic: an international survey]. Rev Neurol. 2021;73(5):151–64.

244. Zaheer R, Khan M, Tanveer A, Farooq A, Khurshid Z. Association of Personal Protective Equipment with De Novo Headaches in Frontline Healthcare Workers during COVID-19 Pandemic: A Cross-Sectional Study. Eur J Dent. 2020;14:S79-s85.

245. Rapisarda L, Trimboli M, Fortunato F, De Martino A, Marsico O, Demonte G, et al. Facemask headache: a new nosographic entity among healthcare providers in COVID-19 era. Neurol Sci. 2021;42(4):1267–76.

246. Christopher PM, Roren RS, Tania C, Jayadi NN, Cucunawangsih C. Adverse Skin Reactions to Personal Protective Equipment among Health-Care Workers during COVID-19 Pandemic: A Multicenter Cross-sectional Study in Indonesia. Int J Dermatol Venereol [Internet]. 2020;3(4):211–8. Available from: https://www.scopus.com/inward/record.uri?eid=2-s2.0-85108505360&doi=10.1097%2fJD9.0000000000000132&partnerID=40&md5=8a4df988203816290fa5a04c79bb3dfe

247. Marraha F, Al Faker I, Charif F, Chahoub H, Benyamna Y, Rahmani N, et al. Skin Reactions to Personal Protective Equipment among First-Line COVID-19 Healthcare Workers: A Survey in Northern Morocco. Ann Work Expo Health. 2021;65(8):998–1003.

248. Coelho M de MF, Cavalcante VMV, Moraes JT, Menezes LCG de, Figueirêdo SV, Branco MFCC, et al. Pressure injury related to the use of personal protective equipment in COVID-19 pandemic. Rev Bras Enferm [Internet]. 2020;73:e20200670–e20200670. Available from: http://www.revenf.bvs.br/scielo.php?script=sci_arttext&pid=S0034-71672020000900159

249. Gürlek Kısacık Ö, Özyürek P. Skin-related problems associated with the use of personal protective equipment among health care workers during the COVID-19 pandemic: A online survey study. J Tissue Viability. 2022;31(1):112–8.

250. Çağlar A, Kaçer İ, Hacımustafaoğlu M, Öztürk B, Öztürk K. Symptoms Associated With Personal Protective Equipment Among Frontline Health Care Professionals During the COVID-19 Pandemic. Disaster Med Public Health Prep. 2020;1–4.

251. Daye M, Cihan FG, Durduran Y. Evaluation of skin problems and dermatology life quality index in health care workers who use personal protection measures during COVID-19 pandemic. Dermatol Ther. 2020;33(6):e14346.

252. Aborisade RA, Gbahabo DD. Policing the lockdown: accounts of police officers’ aggression and extortion of frontline health workers in Nigeria. Polic Soc [Internet]. 2021;31(5):565–82. Available from: https://www.scopus.com/inward/record.uri?eid=2-s2.0-85103174055&doi=10.1080%2f10439463.2021.1903461&partnerID=40&md5=2ad6e186bc182ff91e79db804aea805f

253. Gee S, Skovdal M. Public Discourses of Ebola Contagion and Courtesy Stigma: The Real Risk to International Health Care Workers Returning Home From the West Africa Ebola Outbreak? Qual Health Res. 2018;28(9):1499–508.

254. Shang Z, Kim JY, Cheng SO. Discrimination experienced by Asian Canadian and Asian American health care workers during the COVID-19 pandemic: a qualitative study. CMAJ Open. 2021;9(4):E998-e1004.

255. Muñoz Del Carpio-Toia A, Begazo Muñoz Del Carpio L, Mayta-Tristan P, Alarcón-Yaquetto DE, Málaga G. Workplace Violence Against Physicians Treating COVID-19 Patients in Peru: A Cross-Sectional Study. Jt Comm J Qual Patient Saf. 2021;47(10):637–45.

256. Bitencourt MR, Alarcão ACJ, Silva LL, Dutra AC, Caruzzo NM, Roszkowski I, et al. Predictors of violence against health professionals during the COVID-19 pandemic in Brazil: A cross-sectional study. PLoS One. 2021;16(6):e0253398.

257. Xie XM, Zhao YJ, An FR, Zhang QE, Yu HY, Yuan Z, et al. Workplace violence and its association with quality of life among mental health professionals in China during the COVID-19 pandemic. J Psychiatr Res. 2021 Mar;135:289–93.

258. Mostafa A, Sabry W, Mostafa NS. COVID-19-related stigmatization among a sample of Egyptian healthcare workers. PLoS One. 2020;15(12):e0244172.

259. Alsaqri S, Pangket P, Alkuwaisi M, Llego J, Alshammari MS. COVID-19 associated social stigma as experienced by frontline nurses of Hail: A qualitative study. Int J Adv Appl Sci [Internet]. 2021;8(8):52–7. Available from: https://www.scopus.com/inward/record.uri?eid=2-s2.0-85108613339&doi=10.21833%2fIJAAS.2021.08.007&partnerID=40&md5=eb259ed1208453afcc90b0af8f27d6a5

260. Gunawan J, Juthamanee S, Aungsuroch Y. Current Mental Health Issues in the Era of Covid-19. ASIAN J PSYCHIATRY. 2020 Jun;51.

261. Sachdeva A, Nandini H, Kumar V, Chawla RK, Chopra K. From stress to stigma – Mental health considerations of health care workers involved in COVID19 management. Indian J Tuberc [Internet]. 2021; Available from: ["https://www.embase.com/search/results?subaction=viewrecord&id=L2014888570&from=export", "http://dx.doi.org/10.1016/j.ijtb.2021.09.007"]

262. Ghareeb NS, El-Shafei DA, Eladl AM. Workplace violence among healthcare workers during COVID-19 pandemic in a Jordanian governmental hospital: the tip of the iceberg. Environ Sci Pollut Res. 2021 Nov;28(43):61441–9.

263. Ceri V, Cicek I. Psychological Well-Being, Depression and Stress During COVID-19 Pandemic in Turkey: A Comparative Study of Healthcare Professionals and Non-Healthcare Professionals. Psychol Health Med. 2021 Jan;26(1):85–97.

264. Wozniak H, Benzakour L, Moullec G, Buetti N, Nguyen A, Corbaz S, et al. Mental health outcomes of ICU and non-ICU healthcare workers during the COVID-19 outbreak: a cross-sectional study. Ann Intensive Care. 2021;11(1):106.

265. Safiye T, Vukcevic B, Cabarkapa M. Resilience as a moderator in the relationship between burnout and subjective well-being among medical workers in Serbia during the COVID-19 pandemic. Vojnosanit Pregl [Internet]. 2021;78(11):1207–13. Available from: ["https://www.embase.com/search/results?subaction=viewrecord&id=L2016063948&from=export", "http://dx.doi.org/10.2298/VSP210517070S"]

266. Abdelghani M, Mahdy RS, El-Gohari HM. Health anxiety to COVID-19 virus infection and its relationship to quality of life in a sample of health care workers in Egypt: A cross-sectional study. Arch Psychiatry Psychother [Internet]. 2021;23(1):19–28. Available from: ["https://www.embase.com/search/results?subaction=viewrecord&id=L2011720988&from=export", "http://dx.doi.org/10.12740/APP/130304"]

267. Mohamadzadeh Tabrizi Z, Mohammadzadeh F, Davarinia Motlagh Quchan A, Bahri N. COVID-19 anxiety and quality of life among Iranian nurses. BMC Nurs. 2022;21(1):27.

268. McFadden P, Ross J, Moriarty J, Mallett J, Schroder H, Ravalier J, et al. The Role of Coping in the Wellbeing and Work-Related Quality of Life of UK Health and Social Care Workers during COVID-19. Int J Env Res Public Health. 2021;18(2).

269. Ebrahimi H, Jafarjalal E, Lotfolahzadeh A, Kharghani Moghadam SM. The effect of workload on nurses’ quality of life with moderating perceived social support during the COVID-19 pandemic. Work. 2021;70(2):347–54.

270. Caliari JS, Santos MAD, Andrechuk CRS, Campos KRC, Ceolim MF, Pereira FH. Quality of life of nurse practitioners during the COVID-19 pandemic. Rev Bras Enferm. 2021;75:e20201382.

271. Baysal E, Selçuk AK, Aktan GG, Andrade EF, Notarnicola I, Stievano A, et al. An examination of the fear of COVID-19 and professional quality of life among nurses: A multicultural study. J Nurs Manag. 2022;

272. Lehmann M, Bruenahl CA, Addo MM, Becker S, Schmiedel S, Lohse AW, et al. Acute Ebola virus disease patient treatment and health-related quality of life in health care professionals: A controlled study. J Psychosom Res. 2016;83:69–74.

273. Chalhub R, Menezes MS, Aguiar CVN, Santos-Lins LS, Netto EM, Brites C, et al. Anxiety, health-related quality of life, and symptoms of burnout in frontline physicians during the COVID-19 pandemic. Braz J Infect Dis. 2021;25(5):101618.

274. Choi HJ, Yang CM, Lee SY, Lee HJ, Jang SH. Mental Health and Quality of Life for Healthcare Workers in a University Hospital Under COVID-19. Psychiatry Investig. 2022;19(2):85–91.

275. Alhawatmeh H, Alsholol R, Aldelky H, Al-Ali N, Albataineh R. Mediating role of resilience on the relationship between stress and quality of life among Jordanian registered nurses during COVID-19 pandemic. Heliyon. 2021;7(11):e08378.

276. Almhdawi KA, Alrabbaie H, Arabiat A, Alhammouri AT, Hamadneh M, Obeidat D, et al. Physicians’ Health-Related Quality of Life and Its Associated Factors During COVID-19 Pandemic in Jordan: A Cross-Sectional Study. Eval Health Prof. 2022;1632787211068899.

277. Kantorski LP, de Oliveira MM, Treichel CAS, Alves PF, Lemos DSC, Ramos CI. Suspected infection, absenteeism at work and testing for covid-19 among nursing professionals. Texto E Contexto Enferm [Internet]. 2021;30. Available from: https://www.scopus.com/inward/record.uri?eid=2-s2.0-85121446330&doi=10.1590%2f1980-265X-TCE-2021-0135&partnerID=40&md5=e376be55ea04252bbcb998d43559789c

278. Maltezou HC, Panagopoulos P, Sourri F, Giannouchos T V, Raftopoulos V, Gamaletsou MN, et al. COVID-19 vaccination significantly reduces morbidity and absenteeism among healthcare personnel: A prospective multicenter study. VACCINE. 2021 Nov 26;39(48):7021–7.

279. Dolić M, Antičević V, Dolić K, Pogorelić Z. Difference in Pandemic-Related Experiences and Factors Associated with Sickness Absence among Nurses Working in COVID-19 and Non-COVID-19 Departments. Int J Env Res Public Health. 2022;19(3).

280. Khorasanee R, Grundy T, Isted A, Breeze R. The effects of COVID-19 on sickness of medical staff across departments: A single centre experience. Clin Med Lond. 2021;21(2):e150–4.

281. Gindler J, Grohskopf LA, Biggerstaff M, Finelli L. A model survey for assessing 2009 pandemic influenza A (H1N1) virus disease burden in the workplace. Clin Infect Dis. 2011;52:S173-6.

282. Li TM, Pien LC, Kao CC, Kubo T, Cheng WJ. Effects of work conditions and organisational strategies on nurses’ mental health during the COVID-19 pandemic. J Nurs Manag. 2022;30(1):71–8.

283. Khattak SR, Saeed I, Rehman SU, Fayaz M. Impact of Fear of COVID-19 Pandemic on the Mental Health of Nurses in Pakistan. J Loss Trauma [Internet]. 2021;26(5):421–35. Available from: https://www.scopus.com/inward/record.uri?eid=2-s2.0-85091134759&doi=10.1080%2f15325024.2020.1814580&partnerID=40&md5=f1dd1a295088ef1f22788648ded65297

284. Ohue T, Togo E, Ohue Y, Mitoku K. Mental health of nurses involved with COVID-19 patients in Japan, intention to resign, and influencing factors. Med Baltim. 2021;100(31):e26828.

285. Yáñez JA, Afshar Jahanshahi A, Alvarez-Risco A, Li J, Zhang SX. Anxiety, Distress, and Turnover Intention of Healthcare Workers in Peru by Their Distance to the Epicenter during the COVID-19 Crisis. Am J Trop Med Hyg. 2020;103(4):1614–20.

286. Alameddine M, Clinton M, Bou-Karroum K, Richa N, Doumit MAA. Factors Associated With the Resilience of Nurses During the COVID-19 Pandemic. Worldviews Evid Based Nurs. 2021;18(6):320–31.

1. Most relevant, not exhaustive [↑](#footnote-ref-1)
