# Supplementary material for: Impacts for health and care workers of Covid-19 and other public health emergencies of international concern: living systematic review, meta-analysis and policy recommendations
Source: Hum Resour Health. 2024 Jan 25;22:10. doi: 10.1186/s12960-024-00892-2 (PMC10809470; doi:10.1186/s12960-024-00892-2)
Supplement: Supplementary file 10 — Additional file 10. Cost-effective and culturally relevant interventions to address short- and long-term impact of COVID-19 pandemic and other PHEICs on HCW. [file 12960_2024_892_MOESM10_ESM.docx]

[Items in the CAT checklists 1](#_Toc135240343)

[COMPUTATION OF SCORES AND QUALITY CATEGORIES FOR THE JBI CRITICAL APPRAISAL TOOLS (CAT) 4](#_Toc135240344)

[Quality of evidence assessment criteria 5](#_Toc135240345)

# Items in the CAT checklists

| **CHECKLIST FOR ANALYTICAL CROSS-SECTIONAL STUDIES**  1. Were the criteria for inclusion in the sample clearly defined?  2. Were the study subjects and the setting described in detail?  3. Was the exposure measured in a valid and reliable way?  4. Were objective, standard criteria used for measurement of the condition?  5. Were confounding factors identified?  6. Were strategies to deal with confounding factors stated?  7. Were the outcomes measured in a valid and reliable way?  8. Was appropriate statistical analysis used? |
| --- |
| **CHECKLIST FOR CASE CONTROL STUDIES**  1. Were the groups comparable other than the presence of disease in cases or the absence of disease in controls?  2. Were cases and controls matched appropriately?  3. Were the same criteria used for identification of cases and controls?  4. Was exposure measured in a standard, valid and reliable way?  5. Was exposure measured in the same way for cases and controls?  6. Were confounding factors identified?  7. Were strategies to deal with confounding factors stated?  8. Were outcomes assessed in a standard, valid and reliable way for cases and controls?  9. Was the exposure period of interest long enough to be meaningful?  10. Was appropriate statistical analysis used? |
| **CHECKLIST FOR COHORT STUDIES**  1. Were the two groups similar and recruited from the same population?  2. Were the exposures measured similarly to assign people to both exposed and unexposed groups?  3. Was the exposure measured in a valid and reliable way?  4. Were confounding factors identified?  5. Were strategies to deal with confounding factors stated?  6. Were the groups/participants free of the outcome at the start of the study (or at the moment of exposure)?  7. Were the outcomes measured in a valid and reliable way?  8. Was the follow up time reported and sufficient to be long enough for outcomes to occur?  9. Was follow up complete, and if not, were the reasons to loss to follow up described and explored?  10. Were strategies to address incomplete follow up utilized?  11. Was appropriate statistical analysis used? |
| **CHECKLIST FOR STUDIES REPORTING PREVALENCE DATA**  1. Was the sample frame appropriate to address the target population?  2. Were study participants sampled in an appropriate way?  3. Was the sample size adequate?  4. Were the study subjects and the setting described in detail?  5. Was the data analysis conducted with sufficient coverage of the identified sample?  6. Were valid methods used for the identification of the condition?  7. Was the condition measured in a standard, reliable way for all participants?  8. Was there appropriate statistical analysis?  9. Was the response rate adequate, and if not, was the low response rate managed appropriately? |
| **CHECKLIST FOR QUALITATIVE RESEARCH**  1. Is there congruity between the stated philosophical perspective and the research methodology?  2. Is there congruity between the research methodology and the research question or objectives?  3. Is there congruity between the research methodology and the methods used to collect data?  4. Is there congruity between the research methodology and the representation and analysis of data?  5. Is there congruity between the research methodology and the interpretation of results?  6. Is there a statement locating the researcher culturally or theoretically?  7. Is the influence of the researcher on the research, and vice- versa, addressed?  8. Are participants, and their voices, adequately represented?  9. Is the research ethical according to current criteria or, for recent studies, and is there evidence of ethical approval by an appropriate body?  10. Do the conclusions drawn in the research report flow from the analysis, or interpretation, of the data? |
| **CHECKLIST FOR QUASI-EXPERIMENTAL STUDIES**  1. Is it clear in the study what is the 'cause' and what is the 'effect' (i.e. there is no confusion about which variable comes first)?  2. Were the participants included in any comparisons similar?  3. Were the participants included in any comparisons receiving similar treatment/care, other than the exposure or intervention of interest?  4. Was there a control group?  5. Were there multiple measurements of the outcome both pre and post the intervention/exposure?  6. Was follow up complete and if not, were differences between groups in terms of their follow up adequately described and analysed?  7. Were the outcomes of participants included in any comparisons measured in the same way?  8. Were outcomes measured in a reliable way?  9. Was appropriate statistical analysis used? |
| **CHECKLIST FOR EXPERIMENTAL STUDIES**  1. Was true randomization used for assignment of participants to treatment groups?  2. Was allocation to treatment groups concealed?  3. Were treatment groups similar at the baseline?  4. Were participants blind to treatment assignment?  5. Were those delivering treatment blind to treatment assignment?  6. Were outcomes assessors blind to treatment assignment?  7. Were treatment groups treated identically other than the intervention of interest?  8. Was follow up complete and if not, were differences between groups in terms of their follow up adequately described and analysed?  9. Were participants analysed in the groups to which they were randomized?  10. Were outcomes measured in the same way for treatment groups?  11. Were outcomes measured in a reliable way?  12. Was appropriate statistical analysis used?  13. Was the trial design appropriate, and any deviations from the standard RCT design (individual randomization, parallel groups) accounted for in the conduct and analysis of the trial? |

# COMPUTATION OF SCORES AND QUALITY CATEGORIES FOR THE JBI CRITICAL APPRAISAL TOOLS (CAT)

1 – The 4 options of answer of the CAT were given the following points

- Yes – 2 points
- No – 0 points
- Unclear – 1 point
- NA – missing

2 – The score is obtained by adding the points of each answer. The NA answer is not considered. Example for cross-sectional studies


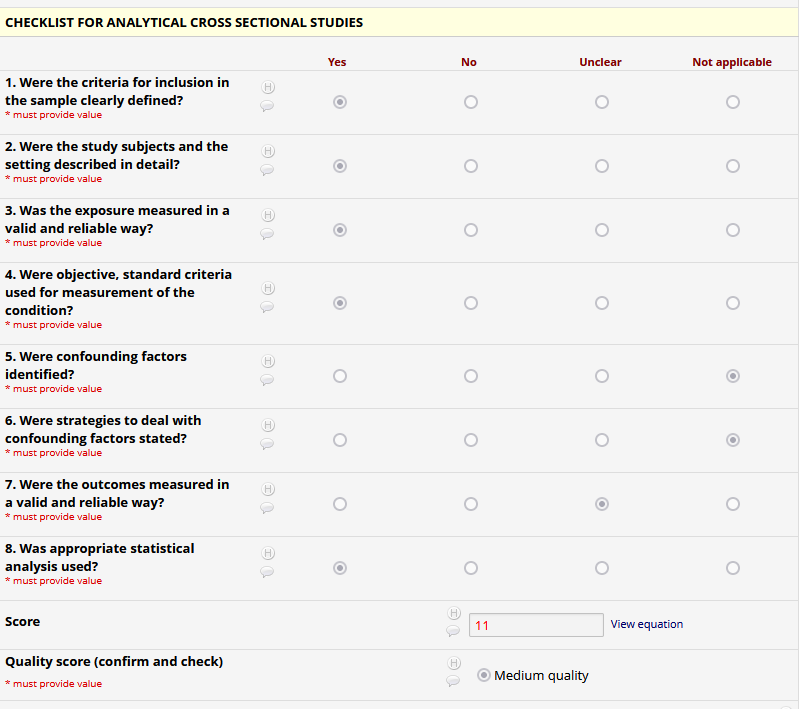


3 - For each CAT the maximum score was computed by multiplying the total number of item per the maximum score in each item (2 points per YES) as detailed in the table.

4 – Low quality was set at Q2 of the maximum score, MEDIUM quality was set between Q2+1 and Q3 and HIGH quality as Q4+1. In case an integer was not obtained the next integer was set as a cut-off (table).

| **CAT** | **Items** | **Maximum score** | **Scores and Quality** |
| --- | --- | --- | --- |
| Checklist for Analytical Cross-Sectional Studies | 8 | 16 | Low - ≤ 8  Medium - [9;12]  High - ≥ 13 |
| Checklist for Case Control Studies | 10 | 20 | Low - ≤ 10  Medium - [11;15]  High - ≥ 16 |
| Checklist for Cohort Studies | 11 | 22 | Low - ≤ 11  Medium - [12;17]  High - ≥ 18 |
| Checklist for Prevalence Studies | 9 | 18 | Low - ≤ 9  Medium - [10;14]  High - ≥ 15 |
| Checklist for Qualitative Research | 10 | 20 | Low - ≤ 10  Medium - [11;15]  High - ≥ 16 |
| Checklist for Quasi-Experimental Studies | 9 | 18 | Low - ≤ 10  Medium- [10;14]  High - ≥ 15 |
| Checklist for Randomized Controlled Trials | 13 | 26 | Low - ≤ 13  Medium - [14;20]  High - ≥ 21 |

# Quality of evidence assessment criteria

| **Study design** | **Quality of evidence** | **Lower if** | **Higher if** |
| --- | --- | --- | --- |
| Randomized Controlled Trials | High | **Risk of bias**  Medium risk of bias / quality according to CAT  **Inconsistency**  -1 serious  -2 very serious  **Imprecision**  -1 serious  -2 very serious  **Publication bias**  -1 likely  -2 very likely | **Large effect**  +1 Large  +2 Very large  **Dose response**  +1 evidence of a gradient  **All plausible confounding**  +1 Would reduce a demonstrated effect  +1 Would suggest a spurious effect when results show no effect |
| Quasi-Experimental Studies | Moderate |  |  |
| Cohort Studies | Low |  |  |
| Case Control Studies | Low |  |  |
| Analytical Cross-Sectional Studies | Low |  |  |
| Prevalence Studies | Low |  |  |
| Qualitative Research | Very low |  |  |

Adapted from: Guyatt G, Oxman AD, Akl EA, Kunz R, Vist G, Brozek J, et al. GRADE guidelines: 1. Introduction—GRADE evidence profiles and summary of findings tables. Journal of Clinical Epidemiology [Internet]. Elsevier; 2011 [cited 2022 Nov 20];64:383–94. Available from: https://www.jclinepi.com/article/S0895-4356(10)00330-6/fulltext#tbl2
